# Composition of PM Affects Acute Vascular Inflammatory and Coagulative Markers - The RAPTES Project

**DOI:** 10.1371/journal.pone.0058944

**Published:** 2013-03-13

**Authors:** Maciej Strak, Gerard Hoek, Krystal J. Godri, Ilse Gosens, Ian S. Mudway, René van Oerle, Henri M. H. Spronk, Flemming R. Cassee, Erik Lebret, Frank J. Kelly, Roy M. Harrison, Bert Brunekreef, Maaike Steenhof, Nicole A. H. Janssen

**Affiliations:** 1 Centre for Environmental Health (MGO), National Institute for Public Health and the Environment (RIVM), Bilthoven, The Netherlands; 2 Division of Environmental Epidemiology and Division of Toxicology, Institute for Risk Assessment Sciences (IRAS), Utrecht University, Utrecht, The Netherlands; 3 Environmental Research Group, MRC-HPA Centre for Environmental Health, King’s College London, London, United Kingdom; 4 Division of Environmental Health and Risk Management, School of Geography, Earth & Environmental Sciences, University of Birmingham, Birmingham, United Kingdom; 5 Laboratory for Clinical Thrombosis and Haemostasis, Department of Internal Medicine, Cardiovascular Research Institute Maastricht, Maastricht University Medical Center, Maastricht, The Netherlands; 6 Department of Environmental Sciences / Center of Excellence in Environmental Studies, King Abdulaziz University, Jeddah, Saudi Arabia; 7 Julius Centre for Health Sciences and Primary Care, University Medical Centre Utrecht, Utrecht, The Netherlands; Louisiana State University Health Sciences Center, United States of America

## Abstract

**Background:**

Exposure to ambient particulate matter (PM) has been associated with adverse cardiovascular effects in epidemiological studies. Current knowledge of independent effects of individual PM characteristics remains limited.

**Methods:**

Using a semi-experimental design we investigated which PM characteristics were consistently associated with blood biomarkers believed to be predictive of the risk of cardiovascular events. We exposed healthy adult volunteers at 5 different locations chosen to provide PM exposure contrasts with reduced correlations among PM characteristics. Each of the 31 volunteers was exposed for 5 h, exercising intermittently, 3–7 times at different sites from March to October 2009. Extensive on-site exposure characterization included measurements of PM mass and number concentration, elemental- (EC) and organic carbon (OC), trace metals, sulfate, nitrate, and PM oxidative potential (OP). Before and 2 h and 18 h after exposure we measured acute vascular blood biomarkers - C-reactive protein, fibrinogen, platelet counts, von Willebrand Factor, and tissue plasminogen activator/plasminogen activator inhibitor-1 complex. We used two-pollutant models to assess which PM characteristics were most consistently associated with the measured biomarkers.

**Results and Conclusion:**

We found OC, nitrate and sulfate to be most consistently associated with different biomarkers of acute cardiovascular risk. Associations with PM mass concentrations and OP were less consistent, whereas other measured components of the air pollution mixture, including PNC, EC, trace metals and NO_2_, were not associated with the biomarkers after adjusting for other pollutants.

## Introduction

Exposure to ambient particulate matter (PM) has been associated with cardiovascular health effects in epidemiological studies [1], [2]. These effects have been linked with exposure to ambient PM, mostly with concentrations of PM_2.5_ (particulate matter less than 2.5 µm in aerodynamic diameter; expressed in μg/m^3^). However, PM composition is variable in time and space and is strongly dependent on (local) sources of emission [3]. This heterogeneity in physiochemical composition has been shown to affect PM toxicity [4]. PM characteristics, like particle number concentration (expressed in particles/cm^3^), surface area, content of transition metals, organics, sulfate and nitrate, and biological components (e.g., endotoxin) have been proposed as possibly being responsible for observed adverse health effects. Recently, oxidative potential (OP) of PM has been suggested as a promising integrated and biologically meaningful measure to predict human health effects related to the PM mixture [5]. However, current knowledge of health effects of individual PM characteristics remains limited [2], [6], [7].

Current generally accepted hypotheses on the biological mechanisms of cardiovascular effects of PM exposure include the *indirect* pathway, where inhaled particles provoke lung cells to release pro-inflammatory mediators (e.g., cytokines, reactive oxygen species) that are consequently released into the circulation, and the *direct* pathway, with translocation of particles (i.e., ultrafines) or their constituents (e.g., soluble metals, organics) from the lungs into the circulation through the alveolar-capillary barrier [2]. Both pathways would cause systemic inflammation and (potentially) oxidative stress.

Over the recent years, several biomarkers of acute vascular response have been identified as contributors to the risk of cardiovascular disease, namely: C-reactive protein (CRP) as an acute-phase reactant and a sensitive systemic marker of inflammation and tissue damage [8]; fibrinogen as an acute-phase reactant in systemic inflammation [8] and involvement in blood coagulation [9]; platelets for their role in hemostasis and systemic inflammation [2], [10]; von Willebrand Factor (vWF) as an important factor in hemostasis and possible indicator of endothelial cell activation or damage [11]; and the complex between tissue plasminogen activator and plasminogen activator inhibitor-1 (tPA/PAI-1 complex) indicating impaired fibrinolytic activity [12]. Though not consistent across studies, these markers were associated with (typically 24-h average) ambient air pollution [2].

Often the limitations in study design are an obstacle in investigating the independent health effects of individual PM characteristics. In classical epidemiological studies a frequent limitation in disentangling independent health contributions is the high correlation between air pollutants [7], as well as pollutant-specific measurement error related to non-representativeness of a central monitoring site for personal exposure assessment. Those problems are avoided in controlled exposure studies in laboratory settings, but here the limitations often are individual air pollutants or defined mixtures (e.g., diesel engine exhaust) with high and constant concentrations, which may not be reflective of variable real-world exposures. The aim of the RAPTES project (“Risk of Airborne Particles: a Toxicological-Epidemiological Hybrid Study”) was to assess the independent contribution of specific PM characteristics to various health outcomes. We studied those by a short-term, semi-experimental exposure of healthy volunteers to ambient PM at selected real-world locations. At those locations we previously established substantial differences in PM characteristics [13] and investigated PM toxicity *in vitro* [14], as well as acute respiratory changes in human volunteers [15]. The current paper focuses on the associations of specific PM characteristics, including OP, with acute vascular changes in healthy human volunteers. We hypothesized that the changes would have a stronger and more consistent relationship with OP of PM, as a measure of integrated biological response of the total PM mixture, than with other measured characteristics.

## Materials and Methods

### Study Design

The RAPTES study design has been explained previously [15]. Briefly, we used a semi-experimental rather than a pure observational design to reduce exposure measurement error while still exposing the volunteers to real-world conditions. We exposed 31 healthy human volunteers to ambient PM at five locations in the Netherlands: an underground train station, a continuous and a stop-and-go traffic location, a farm and an urban background site. The locations all had different source characteristics, which resulted in increased exposure contrasts and reduced correlations between PM characteristics compared to a study conducted at a single site [13]. A detailed characterization of PM air pollution was performed on-site during exposure over 30 sampling days between March and October 2009. The participants were exposed at the five sampling locations multiple times, one site at a time, and each time pre- and post-exposure measurements were made to assess changes in biomarkers of acute vascular effects. Participants directly inhaled ambient air at each location; no air concentration or inhalation devices were used. Exposure contrast for the epidemiological analysis is derived from contrasts between sites and (temporal) variation within sites. Exposure always started around 9∶00–9∶30 and lasted for 5 h (Figure S1). Each hour participants cycled for 20 minutes on a stationary bicycle to increase the experimental dose and obtain a clearer health response, needed to disentangle the effects of different pollutants. The activity and 5-h exposure period was also selected to provide a contrast with the background exposure at the campus. To avoid potential carry-over effects from previous exposures, an individual’s visits to the sites were separated by a minimum of 14 days.

### Study Population

Volunteers were healthy, young, non-smoking students of Utrecht University, the Netherlands. We chose participants living at the campus to equalize and minimize exposure to traffic-related air pollution prior to the experimental exposure. Before the study, each participant attended a check-up by a physician to obtain medical clearance for participation. The study was approved by the ethics committee at University Medical Center Utrecht and written informed consent was provided by all participants.

### Exposure Measurements

We previously described in detail the measurements of exposure to air pollution on location and the instrumentation used [13]. Briefly, we determined mass concentrations and absorbance of PM_10_ and PM_2.5_ samples. The mass concentration of PM_2.5−10_ was calculated as the difference between PM_10_ and PM_2.5_. PM_10_ samples were analyzed for endotoxin content. Particle number concentration (PNC) between 0.007 µm and 3 µm was measured using a condensation particle counter. PM_2.5−10_ and PM_2.5_ samples collected with a high volume sampler were measured for the concentrations of elemental (EC) and organic carbon (OC), trace metals, e.g., iron (Fe), copper (Cu), nickel (Ni), vanadium (V) (both water-soluble and “total” acid-extracted fractions), and secondary inorganics (nitrate, sulfate). Gaseous pollutants concentrations (O_3_, NO_2_, NO_X_) were measured using real-time monitors. PM_2.5−10_, PM_2.5−0.18_ and PM_0.18_ samples were collected using the Micro-Orifice cascade Impactor (MOUDI, model M100-NR, MSP Corporation, Shoreview, MN, USA) and their OP was determined *in vitro* by the extent of ascorbate (OP^AA^) and reduced glutathione (OP^GSH^) depletion [16] and the sum of both metrics is presented as OP^TOTAL^, expressed per m^3^. We report absorbance, nitrate and sulfate in the fine fraction, whereas the individual PM fraction concentrations of OP and trace metals were aggregated.

To minimize exposure during transport of participants between the collection point and the sampling locations, we equipped a minibus with a custom-made cabin air filter. To estimate the traffic-related air pollution during transport, we measured the PNC in the minibus during each commute, as described previously [15].

### Clinical Measurements

Venous blood was collected into K_2_-EDTA, 3.2% (w/v) citrate and serum tubes (BD) through vena punction using 21 gauge needles before exposure (t = 0), 2 h after exposure (t = 9), and the next morning (t = 25) at the collection point located at the Utrecht University campus. Short after the collection (at t = 0 and t = 25) or the next morning (refrigerated after t = 9) EDTA plasma samples were transported to an external, commercial laboratory that performed the complete blood cell counts. Serum for analysis of high-sensitivity CRP (hs-CRP) and citrate plasma for analysis of fibrinogen, vWF and tPA/PAI-1 complex were stored at −80°C and analyzed at the end of the sampling campaign. Hs-CRP was measured in serum using CRP Vario turbidimetric immunoassay (Sentinel, Milan, Italy) with clinical chemistry analyzer (ARCHITECT; Abbott Laboratories, Abbott Park, IL, USA); platelets were analyzed as part of complete blood cell count using an automated hematology analyzer (CELL-DYN; Abbott Laboratories, Abbott Park, IL, USA); fibrinogen was measured on a Behring Coagulation System (BCS) with Multifibren U kit (Siemens Healthcare Diagnostics, Marburg, Germany) using a modification of the Clauss method; vWF antigen was measured on a BCS with vWF:ag immunoturbidimetric determination kit (Siemens Healthcare Diagnostics, Marburg, Germany); tPA/PAI-1 complex was measured using Technoclone tPA/PAI-1 Complex ELISA Reagent Kit (Technoclone, Vienna, Austria) according to the manufacturers instructions. The coefficients of variation (CV) for hs-CRP, fibrinogen, vWF and tPA/PAI-1 complex were <1.25%, <3%, <2% and <11%, respectively.

At t = 0 and t = 25 participants filled in a questionnaire reporting e.g., time spent in traffic, medication use and respiratory symptoms during the last 24 h.

### Statistical Analyses

We analyzed the associations between air pollution concentrations measured during exposure at the sampling locations and the difference in acute vascular markers between post- (t = 9, t = 25) and pre-exposure (t = 0) for each sampling day using mixed linear regression. We used mixed models to account for the influence of repeated observations per subject (using compound symmetry of the residuals). Pre- and post-exposure levels of biomarkers were log-transformed to stabilize outliers. The associations between vWF and PM characteristics were driven by one obvious extreme outlier which influence was not diminished by log-transformation. Since the outlier decreased several otherwise stronger and significant estimates, we decided to report the vWF associations from the models where this outlier was excluded. As independent variables, we used the 5-h average concentrations of air pollutants measured at the locations. We first specified single-pollutant models. Then, to disentangle the individual effects of different pollutants, we specified two-pollutant models with all possible combinations of measured pollutants. We specified two-pollutant models because, due to the experimental nature of the study, exposure measurement error is much less important compared to pure observational studies in determining which of the two exposure variables has the strongest association with health. Further, we did not perform a factor analysis to group correlated pollutants because of an insufficient amount of observations available for such analysis (30 days).

The two-pollutant models are our main models, as in these the independent associations of particle components are assessed. We report first the results for single-pollutant models, to be able to compare with previous studies. If we only showed two-pollutant model results with e.g. no significant associations with PM_2.5_ then we would not be able to distinguish between the scenario of no associations with PM_2.5_ at all (in single- and two-pollutant models) and the scenario of significant association with PM_2.5_ (in single-pollutant model) that was explained by a specific component when included in two-pollutant model.

Effect estimates and confidence intervals (CI) are presented as percentage change over a study population mean of the baseline (t = 0) values expressed per changes in interquartile ranges (IQR) for the outdoor locations. We largely focus on the results of two-pollutant models. We did not interpret models with Spearman’s R larger than 0.7 between two pollutants as those were considered highly correlated. Inclusion of highly correlated predictors in regression models may result in unstable regression coefficients (co-linearity). Since we defined a large number of models, we focus on the consistency of significant associations rather than single isolated significant associations. Due to a substantial difference in some exposures between outdoor locations and the underground location [13], we also analyzed data separately for the outdoor sites.

As potential confounders in the analysis, we adjusted for temperature and relative humidity measured at the location during sampling, and season (before or after the start date of the calendar summer), as in our analysis of respiratory effects [15]. We additionally adjusted for use of oral contraceptives and the use of oral contraceptives on the sampling day or a day before, since those may affect our biomarkers [17], [18]. The impact of influential observations on the regression results was assessed by comparing effect estimates with and without 1% of observations with the highest Cook’s Distance value. Data analyses were carried out using SAS 9.2 (SAS Institute, Cary, NC).

## Results

We obtained a total of 170 observations from 31 participants (Table 1). Participants were exposed between three and seven times at different sites, and each person visited the underground train station at least once – in total we obtained 45 observations at the underground and 28–37 at the other sites. Baseline markers mean levels agreed closely with a recent Dutch study in healthy adults [19].

**Table 1:** Population characteristics and baseline (t = 0) levels of blood biomarkers.

Characteristic	Value
**Age (years)**	22 (19–26)
**Sex, n (%)**	
** Female**	21 (68%)
** Male**	10 (32%)
**Body Mass Index (kg/m^2^)**	22.3 (17.0–32.0)
**Hs-CRP (mg/L)**	1.00 (0.10–14.48)
**Fibrinogen (g/L)**	3.02 (1.43–5.19)
**Platelet counts (10^9^/L)**	267.79 (130.00–416.00)
**vWF (% of normal)**	89.41 (37.72–199.64)
**tPA/PAI-1 complex (ng/mL)**	2.81 (0.08–27.39)

Unless otherwise stated, values are means (ranges), or geometric means (ranges) for the biomarkers.

N = 170, except vWF (N = 169) and tPA/PAI-1 complex (N = 168).

### Concentrations of Air Pollutants

Nearly every PM characteristic, especially levels of Fe, Cu and OP^TOTAL^, had substantially higher levels at the underground train station (Table S1 in Supporting Information). The highest PNC was at the continuous traffic site. Endotoxin levels were the highest at the farm. Variability within sites was smaller than between sites.

Correlations between air pollution concentrations are shown in Table S2 and we discussed them more extensively in our previous publication [13]. Briefly, we found PM_10_ and PM_2.5_ to be highly correlated with each other, as well as with absorbance, EC, OC, trace metals, OP^TOTAL^, but less so with PNC. O_3_ showed a strong negative correlation with several PM characteristics. The high correlations decreased considerably after we excluded the measurements from the underground train station. Due to this exclusion, we observed substantially stronger correlations between PNC and EC or absorbance (whereas the correlations between PNC and PM_10_ or PM_2.5_ remained low). Overall, both in the “all sites” and the “outdoor-only sites” dataset, the correlations between several PM characteristics were sufficiently low to investigate their independent effects on human health in two-pollutant models.

### Associations 2 h after Exposure

In the single-pollutant models, hs-CRP (Table S4), fibrinogen (Table S5) and tPA/PAI-1 complex (Table S6) were not associated with any of the PM characteristics. There was a negative association of platelet counts with nitrogen oxides in the outdoor-only dataset (Table S7). NO_2_ and OC (fine) were positively associated with vWF in the complete dataset and the latter association was also present in the outdoor-only models (Table S8).

Generally, we did not observe strong and consistent associations 2 h after exposure, therefore we did not analyze the two-pollutant models for this time point.

### Associations in the Following Morning

#### Air pollution and C-reactive protein

In single-pollutant models, an increase in hs-CRP was associated with an increase in PM mass concentrations, OP, OC (fine) and transition metals, and negatively associated with O_3_ (Table S4). Those associations were not present in the outdoor-only data subset.

In two-pollutant models, none of the PM characteristics remained significant after including each of the other co-pollutants (Table 2 and Table S9). We use the term “consistent” if a component is (borderline) significantly associated after adjusting for all other components (shown in the row per component) and the effect estimates do not change substantially compared to the single-pollutant estimate. However, OP and OC (fine) were fairly consistently associated with increase in hs-CRP after inclusion of most other PM characteristics (Table 2 and Table S9). OC (fine) had (borderline) significant associations in 16 of the models shown in the OC (fine) row and effect estimates did not deviate much (4.6% to 6.9%) from the single-pollutant estimate (6.7%). In contrast, e.g. PM_10_ remained significant in seven models but the effect estimate became negative after adjusting for Fe and Cu with a borderline significant association in the single-pollutant model, whereas the associations with PM_2.5_ (also borderline significant in the single-pollutant model) decreased and became non-significant after adjusting for EC, some trace metals and gaseous pollutants (Table S9). In the outdoor-only models no consistent associations were found (Table S13).

**Table 2:** Associations between exposure to air pollution and percentage changes (25 h post-pre) in hs-CRP (all sites).

	IQR	Single-pollutant associations	Two-pollutant associations									
			PM_10_	PM_2.5_	PNC	EC(F)	OC(F)	OC(C)	NO_3_^–a^	SO_4_^2–a^	OP^TOTAL^	NO_2_
**PM_10_**	13.50	0.74*(−0.01, 1.50)		*0.19* *(−2.80, 3.26)*	0.79**(0.03, 1.56)	1.46*(−0.24, 3.19)	*0.36* *(−0.57, 1.30)*	*1.01* * *(−0.12, 2.14)*	0.72*(*−*0.05, 1.48)	0.77**(0.02, 1.53)	*−0.71* *(−2.80, 1.43)*	0.63(*−*0.14, 1.41)
**PM_2.5_**	11.54	1.74*(0.00, 3.51)	*1.32* *(−5.49, 8.62)*		1.81**(0.07, 3.59)	2.54(*−*0.64, 5.83)	*0.76* *(−1.53, 3.10)*	2.46*(*−*0.15, 5.14)	1.71*(*−*0.13, 3.58)	1.74*(0.00, 3.51)	*0.13* *(−3.48, 3.89)*	1.45(*−*0.36, 3.28)
**PNC**	32,906	*−*4.31(*−*14.35, 6.92)	*−*5.75(*−*15.66, 5.32)	*−*5.23(*−*15.15, 5.85)		*−*9.91(*−*20.56, 2.17)	*−*4.89(*−*14.78, 6.16)	*−*2.23(*−*12.51, 9.26)	*−*3.46(*−*14.05, 8.44)	*−*3.49(*−*13.91, 8.19)	*−*8.95(*−*18.84, 2.13)	*−*11.23*(*−*21.75, 0.71)
**EC(F)**	4.35	4.23(*−*2.05, 10.91)	*−*6.30(*−*18.50, 7.72)	*−*3.30(*−*13.63, 8.28)	7.24*(*−*0.07, 15.08)		1.30(*−*5.46, 8.54)	3.82(*−*3.50, 11.70)	4.53(*−*1.80, 11.27)	4.98(*−*1.47, 11.86)	*−8.79* *(−19.03, 2.74)*	2.63(*−*4.01, 9.73)
**OC(F)**	1.82	6.68**(0.81, 12.89)	*5.00* *(−2.14, 12.67)*	*4.95* *(−2.69, 13.20)*	6.81**(0.92, 13.04)	6.13*(*−*0.40, 13.08)		6.94**(0.60, 13.69)	6.94**(0.52, 13.78)	6.48**(0.56, 12.74)	5.52(*−*1.93, 13.54)	5.69*(*−*0.48, 12.24)
**OC(C)**	0.79	1.64(*−*2.17, 5.60)	*−2.18* *(−7.65, 3.63)*	*−*2.40(*−*7.87, 3.40)	1.61(*−*2.22, 5.58)	0.36(*−*4.11, 5.05)	*−*0.20(*−*4.25, 4.03)		1.11(*−*3.06, 5.45)	1.72(*−*2.10, 5.68)	*−0.79* *(−5.55, 4.21)*	1.18(*−*2.61, 5.12)
**NO_3_^–a^**	5.19	1.89(*−*3.21, 7.26)	1.21(*−*3.89, 6.57)	0.30(*−*4.96, 5.86)	1.41(*−*3.90, 7.01)	2.28(*−*2.86, 7.69)	*−*0.54(*−*5.92, 5.15)	1.71(*−*3.81, 7.55)		0.97(*−*5.42, 7.79)	3.01(*−*2.11, 8.39)	0.79(*−*4.44, 6.30)
**SO_4_^2–a^**	2.99	2.51 (*−*3.59, 9.00)	3.07(*−*3.06, 9.58)	2.53(*−*3.55, 8.98)	2.05(*−*4.21, 8.71)	3.53(*−*2.75, 10.22)	1.64(*−*4.41, 8.08)	2.92(*−*3.13, 9.35)	1.79(*−*5.87, 10.07)		3.05(*−*2.96, 9.43)	1.94(*−*4.14, 8.41)
**OP^TOTAL^**	38.71	0.28**(0.05, 0.52)	*0.51* *(−0.20, 1.23)*	*0.27* *(−0.27, 0.81)*	0.31**(0.07, 0.55)	0.61** *(0.13, 1.08)*	0.13(*−*0.18, 0.45)	*0.30* * *(−0.02, 0.61)*	0.27**(0.04, 0.51)	0.29**(0.05, 0.53)		0.27**(0.03, 0.51)
**NO_2_**	10.54	10.81(*−*2.30, 25.67)	8.11 (*−*4.99, 23.02)	7.56(*−*5.65, 22.61)	18.64**(2.70, 37.05)	8.60(*−*5.24, 24.46)	6.33(*−*6.94, 21.50)	12.79*(*−*0.59, 27.96)	10.21(*−*3.37, 25.70)	10.29(*−*2.87, 25.23)	4.44(*−*8.30, 18.96)	

a measured in PM_2.5_.

* p<0.10, ** p<0.05.

“C” denotes the coarse, whereas “F” the fine PM fraction.

Italics indicate Spearman’s R above 0.7. In each row effect estimates (95% confidence intervals) for the indicated pollutant are presented. All models are adjusted for the use of oral contraceptives and the use of oral contraceptives on the sampling day or the day before, temperature, relative humidity, season and adjustment pollutant (indicated in the header of each column). For example, in the single-pollutant model PM_10_ has the effect estimate of 0.74 (*−*0.01, 1.50), whereas after adjusting for PNC, the effect estimate is 0.79 (0.03, 1.56). Estimates are percentage increases above population-average baseline expressed per outdoor-sites IQR. N = 170, except all models including OP where N = 153 and all models including EC (C) and OC (C) where N = 166.

#### Air pollution and fibrinogen

Increases in nitrate, sulfate, OC (coarse) and water-soluble Cu were associated with increases in fibrinogen in single-pollutant models (Table S5). Positive associations with nitrate and sulfate were also found in the outdoor-only models.

Nitrate and sulfate were most consistently associated with fibrinogen in two-pollutant models (Table 3 and Table S10). In the outdoor-only subset, the associations with both sulfate and nitrate were consistently robust, but decreased somewhat after adjusting for each other (and sulfate after adjusting for OP^AA^) (Table S14).

**Table 3:** Associations between exposure to air pollution and percentage changes (25 h post-pre) in fibrinogen (all sites).

	IQR	Single-pollutant associations	Two-pollutant associations									
			PM_10_	PM_2.5_	PNC	EC(F)	OC(F)	OC(C)	NO_3_^–a^	SO_4_^2–a^	OP^TOTAL^	NO_2_
**PM_10_**	13.50	0.10(*−*0.04, 0.25)		*−0.18* *(−0.75, 0.40)*	0.11(*−*0.03, 0.26)	0.30*(*−*0.02, 0.62)	*0.07* *(−0.11, 0.25)*	*0.02* *(−0.17, 0.21)*	0.08(*−*0.06, 0.23)	0.11(*−*0.03, 0.25)	*0.06* *(−0.37, 0.50)*	0.11(−0.04, 0.25)
**PM_2.5_**	11.54	0.27(−0.06, 0.60)	*0.68* *(−0.65, 2.02)*		0.28*(*−*0.04, 0.62)	0.59*(*−*0.01, 1.19)	*0.22* *(−0.22, 0.66)*	0.14(*−*0.30, 0.57)	0.18(*−*0.16, 0.53)	0.26(*−*0.06, 0.59)	*0.33* *(−0.42, 1.08)*	0.28(*−*0.06, 0.63)
**PNC**	32,906	*−*0.92(*−*2.98, 1.19)	*−*1.12(*−*3.19, 0.99)	*−*1.06(*−*3.11, 1.05)		*−*1.62(*−*3.95, 0.76)	*−*0.99(*−*3.05, 1.12)	*−*0.93(*−*2.75, 0.92)	*−*0.35(*−*2.50, 1.85)	*−*0.46(*−*2.55, 1.69)	*−*1.27(*−*3.56, 1.07)	*−*1.40(*−*3.77, 1.04)
**EC(F)**	4.35	0.40(*−*0.78, 1.59)	*−*1.82(*−*4.38, 0.80)	*−*1.36(*−*3.45, 0.77)	0.84(*−*0.51, 2.21)		0.06(*−*1.26, 1.39)	*−*0.23(*−*1.45, 0.99)	0.52(*−*0.66, 1.71)	0.66(*−*0.53, 1.86)	*−1.33* *(−3.70, 1.10)*	0.40(*−*0.88, 1.70)
**OC(F)**	1.82	0.73(*−*0.36, 1.82)	*0.41* *(−0.94, 1.77)*	*0.24* *(−1.20, 1.70)*	0.75(*−*0.34, 1.85)	0.70(*−*0.51, 1.94)		0.47(*−*0.56, 1.51)	0.34(*−*0.84, 1.53)	0.56(*−*0.53, 1.65)	0.66(*−*0.84, 2.18)	0.77(*−*0.39, 1.94)
**OC(C)**	0.79	0.64*(0.00, 1.29)	*0.56* *(−0.41, 1.54)*	0.41(*−*0.56, 1.40)	0.62*(*−*0.02, 1.27)	0.72*(*−*0.05, 1.49)	0.51(*−*0.19, 1.22)		0.39(*−*0.32, 1.10)	0.64**(0.01, 1.27)	*0.71* *(−0.17, 1.59)*	0.64*(*−*0.01, 1.30)
**NO_3_** *−* **^a^**	5.19	0.98**(0.01, 1.96)	0.90*(*−*0.08, 1.89)	0.81(*−*0.22, 1.84)	0.94*(*−*0.08, 1.96)	1.03**(0.05, 2.01)	0.86(*−*0.21, 1.94)	0.77(*−*0.18, 1.72)		0.54(*−*0.69, 1.78)	0.98*(*−*0.06, 2.02)	1.03**(0.02, 2.06)
**SO_4_^2^** *−* **^a^**	2.99	1.33** (0.11, 2.57)	1.40**(0.17, 2.64)	1.31**(0.08, 2.54)	1.27**(0.02, 2.55)	1.47**(0.22, 2.73)	1.23*(*−*0.01, 2.49)	1.34**(0.28, 2.41)	0.90(*−*0.66, 2.49)		1.21*(*−*0.07, 2.51)	1.34**(0.10, 2.59)
**OP^TOTAL^**	38.71	0.04(*−*0.01, 0.08)	*0.02* *(−0.13, 0.16)*	*−0.01* *(−0.12, 0.10)*	0.04(*−*0.01, 0.09)	*0.08* *(−0.02, 0.18)*	0.02(*−*0.05, 0.08)	*0.01* *(−0.05, 0.06)*	0.03(*−*0.02, 0.08)	0.04(*−*0.01, 0.08)		0.04(*−*0.01, 0.09)
**NO_2_**	10.54	0.29(*−*2.09, 2.73)	*−*0.11(*−*2.55, 2.38)	*−*0.29(*−*2.75, 2.24)	1.10(*−*1.67, 3.95)	*−*0.02(*−*2.59, 2.62)	*−*0.28(*−*2.81, 2.30)	*−*0.01(*−*2.12, 2.15)	*−*0.42(*−*2.86, 2.09)	*−*0.05(*−*2.42, 2.37)	*−*0.35(*−*2.96, 2.33)	

For explanation see Table 2.

The associations with sulfate decreased and were non-significant (particularly in the complete dataset) after excluding 1% observations with the highest Cook’s Distance.

#### Air pollution and platelets counts

In single-pollutant models there were positive associations of platelet counts with OC (coarse) and nitrate and negative associations with nitrogen oxides (Table S7). The positive OC (coarse) and nitrate associations were also present in the outdoor-only models, along with positive associations with PM_10_ and PM_2.5_.

In two-pollutant models, a consistent and robust positive association was found for platelet counts with OC (coarse) (Table 4 and Table S11). A fairly consistent positive association was also found for nitrate. Negative associations with NO_X_ remained, except for adjusting for PNC when NO_X_ became somewhat less strong and less significant. In the outdoor-only set, none of the PM characteristics remained significant after adjusting for all other pollutants. Fairly consistent associations of a similar magnitude, though less robust than in the complete dataset, were found for OC (coarse), nitrate, PM_10_ and PM_2.5_, with the two former being more stable (Table S15). The negative associations with NO_X_ found in the outdoor-only dataset were less consistent than in the complete dataset.

**Table 4:** Associations between exposure to air pollution and percentage changes (25 h post-pre) in platelet counts (all sites).

	IQR	Single-pollutant associations	Two-pollutant associations									
			PM_10_	PM_2.5_	PNC	EC(F)	OC(F)	OC(C)	NO_3_^–a^	SO_4_^2–a^	OP^TOTAL^	NO_2_
**PM_10_**	13.50	0.04(*−*0.07, 0.14)		*0.12* *(−0.31, 0.55)*	0.05(*−*0.06, 0.16)	0.23*(0.00, 0.47)	*0.07* *(−0.06, 0.20)*	−0.17** *(−0.31, −0.02)*	0.03(*−*0.08, 0.13)	0.04(*−*0.06, 0.15)	*0.17* *(−0.14, 0.48)*	0.07(*−*0.04, 0.17)
**PM_2.5_**	11.54	0.07(*−*0.17, 0.32)	*−0.19* *(−1.17, 0.80)*		0.09(*−*0.15, 0.34)	0.30(*−*0.14, 0.75)	*0.17* *(−0.16, 0.49)*	*−*0.39**(*−*0.72, *−*0.05)	0.00(*−*0.25, 0.26)	0.08(*−*0.17, 0.32)	*0.08* *(−0.45, 0.62)*	0.15(*−*0.10, 0.40)
**PNC**	32,906	*−*1.15(*−*2.69, 0.40)	*−*1.26(*−*2.80, 0.32)	*−*1.21(*−*2.75, 0.36)		*−*1.44(*−*3.20, 0.35)	*−*1.15(*−*2.69, 0.41)	*−*1.15(*−*2.68, 0.40)	*−*0.75(*−*2.36, 0.88)	*−*1.09(*−*2.67, 0.51)	*−*0.76(*−*2.42, 0.94)	*−*0.51(*−*2.29, 1.30)
**EC(F)**	4.35	*−*0.08(*−*0.95, 0.80)	*−*1.77*(*−*3.66, 0.16)	*−*0.98(*−*2.55, 0.61)	0.32(*−*0.68, 1.33)		*−*0.03(*−*0.99, 0.95)	*−*1.06**(*−*2.02, *−*0.08)	0.03(*−*0.84, 0.90)	*−*0.02(*−*0.91, 0.88)	*−0.58* *(−2.26, 1.14)*	0.29(*−*0.65, 1.23)
**OC(F)**	1.82	*−*0.12(*−*0.91, 0.69)	*−0.43* *(−1.41, 0.56)*	*−0.47* *(−1.51, 0.59)*	*−*0.10(*−*0.90, 0.70)	*−*0.11(*−*0.99, 0.79)		*−*0.65(*−*1.47, 0.18)	*−*0.50(*−*1.35, 0.36)	*−*0.14(*−*0.95, 0.67)	*−*0.31(*−*1.37, 0.75)	0.11(*−*0.72, 0.95)
**OC(C)**	0.79	0.79**(0.25, 1.33)	1.44** *(0.66, 2.23)*	1.44**(0.67, 2.21)	0.78**(0.24, 1.32)	1.16**(0.53, 1.79)	0.96**(0.38, 1.54)		0.63**(0.04, 1.23)	0.79**(0.25, 1.34)	1.06** *(0.36, 1.77)*	0.84**(0.30, 1.37)
**NO_3_** *−* **^a^**	5.19	0.74**(0.02, 1.47)	0.72*(*−*0.01, 1.46)	0.74*(*−*0.02, 1.51)	0.64*(*−*0.12, 1.41)	0.75**(0.02, 1.48)	0.92**(0.14, 1.71)	0.52(*−*0.26, 1.31)		0.90*(0.01, 1.81)	0.37(*−*0.39, 1.13)	0.98**(0.25, 1.73)
**SO_4_^2^** *−* **^a^**	2.99	0.29(*−*0.58, 1.17)	0.33(*−*0.55, 1.21)	0.30(*−*0.57, 1.18)	0.18(*−*0.71, 1.07)	0.29(*−*0.60, 1.19)	0.31(*−*0.57, 1.19)	0.41(*−*0.44, 1.26)	*−*0.33(*−*1.39, 0.74)		0.02(*−*0.87, 0.91)	0.39(*−*0.48, 1.26)
**OP^TOTAL^**	38.71	0.02(*−*0.02, 0.05)	*−0.04* *(−0.14, 0.06)*	*0.01* *(−0.07, 0.08)*	0.02(*−*0.02, 0.05)	*0.04* *(−0.03, 0.10)*	0.02(*−*0.02, 0.07)	*−0.03 (−0.07, 0.02)*	0.01(*−*0.02, 0.05)	0.02(*−*0.02, 0.05)		0.02(*−*0.02, 0.05)
**NO_2_**	10.54	*−*1.69*(*−*3.40, 0.06)	*−*1.93**(*−*3.68, *−*0.14)	*−*1.98**(*−*3.75, *−*0.17)	*−*1.40(*−*3.39, 0.64)	*−*1.91**(*−*3.76, *−*0.02)	*−*1.76*(*−*3.55, 0.07)	*−*1.83**(*−*3.51, *−*0.11)	*−*2.28**(*−*4.01, *−*0.52)	*−*1.77**(*−*3.50, *−*0.02)	*−*1.24(*−*3.09, 0.64)	

For explanation see Table 2.

#### Air pollution and von Willebrand Factor

Changes in vWF in single-pollutant models were positively associated with PM mass concentrations, EC (coarse), OC (fine), OP^GSH^ and OP^TOTAL^ (Table S8). In the outdoor subset, only PM_2.5_ and OC (fine) were positively associated.

In the two-pollutant models, no single component was consistently associated with vWF (Table 5 and Table S12). Associations with OC (fine) were most consistent, whereas the associations with PM mass concentrations were not consistent after adjusting for a range of components (Table S12). In the outdoor-only subset, the association with OC (fine) was consistent and robust and only decreased somewhat after adjusting for OP^AA^ (Table S16). We also saw a fairly consistent positive association with PM_2.5_.

**Table 5:** Associations between exposure to air pollution and percentage changes (25 h post-pre) in von Willebrand Factor (all sites).

	IQR	Single-pollutant associations	Two-pollutant associations									
			PM_10_	PM_2.5_	PNC	EC(F)	OC(F)	OC(C)	NO_3_^–a^	SO_4_^2–a^	OP^TOTAL^	NO_2_
**PM_10_**	13.50	0.22**(0.02, 0.41)		*−0.32* *(−1.09, 0.46)*	0.22**(0.02, 0.42)	0.31(*−*0.12, 0.75)	*−0.04* *(−0.27, 0.20)*	*0.07* *(−0.22, 0.37)*	0.21**(0.01, 0.41)	0.13(*−*0.07, 0.33)	*0.02* *(−0.55, 0.60)*	0.20*(*−*0.01, 0.40)
**PM_2.5_**	11.54	0.58**(0.12, 1.03)	*1.29* *(−0.51, 3.12)*		0.58**(0.12, 1.03)	0.78*(*−*0.04, 1.60)	*−0.06* *(−0.64, 0.53)*	0.32(*−*0.36, 1.00)	0.55**(0.07, 1.03)	0.35(*−*0.11, 0.81)	*0.34* *(−0.65, 1.34)*	0.53**(0.06, 1.00)
**PNC**	32,906	*−*0.04(*−*2.80, 2.80)	0.16(*−*2.70, 3.09)	0.28(*−*2.56, 3.20)		*−*0.40(*−*3.53, 2.83)	*−*0.13(*−*2.87, 2.69)	0.87(*−*2.05, 3.87)	0.40(*−*2.48, 3.38)	0.37(*−*2.60, 3.42)	*−*1.40(*−*4.47, 1.78)	*−*0.73(*−*3.88, 2.52)
**EC(F)**	4.35	0.32(*−*1.27, 1.94)	*−*0.91(*−*4.42, 2.73)	*−*0.87(*−*3.73, 2.08)	0.44(*−*1.37, 2.28)		*−*0.40 (*−*2.14, 1.36)	0.06(*−*1.83, 2.00)	1.54*(*−*0.12, 3.22)	0.88(*−*0.81, 2.59)	*−2.13 (−5.21, 1.04)*	0.12(–1.58, 1.85)
**OC(F)**	1.82	1.81**(0.26, 3.38)	*1.51* *(−0.29, 3.34)*	*1.46* *(−0.47, 3.44)*	1.34*(*−*0.11, 2.81)	1.50*(*−*0.12, 3.13)		1.68**(0.05, 3.33)	1.69*(0.01, 3.41)	1.24(*−*0.29, 2.80)	0.98(*−*1.03, 3.02)	1.30*(*−*0.23, 2.86)
**OC(C)**	0.79	0.48(*−*0.53, 1.50)	*−0.05* *(−1.55, 1.46)*	*−*0.29(*−*1.78, 1.22)	0.50(*−*0.52, 1.53)	0.20(*−*0.97, 1.38)	0.03(*−*1.05, 1.13)		*−*0.02(*−*1.09, 1.07)	0.13(*−*0.89, 1.17)	*0.56* *(−0.78, 1.92)*	0.17(*−*0.81, 1.16)
**NO_3_** *−* **^a^**	5.19	0.66(*−*0.63, 1.97)	0.59(–0.74, 1.95)	0.29(*−*1.10, 1.70)	0.72(*−*0.63, 2.09)	0.92(*−*0.42, 2.28)	0.25(*−*1.21, 1.74)	0.73(*−*0.70, 2.19)		0.55(*−*1.19, 2.33)	0.87(*−*0.54, 2.30)	0.57(*−*0.78, 1.94)
**SO_4_^2^** *−* **^a^**	2.99	0.93(*−*0.91, 2.80)	0.99(*−*0.86, 2.88)	0.88(*−*0.97, 2.76)	0.97(*−*0.91, 2.89)	1.11(*−*0.78, 3.03)	0.67(*−*1.19, 2.56)	1.00(*−*0.86, 2.88)	0.46(*−*1.89, 2.87)		1.04(*−*0.85, 2.97)	0.80(*−*1.07, 2.70)
**OP^TOTAL^**	38.71	0.06*(*−*0.01, 0.12)	*0.01* *(−0.18, 0.21)*	*−0.02* *(−0.17, 0.12)*	0.06*(0.00, 0.13)	*0.10* *(−0.03, 0.22)*	0.03(*−*0.06, 0.12)	*−0.01* *(−0.09, 0.08)*	0.06(*−*0.01, 0.12)	0.03(*−*0.03, 0.10)		0.06*(*−*0.01, 0.12)
**NO_2_**	10.54	1.19(*−*2.00, 4.49)	1.79(*−*1.56, 5.26)	1.52(*−*1.86, 5.01)	1.61(*−*2.08, 5.45)	1.10(*−*2.33, 4.66)	0.25(*−*3.09, 3.70)	1.41(*−*1.86, 4.78)	0.78(*−*2.55, 4.22)	1.48(*−*1.90, 4.98)	0.53(*−*3.03, 4.21)	

For explanation see Table 2.

#### Air pollution and tPA/PAI-1 complex

In single-pollutant models, PM mass concentrations, EC, absorbance, total Fe and nitrogen oxides were positively associated with increase in the tPA/PAI-1 complex, whereas O_3_ had an opposite association (Table S6). The latter one is the only association that was also present in the outdoor-only models.

In the two-pollutant models, no single component was consistently associated with increase in the tPA/PAI-1 complex (Table S17). Strong consistent negative associations with O_3_ were found, possibly due to high negative correlation with EC. In the outdoor-sites models, O_3_ was similarly strong, though the estimates after adjusting for absorbance and EC (coarse) lost statistical significance (Table S18). The associations with water-soluble Ni were driven by 1% of influential observations with the highest Cook’s Distance and were not present anymore after those were removed.

We log-transformed the tPA/PAI-1 complex levels for consistency with other endpoints, but even though the transformation did not affect normality, it resulted in disappearance of fairly consistent associations with absorbance, EC, OP^AA^ and NO_2_ seen in the “all sites” dataset before log-transformation (Table S19).

### Sensitivity Analyses

Exposure of participants to PNC during transport was not associated with changes in acute vascular markers investigated. Inclusion of in-transport exposure did not affect the reported associations with our experimental exposures (data not shown).

Deletion of 1% observations with the highest Cook’s Distances generally did not affect the effect estimates, except for the aforesaid associations with vWF and other associations mentioned in the text.

Further adjustment for infections/colds at baseline, did not affect the reported associations with air pollution (data not shown).

## Discussion

We investigated the acute vascular effects in a panel of healthy, young volunteers semi-experimentally exposed for 5 h to ambient air pollution with contrasting PM characteristics. Most associations were observed in the next morning rather than 2 h after exposure. In two-pollutant models we found associations of OC (fine) and OP with hs-CRP; sulfate and nitrate with fibrinogen; OC (coarse) and nitrate with platelet counts; and OC (fine) with vWF. We also observed unexpected negative associations with NO_2_/NO_X_ (platelet counts) and O_3_ (tPA/PAI-1 complex).

Our study substantially adds to the existing limited evidence of the effects of particulate matter air pollution on the biomarkers of cardiovascular risk. Compared to previous epidemiological studies which often use 24-hour average exposures, this study shows that five-hour exposure with exercise is sufficient to elicit biological responses. The semi-experimental design of our study is a strength when compared to observational studies as bias due to exposure measurement error and confounding is reduced. This study further adds to the very scarce evidence of the effects of different particle components. We have documented that associations with specific components (OC, nitrate, sulfate) were stronger than with widely used, legally regulated, PM mass metrics.

### PM Characteristics and Components

In single-pollutant models many significant associations of PM mass and composition with acute vascular biomarkers were observed. Those were, however, not robust against adjustment for co-pollutants. In two-pollutant models, with the exception of fairly consistent associations in the ”outdoor sites” models with platelets and von Willebrand Factor, the associations with PM mass concentrations generally disappeared. This supports the notion that particle composition affects the biological response to PM. We found little (PM_2.5_) or no effect (PM_2.5–10_, PNC) of PM fractions defined on the basis of particle size. PNC has been associated with cardiovascular events in time series studies, but not consistently [2]. A concern in observational studies is that PNC exposure measurement error is larger than for PM_2.5_. This however does not apply to our study as we measured exposure on-site.

We also observed no associations with either total or water-soluble trace metals, or EC. Contrary to the results of the respiratory analysis [15], we observed no positive associations with PNC and NO_2_ in the current analysis. Similarly to our respiratory analysis [15], and contrary to our hypothesis, OP did not display a strong and consistent association with acute vascular effects - in the two-pollutant models associations with OP were only fairly consistent with changes in hs-CRP. The absence of associations of the examined endpoints with OP may reflect the fact that the assay employed only examined the intrinsic potential of the particles to drive oxidation reactions in an acellular model: reflecting their content of redox-active transition metals and quinones. As PM can elicit oxidative stress through alternative pathways upon interaction with airway cells only a fraction of their in vivo activity can be accounted for by this assay. The OP values themselves also reflect, as with the transition metal concentrations, an aggregate (OP^TOTAL^) of the observed values in coarse (OP^PM2.5–10^), fine (OP^PM2.5^) and (quasi)ultrafine (OP^PM0.18^) fractions. On average, OP^PM2.5–10^ contributed in 30% to the OP^TOTAL^ (26% at the underground location and 31% at the outdoor locations), OP^PM2.5^ contributed 40% to the OP^TOTAL^ (30% underground, 44% outdoor sites), and OP^PM0.18^ contributed 31% to the OP^TOTAL^ (45% underground, 24% outdoor sites). There may be further merit therefore in exploring these parameters in the individual size fraction. In the current analysis we restricted ourselves to PM_10_, to limit the number of exposure variables under examination and thus the possibility of observing spurious interactions by chance.

The components consistently seen in the current analysis were OC, either in coarse or fine PM fraction, nitrate and sulfate, which were seen both in the “all sites” and the “outdoor-only sites” datasets. OC and other organic components of PM (e.g., polycyclic aromatic hydrocarbons, (semi)quinones) may be relevant for cardiovascular diseases [2], with two recent US studies reporting associations of organic carbon with cardiovascular hospital admissions [20] and mortality [21]. Sulfate and nitrate may not have direct effects - a review of the biological effects of secondary inorganic aerosols in controlled exposure studies showed the effects due to acidity of inorganic components rather than presence of single specific anions [22]. Since ambient PM in the Netherlands is less acidic due to neutralization by ambient ammonia [22], the sulfate and nitrate are unlikely to have a direct causal effect. However, a recent American Heart Association Scientific Statement did not completely exclude a direct role of particle sulfate in cardiovascular events [2] and a large epidemiological literature supports associations of sulfate with hospital admissions and mortality [23]–[26]. As the associations found for sulfate and nitrate with acute vascular biomarkers were robust against adjustment for a large series of co-pollutants, it remains unclear from our data what these associations represent. Sulfate and nitrate are likely indicators for other secondary aerosol components that may be biologically active.

### Timing of Effects

Different from our findings in the respiratory analysis [15], where the associations were the strongest immediately after and 2 h after exposure, in the current analysis the associations with acute vascular markers were mostly seen in the morning following the exposure. This difference seems reasonable as systemic inflammation would need more time to develop. The lack of associations is also consistent with the relatively long half-lives of CRP and especially fibrinogen. However, the literature regarding the timing of the effects is somewhat inconsistent. Some experimental studies in human volunteers reported increases in platelet count [27] and activation [28], thrombus formation [28], CRP and tPA [29] between 2 and 6 h after exposure to diesel exhaust or concentrated ambient ultrafine particles. However, no changes in CRP, fibrinogen, platelets or vWF were observed either 6 h after exposure of commuters to traffic-related air pollution [19], or immediately after experimental exposure to concentrated (quasi)ultrafine particles (PM_0.16_) [30]. When the more delayed effects were investigated, experimental diesel exhaust exposure led to a decrease in PAI-1 levels 22 h after exposure [31]; exposure to concentrated ambient particles was associated with elevated fibrinogen 24 h later [32]; and exposure of healthy volunteers to subway air pollution was associated with increase in fibrinogen 14 h later [33]. However, exposure of healthy volunteers to road-tunnel air pollution was not associated with changes in fibrinogen and PAI-1 14 h later [34]; and no changes in CRP, fibrinogen, platelets, vWF, tPA or PAI-1 were observed 18 h after exposure to concentrated PM_0.16_ [30].

It has to be noted that all of these studies used 1–2 h exposure periods which could potentially be too short to observe a marked increase in acute vascular biomarkers. A study on cardiovascular effects in healthy highway patrol officers by Riediker et al. showed that 9 h in-vehicle exposure to traffic-related PM_2.5_ was associated with increase in CRP and vWF 10-14 h after exposure [35]. When investigating the components of PM_2.5_, Cu and its compounds were associated with a decrease in PAI-1 [36]. However, none of the discussed studies measured the PM components with which we found robust associations; they also did not take a systematic two-pollutant approach.

Effect estimates for OC, nitrate and sulfate expressed per IQR were small (1–2% changes) and in this healthy adult population probably do not reflect an adverse effect. In patients with preexisting disease, changes in these markers might however elicit cardiovascular events. Our study therefore adds to the plausibility of observed associations of ambient air pollution with cardiovascular events.

### Strengths and Limitations

The use of a semi-experimental design in the current investigation allowed us to define two-pollutant models to investigate independent effects of a large number of individual PM characteristics. Since we performed air pollution characterization on site during exposure of volunteers, the exposure measurement error was largely due to instrumental errors and likely negligible compared to observational studies relying on central site monitoring. Instrumental precision of measurements was in general between 5–10% (Table S3), which is very low compared to the range of measured concentrations. This means that difference in instrumental precision was not a likely explanation for stronger associations with specific pollutants. In our design, we also reduced correlations between PM characteristics by performing repeated measurements at multiple locations with different source characteristics. Despite that, some correlations remained too high to interpret two-pollutant models and disentangle independent health effects of single PM characteristics. Since we specified a large number of models to investigate all possible combinations of air pollutants measured, we potentially faced a problem of chance finding in our results. That is why in our interpretation of the results we focused on the consistency of (significant) associations rather than individual significant associations present.

In conclusion, OC, nitrate and sulfate were most consistently associated with different biomarkers of acute cardiovascular risk. Associations with PM mass concentrations and OP were less consistent, whereas other measured components of the air pollution mixture, including PNC, EC, trace metals and NO_2_, were not associated with the biomarkers after adjusting for other pollutants.

## Supporting Information

Figure S1 **Timeline of a typical sampling day in the RAPTES project.**
(TIF)

Table S1 **Geometric means and minimum-maximum of 5-hour average air pollution concentrations.**
(DOC)

Table S2 **Spearman's correlation coefficients between PM characteristics.**
(DOC)

Table S3 **Precision and limits of detection (LOD) for 5-hour sampling periods.**
(DOC)

Table S4 **Adjusted associations between exposure to air pollution and percentage changes (post-pre) in hs-CRP.**
(DOC)

Table S5 **Adjusted associations between exposure to air pollution and percentage changes (post-pre) in fibrinogen.**
(DOC)

Table S6 **Adjusted associations between exposure to air pollution and percentage changes (post-pre) in tPA/PAI-1 complex.**
(DOC)

Table S7 **Adjusted associations between exposure to air pollution and percentage changes (post-pre) in platelet counts.**
(DOC)

Table S8 **Adjusted associations between exposure to air pollution and percentage changes (post-pre) in von Willebrand Factor.**
(DOC)

Table S9 **Two-pollutant models of associations between exposure to air pollution and percentage changes (25 h post-pre) in hs-CRP (all sites).**
(DOC)

Table S10 **Two-pollutant models of associations between exposure to air pollution and percentage changes (25 h post-pre) in fibrinogen (all sites).**
(DOC)

Table S11 **Two-pollutant models of associations between exposure to air pollution and percentage changes (25 h post-pre) in platelet counts (all sites).**
(DOC)

Table S12 **Two-pollutant models of associations between exposure to air pollution and percentage changes (25 h post-pre) in von Willebrand Factor (all sites).**
(DOC)

Table S13 **Two-pollutant models of associations between exposure to air pollution and percentage changes (25 h post-pre) in hs-CRP (outdoor sites).**
(DOC)

Table S14 **Two-pollutant models of associations between exposure to air pollution and percentage changes (25 h post-pre) in fibrinogen (outdoor sites).**
(DOC)

Table S15 **Two-pollutant models of associations between exposure to air pollution and percentage changes (25 h post-pre) in platelet counts (outdoor sites).**
(DOC)

Table S16 **Two-pollutant models of associations between exposure to air pollution and percentage changes (25 h post-pre) in von Willebrand Factor (outdoor sites).**
(DOC)

Table S17 **Two-pollutant models of associations between exposure to air pollution and percentage changes (25 h post-pre) in tPA/PAI-1 complex (all sites).**
(DOC)

Table S18 **Two-pollutant models of associations between exposure to air pollution and percentage changes (25 h post-pre) in tPA/PAI-1 complex (outdoor sites).**
(DOC)

Table S19 **Two-pollutant models of associations between exposure to air pollution and percentage changes (25 h post-pre) in tPA/PAI-1 complex (not log-transformed; all sites).**
(DOC)
