# Supplementary figures and images for: Composition of PM Affects Acute Vascular Inflammatory and Coagulative Markers - The RAPTES Project
Source: PLoS One. 2013 Mar 13;8(3):e58944. doi: 10.1371/journal.pone.0058944 (PMC3596332; doi:10.1371/journal.pone.0058944)

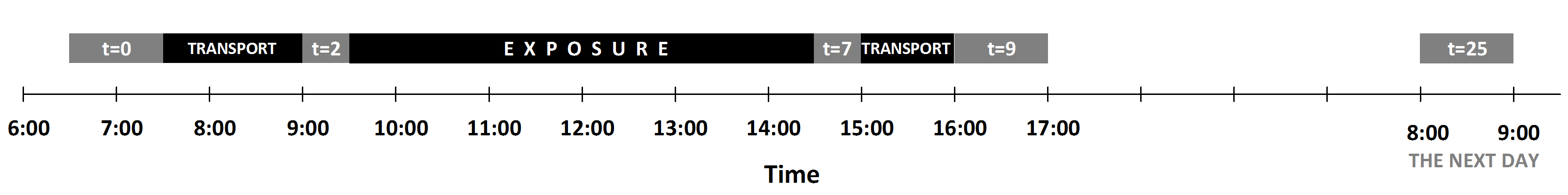

Supplement: Figure S1 — Timeline of a typical sampling day in the RAPTES project. (TIF) [file pone.0058944.s001.tif]
