# Supplementary material for: Composition of PM Affects Acute Vascular Inflammatory and Coagulative Markers - The RAPTES Project
Source: PLoS One. 2013 Mar 13;8(3):e58944. doi: 10.1371/journal.pone.0058944 (PMC3596332; doi:10.1371/journal.pone.0058944)
Supplement: Table S1 — Geometric means and minimum-maximum of 5-hour average air pollution concentrations. (DOC) [file pone.0058944.s002.doc]

**Table S1** Geometric means and minimum-maximum of 5-hour average air pollution concentrations.

|  | **All sites** | **Underground** | **Outdoor sites** | **Continuous traffic** | **Stop-and-go traffic** | **Farm** | **Urban background** |
| --- | --- | --- | --- | --- | --- | --- | --- |
| **PM_10_** | 76 (18-450) | 394 (354-450) | 37 (18-130) | 40 (36-44) | 34 (21-77) | 55 (30-130) | 26 (18-37) |
| **PM_2.5_** | 39 (8-167) | 140 (123-167) | 23 (8-95) | 23 (17-39) | 20 (13-63) | 36 (18-95) | 16 (8-30) |
| **PM_2.5-10_** | 32 (4-282) | 252 (212-282) | 13 (4-35) | 13 (4-22) | 13 (8-18) | 18 (12-35) | 9 (7-13) |
| **PNC** | 23.0 (7.0-74.7) | 29.4 (14.6-39.8) | 20.7 (7.0-74.7) | 66.5 (60.0-74.7) | 29.4 (12.8-42.6) | 9.6 (8.1-11.2) | 9.1 (7.0-11.8) |
| **Absorbance^a^** | 4 (0.3-16) | 14 (11-16) | 2 (0.3-8) | 6 (5-8) | 3 (1-6) | 1 (0.3-3) | 1 (1-2) |
| **EC (F)** | 4 (0.3-19) | 15 (12-19) | 2 (0.3-7) | 6 (6-7) | 3 (1-6) | 1 (0.3-2) | 1 (1-2) |
| **EC (C)** | 0.3 (0.0004-10) | 8 (6-10) | 0.07 (0.0004-0.5) | 0.4 (0.4-1) | 0.3 (0.2-0.5) | 0.02 (0.0004-0.2) | 0.01 (0.0004-0.3) |
| **OC (F)** | 2 (0.6-11) | 4 (2-11) | 1 (0.6-7) | 1 (1-4) | 1 (1-7) | 1 (1-3) | 1 (1-2) |
| **OC (C)** | 2 (0.5-6) | 4 (3-6) | 1 (0.5-5) | 1 (1-2) | 1 (1-2) | 3 (2-5) | 1 (0.5-1) |
| **Fe (tot)** | 3,699 (132-176,699) | 154,408 (133,299-176,699) | 690 (132-2,655) | 2,008 (1,287-2,655) | 884 (698-1,362) | 277 (132-513) | 365 (245-486) |
| **Fe (sol)** | 48 (7-431) | 114 (24-431) | 33 (7-75) | 61 (53-75) | 40 (24-71) | 15 (7-32) | 25 (16-43) |
| **Cu (tot)** | 160 (4-8,193) | 7,001 (5,267-8,193) | 29 (4-97) | 90 (75-97) | 35 (23-72) | 12 (4-35) | 16 (13-22) |
| **Cu (sol)** | 24 (2-1,637) | 517 (189-1,637) | 6 (2-18) | 15 (12-18) | 6 (3-13) | 5 (3-8) | 3 (2-7) |
| **Ni (tot)** | 9 (0.5-78) | 68 (59-78) | 4 (0.5-31) | 3 (2-4) | 4 (2-8) | 7 (0.5-31) | 3 (2-22) |
| **Ni (sol)** | 2 (0.6-10) | 2 (0.9-10) | 2 (0.6-5) | 2 (1-5) | 2 (1-4) | 1 (1-3) | 1 (1-3) |
| **V (tot)** | 6 (0.5-49) | 25 (18-49) | 3 (0.5-12) | 3 (2-6) | 4 (1-12) | 2 (0.5-5) | 3 (1-6) |
| **V (sol)** | 2 (0.1-10) | 0.9 (0.1-5) | 2 (0.3-10) | 2 (1-5) | 3 (1-10) | 1 (0.3-4) | 2 (1-4) |
| **Endotoxin** | 1 (0.3-44) | 0.7 (0.6-1) | 1 (0.3-44) | 0.4 (0.3-1) | 0.5 (0.3-1) | 17 (11-44) | 1 (0.3-1) |
| **NO_3_^- a^** | 4 (0.6-39) | 3 (0.6-9) | 5 (1-39) | 3 (1-8) | 4 (2-19) | 7 (1-39) | 5 (3-19) |
| **SO_4_^2- a^** | 3 (1-21) | 2 (1-5) | 3 (1-21) | 3 (2-6) | 3 (1-5) | 4 (2-10) | 4 (2-21) |
| **OP_AA_** | 103 (12-2,527) | 1,480 (996-2,527) | 27 (12-99) | 31 (24-36) | 31 (16-99) | 30 (14-97) | 20 (12-40) |
| **OP_GSH_** | 79 (4-2,505) | 1,580 (1,066-2,505) | 18 (4-45) | 30 (23-41) | 17 (14-19) | 24 (7-45) | 10 (4-29) |
| **OP_TOTAL_** | 190 (16-5,032) | 3,082 (2,368-5,032) | 47 (16-142) | 61 (48-76) | 50 (31-115) | 57 (21-142) | 31 (16-69) |
| **O_3_** | 7 (0.3-32) | 0.8 (0.3-6) | 18 (6-32) | 15 (6-24) | 16 (10-32) | 21 (13-26) | 22 (16-30) |
| **NO_2_** | 20 (9-34) | 20 (14-26) | 20 (9-34) | 23 (22-30) | 25 (15-34) | 17 (10-26) | 14 (9-18) |
| **NO_X_** | 36 (14-96) | 45 (15-69) | 32 (14-96) | 49 (43-60) | 47 (18-96) | 22 (14-34) | 19 (14-26) |

^a^ measured in PM_2.5_.

Absorbance is expressed in 10^-5^/m; endotoxin is presented in EU/m^3^; PM_10_, PM_2.5_, EC, OC, NO_3_^-^, SO_4_^2-^ are expressed in µg/m^3^; PNC in 10^3^/cm^3^; Fe, Cu, Ni and V in ng/m^3^; OP in 1/m^3^; O_3_, NO_2_ and NO_X_ in ppb. “Tot” is total, and “sol” is water-soluble metal extraction.
