# Supplementary material for: Composition of PM Affects Acute Vascular Inflammatory and Coagulative Markers - The RAPTES Project
Source: PLoS One. 2013 Mar 13;8(3):e58944. doi: 10.1371/journal.pone.0058944 (PMC3596332; doi:10.1371/journal.pone.0058944)
Supplement: Table S2 — Spearman's correlation coefficients between PM characteristics. (DOC) [file pone.0058944.s003.doc]

**Table S2** Spearman's correlation coefficients between PM characteristics.

|  | **PM_10_** | **PM_2.5_** | **PM_2.5-10_** | **PNC** | **Abs.^a^** | **EC (C)** | **EC (F)** | **OC (C)** | **OC (F)** | **Fe (tot)** | **Fe (sol)** | **Cu (tot)** | **Cu (sol)** | **Ni (tot)** | **Ni (sol)** | **V (tot)** | **V (sol)** | **End.** | **NO_3_^- a^** | **SO_4_^2- a^** | **OP^AA^** | **OP^GSH^** | **OP^TOTAL^** | **O_3_** | **NO_2_** | **NO_X_** |
| --- | --- | --- | --- | --- | --- | --- | --- | --- | --- | --- | --- | --- | --- | --- | --- | --- | --- | --- | --- | --- | --- | --- | --- | --- | --- | --- |
| **PM_10_** |  | 0.94 | 0.82 | 0.22 | 0.74 | 0.70 | 0.69 | 0.76 | 0.73 | 0.70 | 0.44 | 0.70 | 0.80 | 0.76 | 0.00 | 0.66 | -0.25 | 0.36 | 0.07 | -0.15 | 0.88 | 0.82 | 0.89 | -0.67 | 0.26 | 0.37 |
| **PM_2.5_** | *0.88* |  | 0.67 | 0.15 | 0.68 | 0.66 | 0.64 | 0.68 | 0.79 | 0.62 | 0.43 | 0.65 | 0.74 | 0.71 | -0.04 | 0.67 | -0.26 | 0.34 | 0.18 | -0.04 | 0.91 | 0.79 | 0.88 | -0.65 | 0.21 | 0.31 |
| **PM_2.5-10_** | *0.55* | *0.22* |  | 0.21 | 0.71 | 0.68 | 0.67 | 0.78 | 0.46 | 0.70 | 0.46 | 0.69 | 0.73 | 0.79 | 0.01 | 0.58 | -0.27 | 0.37 | -0.29 | -0.44 | 0.73 | 0.79 | 0.77 | -0.65 | 0.14 | 0.35 |
| **PNC** | *0.19* | *0.07* | *0.15* |  | 0.65 | 0.60 | 0.67 | 0.00 | -0.04 | 0.62 | 0.65 | 0.60 | 0.56 | 0.07 | 0.47 | 0.25 | 0.17 | -0.32 | -0.27 | -0.16 | 0.36 | 0.32 | 0.35 | -0.37 | 0.50 | 0.70 |
| **Absorbance^a^** | *0.37* | *0.22* | *0.31* | *0.84* |  | 0.88 | 0.98 | 0.48 | 0.49 | 0.92 | 0.75 | 0.89 | 0.92 | 0.64 | 0.19 | 0.66 | -0.19 | -0.01 | -0.30 | -0.36 | 0.80 | 0.73 | 0.78 | -0.81 | 0.39 | 0.70 |
| **EC (C)** | *0.28* | *0.17* | *0.26* | *0.77* | *0.73* |  | 0.89 | 0.45 | 0.36 | 0.88 | 0.75 | 0.92 | 0.93 | 0.54 | 0.25 | 0.77 | -0.06 | -0.07 | -0.24 | -0.41 | 0.79 | 0.77 | 0.80 | -0.71 | 0.27 | 0.61 |
| **EC (F)** | *0.25* | *0.13* | *0.19* | *0.86* | *0.96* | *0.77* |  | 0.42 | 0.43 | 0.92 | 0.74 | 0.89 | 0.90 | 0.60 | 0.24 | 0.71 | -0.15 | -0.02 | -0.35 | -0.38 | 0.79 | 0.73 | 0.76 | -0.81 | 0.36 | 0.67 |
| **OC (C)** | *0.52* | *0.39* | *0.57* | *-0.06* | *0.00* | *-0.04* | *-0.13* |  | 0.46 | 0.49 | 0.27 | 0.54 | 0.57 | 0.62 | -0.08 | 0.43 | -0.25 | 0.59 | -0.07 | -0.22 | 0.70 | 0.77 | 0.77 | -0.48 | -0.01 | 0.15 |
| **OC (F)** | *0.59* | *0.72* | *0.06* | *-0.20* | *0.05* | *-0.26* | *-0.07* | *0.08* |  | 0.37 | 0.20 | 0.37 | 0.53 | 0.62 | -0.18 | 0.37 | -0.35 | 0.22 | 0.36 | 0.08 | 0.66 | 0.48 | 0.60 | -0.50 | 0.19 | 0.21 |
| **Fe (tot)** | *0.24* | *0.04* | *0.27* | *0.90* | *0.83* | *0.77* | *0.81* | *0.07* | *-0.22* |  | 0.78 | 0.96 | 0.88 | 0.57 | 0.15 | 0.62 | -0.26 | -0.07 | -0.31 | -0.55 | 0.72 | 0.69 | 0.72 | -0.67 | 0.27 | 0.54 |
| **Fe (sol)** | *-0.05* | *-0.11* | *-0.01* | *0.86* | *0.65* | *0.59* | *0.66* | *-0.23* | *-0.27* | *0.80* |  | 0.79 | 0.74 | 0.40 | 0.31 | 0.48 | -0.07 | -0.15 | -0.38 | -0.44 | 0.51 | 0.43 | 0.45 | -0.51 | 0.08 | 0.56 |
| **Cu (tot)** | *0.28* | *0.12* | *0.26* | *0.82* | *0.76* | *0.82* | *0.77* | *0.12* | *-0.23* | *0.93* | *0.69* |  | 0.92 | 0.53 | 0.24 | 0.67 | -0.17 | -0.05 | -0.24 | -0.46 | 0.76 | 0.75 | 0.76 | -0.70 | 0.24 | 0.57 |
| **Cu (sol)** | *0.55* | *0.41* | *0.37* | *0.71* | *0.85* | *0.83* | *0.80* | *0.15* | *0.09* | *0.78* | *0.55* | *0.82* |  | 0.63 | 0.19 | 0.66 | -0.16 | 0.00 | -0.14 | -0.31 | 0.82 | 0.80 | 0.83 | -0.73 | 0.34 | 0.64 |
| **Ni (tot)** | *0.40* | *0.27* | *0.49* | *-0.09* | *0.11* | *-0.11* | *-0.01* | *0.22* | *0.37* | *-0.10* | *-0.11* | *-0.16* | *0.13* |  | 0.07 | 0.67 | -0.20 | 0.40 | -0.10 | -0.24 | 0.72 | 0.68 | 0.73 | -0.67 | 0.11 | 0.24 |
| **Ni (sol)** | *-0.01* | *-0.06* | *0.00* | *0.46* | *0.35* | *0.43* | *0.46* | *-0.22* | *-0.37* | *0.27* | *0.49* | *0.36* | *0.26* | *0.11* |  | 0.44 | 0.74 | 0.06 | 0.03 | 0.30 | 0.11 | 0.11 | 0.04 | -0.37 | 0.26 | 0.37 |
| **V (tot)** | *0.14* | *0.19* | *-0.05* | *0.20* | *0.19* | *0.47* | *0.29* | *-0.18* | *-0.18* | *0.04* | *0.06* | *0.21* | *0.22* | *0.16* | *0.75* |  | 0.20 | 0.27 | -0.11 | -0.16 | 0.79 | 0.70 | 0.76 | -0.80 | 0.16 | 0.35 |
| **V (sol)** | *0.04* | *0.07* | *0.00* | *0.19* | *0.14* | *0.42* | *0.24* | *-0.17* | *-0.30* | *0.00* | *0.11* | *0.13* | *0.13* | *0.16* | *0.81* | *0.96* |  | -0.07 | 0.15 | 0.51 | -0.15 | -0.22 | -0.20 | -0.01 | 0.23 | 0.21 |
| **Endotoxin** | *0.22* | *0.22* | *0.22* | *-0.37* | *-0.30* | *-0.49* | *-0.31* | *0.40* | *0.13* | *-0.52* | *-0.45* | *-0.49* | *-0.42* | *0.20* | *0.08* | *0.05* | *0.14* |  | -0.05 | 0.10 | 0.27 | 0.38 | 0.30 | -0.32 | -0.15 | -0.19 |
| **NO_3_^- a^** | *0.56* | *0.74* | *-0.10* | *-0.26* | *-0.12* | *-0.05* | *-0.21* | *0.11* | *0.64* | *-0.22* | *-0.27* | *-0.09* | *0.11* | *0.18* | *-0.13* | *0.20* | *0.06* | *0.02* |  | 0.67 | 0.03 | -0.19 | -0.05 | 0.17 | 0.27 | -0.17 |
| **SO_4_^2- a^** | *0.50* | *0.72* | *-0.12* | *-0.14* | *0.08* | *-0.07* | *0.05* | *0.12* | *0.54* | *-0.32* | *-0.29* | *-0.19* | *0.10* | *0.33* | *0.20* | *0.49* | *0.39* | *0.33* | *0.66* |  | -0.10 | -0.22 | -0.16 | 0.12 | 0.29 | -0.07 |
| **OP^AA^** | *0.75* | *0.79* | *0.32* | *0.28* | *0.51* | *0.47* | *0.45* | *0.40* | *0.38* | *0.26* | *0.03* | *0.35* | *0.56* | *0.24* | *0.07* | *0.47* | *0.36* | *0.00* | *0.57* | *0.67* |  | 0.82 | 0.95 | -0.78 | 0.32 | 0.47 |
| **OP^GSH^** | *0.54* | *0.48* | *0.44* | *0.12* | *0.27* | *0.39* | *0.28* | *0.50* | *-0.01* | *0.14* | *-0.15* | *0.35* | *0.47* | *0.15* | *0.02* | *0.21* | *0.15* | *0.13* | *0.10* | *0.34* | *0.54* |  | 0.92 | -0.69 | 0.14 | 0.35 |
| **OP^TOTAL^** | *0.73* | *0.73* | *0.40* | *0.22* | *0.42* | *0.50* | *0.35* | *0.56* | *0.24* | *0.23* | *-0.11* | *0.38* | *0.59* | *0.28* | *-0.07* | *0.37* | *0.26* | *-0.02* | *0.42* | *0.54* | *0.88* | *0.80* |  | -0.72 | 0.28 | 0.41 |
| **O_3_** | *-0.21* | *-0.15* | *-0.18* | *-0.35* | *-0.57* | *-0.33* | *-0.57* | *0.07* | *-0.06* | *-0.18* | *-0.14* | *-0.26* | *-0.35* | *-0.20* | *-0.54* | *-0.52* | *-0.48* | *-0.21* | *-0.03* | *-0.47* | *-0.46* | *-0.21* | *-0.27* |  | -0.36 | -0.57 |
| **NO_2_** | *0.49* | *0.45* | *0.28* | *0.56* | *0.74* | *0.60* | *0.67* | *0.06* | *0.26* | *0.52* | *0.34* | *0.52* | *0.71* | *0.28* | *0.28* | *0.36* | *0.27* | *-0.19* | *0.26* | *0.32* | *0.69* | *0.28* | *0.58* | *-0.62* |  | 0.65 |
| **NO_X_** | *0.32* | *0.21* | *0.25* | *0.75* | *0.87* | *0.71* | *0.87* | *-0.11* | *0.01* | *0.70* | *0.53* | *0.66* | *0.72* | *0.14* | *0.47* | *0.39* | *0.35* | *-0.23* | *-0.05* | *0.13* | *0.54* | *0.21* | *0.42* | *-0.68* | *0.91* |  |

^a^ measured in PM_2.5_.

“Tot” denotes total, while “sol” water-soluble metal extraction. Light-shaded italics represent correlations in the outdoor-only dataset.
