# Supplementary material for: Composition of PM Affects Acute Vascular Inflammatory and Coagulative Markers - The RAPTES Project
Source: PLoS One. 2013 Mar 13;8(3):e58944. doi: 10.1371/journal.pone.0058944 (PMC3596332; doi:10.1371/journal.pone.0058944)
Supplement: Table S3 — Precision and limits of detection (LOD) for 5-hour sampling periods. (DOC) [file pone.0058944.s004.doc]

**Table S3** Precision and limits of detection (LOD) for 5-hour sampling periods.

| **Component** | **Precision** | **LOD (% > LOD)** |
| --- | --- | --- |
| **PM_10_** | 4.0 | 9.77 (98%) |
| **PM_2.5_** | 4.6 | 9.77 (98%) |
| **Absorbance^a^** | 4.5 | 0.58 (95%) |
| **EC** | 8.4 | - |
| **OC** | 9.5 | - |
| **Fe (tot)** | 2.4 | 67.79 (100%) |
| **Fe (sol)** | 6.0 | 74.26 (79%-90%) |
| **Cu (tot)** | 15.8 | 2.07 (100%) |
| **Cu (sol)** | 5.2 | 3.03 (93%-97%) |
| **Ni (tot)** | 11.9 | 2.46 (93%-97%) |
| **Ni (sol)** | 8.9 | 0.20 (69%-97%) |
| **V (tot)** | 5.7 | 0.26 (100%) |
| **V (sol)** | 3.3 | 0.20 (97%-100%) |
| **NO_3_^- a^** | 0.4 | 0.33 (100%) |
| **SO_4_^2- a^** | 0.6 | 0.17 (100%) |
| **OP^AA^, OP^GSH^, OP^TOTAL^** | <5 | - |

^a^measured in PM_2.5_

PM_10_, PM_2.5_, NO_3_^-^ and SO_4_^2-^ in μg/m^3^, absorbance in 10^-5^/m, trace metals in ng/m^3^.

Precision in % coefficient of variation (CV).

Ranges in % > LOD for trace metals denote PM_2.5-10_ and PM_2.5_ fractions.
