# Supplementary material for: Composition of PM Affects Acute Vascular Inflammatory and Coagulative Markers - The RAPTES Project
Source: PLoS One. 2013 Mar 13;8(3):e58944. doi: 10.1371/journal.pone.0058944 (PMC3596332; doi:10.1371/journal.pone.0058944)
Supplement: Table S4 — Adjusted associations between exposure to air pollution and percentage changes (post-pre) in hs-CRP. (DOC) [file pone.0058944.s005.doc]

**Table S4** Adjusted associations between exposure to air pollution and percentage changes (post-pre) in hs-CRP.

|  | **IQR** | **All sites** | | | | **Outdoor sites** | | | |
| --- | --- | --- | --- | --- | --- | --- | --- | --- | --- |
|  |  | **2h post**-**exposure** | | **Next morning** | | **2h post**-**exposure** | | **Next morning** | |
|  |  | **Estimate (%)** | **95% CI (%)** | **Estimate (%)** | **95% CI (%)** | **Estimate (%)** | **95% CI (%)** | **Estimate (%)** | **95% CI (%)** |
| **PM_10_** | 13.50 | -0.13 | (-0.91 to 0.67) | 0.74* | (-0.01 to 1.50) | -1.26 | (-6.58 to 4.37) | -0.69 | (-5.24 to 4.08) |
| **PM_2.5_** | 11.54 | -0.25 | (-2.05 to 1.57) | 1.74* | (0.00 to 3.51) | -0.91 | (-6.66 to 5.20) | 0.36 | (-4.59 to 5.55) |
| **PM_2.5-10_** | 8.23 | -0.12 | (-0.87 to 0.63) | 0.67* | (-0.04 to 1.38) | -3.97 | (-14.18 to 7.45) | -6.36 | (-14.91 to 3.05) |
| **PNC** | 32,906 | -5.53 | (-15.80 to 5.99) | -4.31 | (-14.35 to 6.92) | -5.15 | (-16.98 to 8.37) | -8.04 | (-17.74 to 2.80) |
| **Absorbance^a^** | 3.49 | -1.73 | (-7.20 to 4.06) | 3.62 | (-1.92 to 9.48) | -5.87 | (-19.90 to 10.62) | -7.85 | (-19.51 to 5.50) |
| **EC (F)** | 4.35 | -1.91 | (-8.07 to 4.66) | 4.23 | (-2.05 to 10.91) | -6.34 | (-22.83 to 13.68) | -9.8 | (-23.35 to 6.16) |
| **EC (C)** | 0.40 | -0.07 | (-1.17 to 1.05) | 0.87 | (-0.16 to 1.91) | -5.29 | (-22.70 to 16.04) | -2.92 | (-18.00 to 14.93) |
| **OC (F)** | 1.82 | -0.16 | (-5.83 to 5.85) | 6.68** | (0.81 to 12.89) | -2.3 | (-12.41 to 8.97) | 0.53 | (-8.34 to 10.25) |
| **OC (C)** | 0.79 | -0.58 | (-4.65 to 3.66) | 1.64 | (-2.17 to 5.60) | -0.88 | (-7.77 to 6.53) | -0.71 | (-6.47 to 5.40) |
| **Fe (tot)** | 895.10 | -0.01 | (-0.14 to 0.11) | 0.11* | (-0.01 to 0.23) | -3.84 | (-14.43 to 8.06) | -6.03 | (-14.55 to 3.34) |
| **Fe (sol)** | 32.09 | 0.22 | (-3.64 to 4.25) | 2.92 | (-0.79 to 6.76) | -3.33 | (-18.24 to 14.30) | -5.62 | (-17.73 to 8.27) |
| **Cu (tot)** | 57.96 | 0 | (-0.18 to 0.17) | 0.16* | (0.00 to 0.32) | -4.11 | (-19.32 to 13.98) | -7.31 | (-19.53 to 6.75) |
| **Cu (sol)** | 8.65 | 0.04 | (-0.18 to 0.26) | 0.18* | (-0.02 to 0.39) | -4.92 | (-18.69 to 11.18) | -7.44 | (-18.53 to 5.15) |
| **Ni (tot)** | 3.53 | -0.15 | (-1.28 to 1.00) | 0.90* | (-0.16 to 1.98) | -0.48 | (-4.36 to 3.55) | -0.4 | (-3.66 to 2.98) |
| **Ni (sol)** | 1.82 | 1.2 | (-6.52 to 9.56) | 1.33 | (-5.96 to 9.18) | -0.03 | (-15.12 to 17.75) | -2.93 | (-15.15 to 11.04) |
| **V (tot)** | 2.04 | 0.01 | (-1.56 to 1.61) | 1.54** | (0.04 to 3.07) | 2.4 | (-6.03 to 11.58) | 7.95** ^b^ | (0.59 to 15.85) |
| **V (sol)** | 1.94 | 2.66 | (-5.31 to 11.30) | 7.90** ^b^ | (0.14 to 16.25) | 3.19 | (-7.01 to 14.51) | 7.70* ^b^ | (-1.12 to 17.30) |
| **Endotoxin** | 0.19 | 0.04 | (-0.12 to 0.19) | 0.01 | (-0.14 to 0.16) | 0.02 | (-0.16 to 0.21) | 0.05 | (-0.10 to 0.21) |
| **NO_3_^- a^** | 5.19 | 0.32 | (-4.90 to 5.83) | 1.89 | (-3.21 to 7.26) | -0.08 | (-6.14 to 6.37) | 1.25 | (-3.98 to 6.76) |
| **SO_4_^2- a^** | 2.99 | 1.65 | (-4.35 to 8.02) | 2.51 | (-3.59 to 9.00) | 1.64 | (-5.37 to 9.18) | 2.88 | (-3.46 to 9.63) |
| **OP^AA^** | 19.08 | -0.03 | (-0.30 to 0.25) | 0.30** | (0.07 to 0.53) | -1.77 | (-10.37 to 7.65) | 0.72 | (-5.70 to 7.58) |
| **OP^GSH^** | 15.53 | -0.02 | (-0.24 to 0.20) | 0.20** | (0.01 to 0.38) | -4.38 | (-17.87 to 11.32) | -5.75 | (-15.46 to 5.09) |
| **OP^TOTAL^** | 38.71 | -0.03 | (-0.30 to 0.25) | 0.28** | (0.05 to 0.52) | -3.65 | (-16.49 to 11.15) | -1.22 | (-10.87 to 9.47) |
| **O_3_** | 9.74 | 1.18 | (-6.24 to 9.19) | -7.76** | (-14.26 to -0.77) | -0.22 | (-18.06 to 21.51) | -2.67 | (-17.72 to 15.13) |
| **NO_2_** | 10.54 | -3.8 | (-15.63 to 9.69) | 10.81 | (-2.30 to 25.67) | -3.09 | (-17.71 to 14.14) | 3.78 | (-9.64 to 19.20) |
| **NO_X_** | 28.05 | -1.43 | (-11.53 to 9.83) | 6.02 | (-4.41 to 17.59) | -1.74 | (-14.55 to 13.00) | 1.25 | (-10.08 to 14.00) |

^a^ measured in PM_2.5_; ^b^ associations disappeared or decreased substantially after excluding 1% of influential observations. * p<0.10, ** p<0.05. “Tot” denotes total, while “sol” water-soluble metal extraction. Adjusted for the use of oral contraceptives and the use of oral contraceptives on the sampling day or the day before, temperature, relative humidity, and season. Estimates are percentage increases above population-average baseline expressed per outdoor-sites IQR.
