# Supplementary material for: Composition of PM Affects Acute Vascular Inflammatory and Coagulative Markers - The RAPTES Project
Source: PLoS One. 2013 Mar 13;8(3):e58944. doi: 10.1371/journal.pone.0058944 (PMC3596332; doi:10.1371/journal.pone.0058944)
Supplement: Table S5 — Adjusted associations between exposure to air pollution and percentage changes (post-pre) in fibrinogen. (DOC) [file pone.0058944.s006.doc]

**Table S5** Adjusted associations between exposure to air pollution and percentage changes (post-pre) in fibrinogen.

|  | **IQR** | **All sites** | | | | **Outdoor sites** | | | |
| --- | --- | --- | --- | --- | --- | --- | --- | --- | --- |
|  |  | **2h post**-**exposure** | | **Next morning** | | **2h post**-**exposure** | | **Next morning** | |
|  |  | **Estimate (%)** | **95% CI (%)** | **Estimate (%)** | **95% CI (%)** | **Estimate (%)** | **95% CI (%)** | **Estimate (%)** | **95% CI (%)** |
| **PM_10_** | 13.50 | 0.04 | (-0.10 to 0.17) | 0.1 | (-0.04 to 0.25) | 0.22 | (-0.60 to 1.05) | 0.57 | (-0.36 to 1.51) |
| **PM_2.5_** | 11.54 | 0.11 | (-0.20 to 0.42) | 0.27 | (-0.06 to 0.60) | 0.25 | (-0.62 to 1.13) | 0.75 | (-0.24 to 1.75) |
| **PM_2.5-10_** | 8.23 | 0.02 | (-0.10 to 0.15) | 0.09 | (-0.05 to 0.22) | 0.24 | (-1.46 to 1.97) | 0.08 | (-1.85 to 2.04) |
| **PNC** | 32,906 | -0.48 | (-2.37 to 1.45) | -0.92 | (-2.98 to 1.19) | -0.34 | (-2.27 to 1.62) | -0.94 | (-3.11 to 1.28) |
| **Absorbance^a^** | 3.49 | 0.09 | (-0.89 to 1.08) | 0.44 | (-0.61 to 1.50) | -0.4 | (-2.74 to 2.00) | -0.8 | (-3.42 to 1.90) |
| **EC (F)** | 4.35 | 0.02 | (-1.08 to 1.14) | 0.4 | (-0.78 to 1.59) | -0.51 | (-3.32 to 2.39) | -1.27 | (-4.41 to 1.98) |
| **EC (C)** | 0.40 | 0.06 | (-0.09 to 0.21) | 0.13 | (-0.04 to 0.30) | -0.28 | (-2.30 to 1.79) | -1.12 | (-3.81 to 1.66) |
| **OC (F)** | 1.82 | 0.55 | (-0.44 to 1.55) | 0.73 | (-0.36 to 1.82) | 0.24 | (-1.37 to 1.89) | 0.45 | (-1.37 to 2.31) |
| **OC (C)** | 0.79 | 0.18 | (-0.36 to 0.73) | 0.64* | (0.00 to 1.29) | 0.27 | (-0.45 to 0.99) | 0.52 | (-0.46 to 1.52) |
| **Fe (tot)** | 895.10 | 0 | (-0.01 to 0.02) | 0.01 | (-0.01 to 0.03) | -0.2 | (-1.35 to 0.97) | -0.84 | (-2.37 to 0.72) |
| **Fe (sol)** | 32.09 | 0.46* | (-0.06 to 0.98) | 0.49 | (-0.13 to 1.11) | -0.89 | (-2.54 to 0.79) | -1.22 | (-3.42 to 1.02) |
| **Cu (tot)** | 57.96 | 0.01 | (-0.01 to 0.03) | 0.02 | (-0.01 to 0.05) | -0.6 | (-2.30 to 1.13) | -1.17 | (-3.44 to 1.15) |
| **Cu (sol)** | 8.65 | 0.02 | (-0.01 to 0.05) | 0.03* | (-0.01 to 0.06) | -0.2 | (-1.76 to 1.39) | -1.13 | (-3.18 to 0.95) |
| **Ni (tot)** | 3.53 | 0.05 | (-0.10 to 0.20) | 0.13 | (-0.05 to 0.31) | 0.19 | (-0.20 to 0.58) | 0.13 | (-0.42 to 0.67) |
| **Ni (sol)** | 1.82 | 0.41 | (-0.63 to 1.47) | 0.28 | (-0.98 to 1.55) | -0.33 | (-1.95 to 1.31) | -0.71 | (-2.89 to 1.51) |
| **V (tot)** | 2.04 | 0.06 | (-0.16 to 0.27) | 0.2 | (-0.06 to 0.45) | 0.34 | (-0.55 to 1.23) | 1.13* ^b^ | (-0.06 to 2.34) |
| **V (sol)** | 1.94 | 0.16 | (-0.90 to 1.22) | 0.54 | (-0.73 to 1.83) | 0.32 | (-0.75 to 1.39) | 0.82 | (-0.62 to 2.27) |
| **Endotoxin** | 0.19 | 0 | (-0.02 to 0.03) | 0 | (-0.03 to 0.03) | 0.01 | (-0.02 to 0.04) | 0 | (-0.03 to 0.03) |
| **NO_3_^- a^** | 5.19 | 0.39 | (-0.50 to 1.28) | 0.98** | (0.01 to 1.96) | 0.31 | (-0.61 to 1.23) | 1.00* | (-0.04 to 2.05) |
| **SO_4_^2- a^** | 2.99 | 0.36 | (-0.65 to 1.38) | 1.33** ^b^ | (0.11 to 2.57) | 0.34 | (-0.71 to 1.40) | 1.50** | (0.19 to 2.83) |
| **OP^AA^** | 19.08 | 0.02 | (-0.03 to 0.06) | 0.03 | (-0.01 to 0.08) | 0.39 | (-0.94 to 1.74) | 0.66 | (-0.79 to 2.13) |
| **OP^GSH^** | 15.53 | 0.01 | (-0.02 to 0.05) | 0.03 | (-0.01 to 0.07) | 0.19 | (-2.00 to 2.43) | -0.06 | (-2.45 to 2.38) |
| **OP^TOTAL^** | 38.71 | 0.02 | (-0.03 to 0.06) | 0.04 | (-0.01 to 0.08) | 0.54 | (-1.53 to 2.66) | 0.77 | (-1.49 to 3.08) |
| **O_3_** | 9.74 | -0.02 | (-1.32 to 1.30) | -0.88 | (-2.25 to 0.52) | 0.21 | (-2.71 to 3.23) | -1.58 | (-4.82 to 1.77) |
| **NO_2_** | 10.54 | -0.37 | (-2.56 to 1.88) | 0.29 | (-2.09 to 2.73) | -0.11 | (-2.52 to 2.37) | 0.49 | (-2.24 to 3.30) |
| **NO_X_** | 28.05 | 0.05 | (-1.75 to 1.90) | 0.1 | (-1.85 to 2.09) | -0.31 | (-2.38 to 1.80) | -0.4 | (-2.72 to 1.98) |

For explanation see Table S4.
