# Supplementary material for: Composition of PM Affects Acute Vascular Inflammatory and Coagulative Markers - The RAPTES Project
Source: PLoS One. 2013 Mar 13;8(3):e58944. doi: 10.1371/journal.pone.0058944 (PMC3596332; doi:10.1371/journal.pone.0058944)
Supplement: Table S6 — Adjusted associations between exposure to air pollution and percentage changes (post-pre) in tPA/PAI-1 complex. (DOC) [file pone.0058944.s007.doc]

**Table S6** Adjusted associations between exposure to air pollution and percentage changes (post-pre) in tPA/PAI-1 complex.

|  | **IQR** | **All sites** | | | | **Outdoor sites** | | | |
| --- | --- | --- | --- | --- | --- | --- | --- | --- | --- |
|  |  | **2h post**-**exposure** | | **Next morning** | | **2h post**-**exposure** | | **Next morning** | |
|  |  | **Estimate (%)** | **95% CI (%)** | **Estimate (%)** | **95% CI (%)** | **Estimate (%)** | **95% CI (%)** | **Estimate (%)** | **95% CI (%)** |
| **PM_10_** | 13.50 | 0.66 | (-0.77 to 2.11) | 1.09* | (-0.09 to 2.28) | 3.71 | (-5.53 to 13.85) | 2.06 | (-5.56 to 10.30) |
| **PM_2.5_** | 11.54 | 1.25 | (-2.03 to 4.64) | 2.36* | (-0.37 to 5.16) | 4.34 | (-5.63 to 15.37) | 0.34 | (-7.75 to 9.12) |
| **PM_2.5-10_** | 8.23 | 0.67 | (-0.68 to 2.04) | 1.03* | (-0.08 to 2.16) | 3.57 | (-14.45 to 25.39) | 12.82 | (-3.51 to 31.91) |
| **PNC** | 32,906 | -1.57 | (-19.92 to 20.99) | 14.9 | (-3.15 to 36.32) | -5.52 | (-24.27 to 17.87) | 10.11 | (-8.33 to 32.26) |
| **Absorbance^a^** | 3.49 | 2.58 | (-7.55 to 13.83) | 9.73** | (0.81 to 19.45) | -3.31 | (-26.16 to 26.62) | 16.46 ^b^ | (-6.64 to 45.26) |
| **EC (F)** | 4.35 | 1.84 | (-9.45 to 14.55) | 9.82* | (-0.28 to 20.93) | -8.09 | (-33.49 to 27.02) | 13.94 ^b^ | (-12.75 to 48.80) |
| **EC (C)** | 0.40 | 0.41 | (-1.53 to 2.39) | 1.47* | (-0.17 to 3.13) | 4.78 | (-24.48 to 45.38) | 18.43 ^b^ | (-10.34 to 56.43) |
| **OC (F)** | 1.82 | 5.7 | (-5.03 to 17.63) | 5.33 | (-3.79 to 15.32) | 1.44 | (-16.16 to 22.74) | -0.93 | (-15.41 to 16.04) |
| **OC (C)** | 0.79 | 5.84 | (-1.55 to 13.78) | 4.93 | (-1.23 to 11.48) | 7.41 | (-4.26 to 20.51) | 4.33 | (-5.52 to 15.21) |
| **Fe (tot)** | 895.10 | 0.07 | (-0.15 to 0.29) | 0.16* | (-0.03 to 0.35) | -5.41 | (-21.43 to 13.87) | 7.53 | (-8.18 to 25.91) |
| **Fe (sol)** | 32.09 | -2.88 | (-9.31 to 4.00) | 0.87 | (-4.82 to 6.91) | -15.71 | (-35.60 to 10.32) | -1.96 | (-22.16 to 23.48) |
| **Cu (tot)** | 57.96 | 0.06 | (-0.25 to 0.36) | 0.21 | (-0.05 to 0.47) | -6.94 | (-29.42 to 22.72) | 6.58 | (-15.81 to 34.94) |
| **Cu (sol)** | 8.65 | 0.1 | (-0.28 to 0.49) | 0.22 | (-0.10 to 0.54) | -5.16 | (-26.20 to 21.88) | 4.17 | (-15.79 to 28.86) |
| **Ni (tot)** | 3.53 | 0.38 | (-1.60 to 2.41) | 1.25 | (-0.45 to 2.98) | -1.48 | (-7.49 to 4.92) | -0.93 | (-6.29 to 4.75) |
| **Ni (sol)** | 1.82 | 0.76 | (-12.28 to 15.74) | -5.21 | (-16.53 to 7.64) | -17.48 | (-36.24 to 6.80) | -21.29** ^b^ | (-36.73 to -2.08) |
| **V (tot)** | 2.04 | 1.59 | (-1.21 to 4.47) | 1.76 | (-0.73 to 4.32) | 3.53 | (-10.02 to 19.11) | -4.4 | (-15.16 to 7.72) |
| **V (sol)** | 1.94 | 8.13 | (-5.97 to 24.35) | -4.94 | (-15.74 to 7.24) | 1.23 | (-14.52 to 19.87) | -8.83 | (-20.95 to 5.16) |
| **Endotoxin** | 0.19 | 0.07 | (-0.20 to 0.36) | -0.08 | (-0.31 to 0.15) | 0.12 | (-0.18 to 0.42) | -0.03 | (-0.28 to 0.22) |
| **NO_3_^- a^** | 5.19 | 5.74 | (-3.95 to 16.40) | 1.9 | (-5.98 to 10.43) | 5.73 | (-4.81 to 17.44) | 1.28 | (-7.24 to 10.58) |
| **SO_4_^2- a^** | 2.99 | 4.24 | (-6.56 to 16.28) | 3.75 | (-5.72 to 14.17) | 4.56 | (-7.28 to 17.91) | 4.64 | (-5.75 to 16.18) |
| **OP^AA^** | 19.08 | 0.08 | (-0.40 to 0.56) | 0.2 | (-0.19 to 0.60) | 10.33 | (-5.47 to 28.76) | 4.77 | (-7.65 to 18.87) |
| **OP^GSH^** | 15.53 | 0.18 | (-0.20 to 0.56) | 0.2 | (-0.11 to 0.51) | 13.87 | (-11.17 to 45.96) | 14.27 | (-6.51 to 39.68) |
| **OP^TOTAL^** | 38.71 | 0.16 | (-0.33 to 0.65) | 0.24 | (-0.16 to 0.64) | 17.75 | (-7.20 to 49.40) | 10.93 | (-8.62 to 34.66) |
| **O_3_** | 9.74 | -9.98 | (-21.61 to 3.38) | -15.08** | (-24.16 to -4.92) | -13.71 | (-38.18 to 20.43) | -25.12** | (-42.87 to -1.85) |
| **NO_2_** | 10.54 | 13.98 | (-10.10 to 44.51) | 20.50* | (-0.80 to 46.36) | 7.5 | (-18.46 to 41.71) | 12.83 | (-10.08 to 41.58) |
| **NO_X_** | 28.05 | 2.33 | (-15.79 to 24.35) | 16.76* | (-0.34 to 36.80) | 3.12 | (-18.54 to 30.55) | 10.25 | (-9.14 to 33.79) |

For explanation see Table S4.
