# Supplementary material for: Composition of PM Affects Acute Vascular Inflammatory and Coagulative Markers - The RAPTES Project
Source: PLoS One. 2013 Mar 13;8(3):e58944. doi: 10.1371/journal.pone.0058944 (PMC3596332; doi:10.1371/journal.pone.0058944)
Supplement: Table S7 — Adjusted associations between exposure to air pollution and percentage changes (post-pre) in platelet counts. (DOC) [file pone.0058944.s008.doc]

**Table S7** Adjusted associations between exposure to air pollution and percentage changes (post-pre) in platelet counts.

|  | **IQR** | **All sites** | | | | **Outdoor sites** | | | |
| --- | --- | --- | --- | --- | --- | --- | --- | --- | --- |
|  |  | **2h post**-**exposure** | | **Next morning** | | **2h post**-**exposure** | | **Next morning** | |
|  |  | **Estimate (%)** | **95% CI (%)** | **Estimate (%)** | **95% CI (%)** | **Estimate (%)** | **95% CI (%)** | **Estimate (%)** | **95% CI (%)** |
| **PM_10_** | 13.50 | 0.05 | (-0.04 to 0.13) | 0.04 | (-0.07 to 0.14) | 0.08 | (-0.46 to 0.63) | 0.68** | (0.05 to 1.32) |
| **PM_2.5_** | 11.54 | 0.1 | (-0.10 to 0.30) | 0.07 | (-0.17 to 0.32) | 0.08 | (-0.50 to 0.66) | 0.72** | (0.04 to 1.41) |
| **PM_2.5-10_** | 8.23 | 0.04 | (-0.04 to 0.13) | 0.04 | (-0.06 to 0.14) | 0.14 | (-1.00 to 1.30) | 1.06 | (-0.23 to 2.35) |
| **PNC** | 32,906 | 0.28 | (-0.98 to 1.57) | -1.15 | (-2.69 to 0.40) | 0.12 | (-1.18 to 1.45) | -0.96 | (-2.48 to 0.57) |
| **Absorbance^a^** | 3.49 | 0.28 | (-0.36 to 0.92) | -0.05 | (-0.82 to 0.73) | -0.24 | (-1.82 to 1.38) | -1.13 | (-2.94 to 0.72) |
| **EC (F)** | 4.35 | 0.34 | (-0.38 to 1.06) | -0.08 | (-0.95 to 0.80) | -0.04 | (-1.95 to 1.90) | -1.23 | (-3.40 to 0.99) |
| **EC (C)** | 0.40 | 0.04 | (-0.08 to 0.16) | 0.01 | (-0.14 to 0.15) | 0.15 | (-1.79 to 2.11) | -0.99 | (-3.18 to 1.25) |
| **OC (F)** | 1.82 | -0.04 | (-0.69 to 0.61) | -0.12 | (-0.91 to 0.69) | -0.03 | (-1.09 to 1.05) | 0.1 | (-1.15 to 1.37) |
| **OC (C)** | 0.79 | 0.24 | (-0.21 to 0.69) | 0.79** | (0.25 to 1.33) | 0.18 | (-0.50 to 0.87) | 1.01** | (0.22 to 1.81) |
| **Fe (tot)** | 895.10 | 0 | (-0.01 to 0.02) | 0 | (-0.01 to 0.02) | 0.73 | (-0.38 to 1.85) | -0.2 | (-1.49 to 1.10) |
| **Fe (sol)** | 32.09 | 0.02 | (-0.41 to 0.44) | -0.1 | (-0.62 to 0.42) | 0.96 | (-0.62 to 2.57) | -0.93 | (-2.72 to 0.90) |
| **Cu (tot)** | 57.96 | 0 | (-0.02 to 0.02) | 0 | (-0.02 to 0.03) | 1.1 | (-0.54 to 2.76) | 0.04 | (-1.85 to 1.97) |
| **Cu (sol)** | 8.65 | 0 | (-0.02 to 0.02) | 0 | (-0.03 to 0.03) | 0.98 | (-0.50 to 2.49) | 0.29 | (-1.42 to 2.03) |
| **Ni (tot)** | 3.53 | 0.02 | (-0.10 to 0.14) | 0.01 | (-0.15 to 0.16) | -0.08 | (-0.44 to 0.29) | -0.02 | (-0.48 to 0.44) |
| **Ni (sol)** | 1.82 | -0.68 | (-1.52 to 0.17) | 0.64 | (-0.41 to 1.70) | -0.13 | (-1.67 to 1.43) | -0.62 | (-2.40 to 1.20) |
| **V (tot)** | 2.04 | -0.05 | (-0.22 to 0.13) | 0.06 | (-0.15 to 0.27) | -0.5 | (-1.33 to 0.34) | -0.4 | (-1.33 to 0.54) |
| **V (sol)** | 1.94 | -0.86** | (-1.70 to -0.01) | -0.07 | (-1.15 to 1.01) | -0.61 | (-1.60 to 0.39) | -0.39 | (-1.52 to 0.76) |
| **Endotoxin** | 0.19 | -0.01 | (-0.02 to 0.01) | 0.01 | (-0.02 to 0.03) | -0.01 | (-0.02 to 0.01) | 0.01 | (-0.01 to 0.03) |
| **NO_3_^- a^** | 5.19 | 0.17 | (-0.41 to 0.75) | 0.74** | (0.02 to 1.47) | 0.13 | (-0.48 to 0.74) | 0.83** | (0.12 to 1.55) |
| **SO_4_^2- a^** | 2.99 | 0.22 | (-0.45 to 0.89) | 0.29 | (-0.58 to 1.17) | 0.22 | (-0.48 to 0.92) | 0.38 | (-0.49 to 1.25) |
| **OP^AA^** | 19.08 | 0.01 | (-0.02 to 0.04) | 0.01 | (-0.03 to 0.04) | -0.16 | (-1.01 to 0.71) | 0.26 | (-0.67 to 1.21) |
| **OP^GSH^** | 15.53 | 0.01 | (-0.01 to 0.03) | 0.02 | (-0.01 to 0.05) | -0.56 | (-1.95 to 0.84) | 0.09 | (-1.46 to 1.66) |
| **OP^TOTAL^** | 38.71 | 0.01 | (-0.02 to 0.04) | 0.02 | (-0.02 to 0.05) | -0.39 | (-1.71 to 0.95) | 0.35 | (-1.11 to 1.82) |
| **O_3_** | 9.74 | -0.05 | (-0.90 to 0.80) | 0.21 | (-0.82 to 1.25) | 1.58 | (-0.38 to 3.59) | 1.2 | (-1.06 to 3.51) |
| **NO_2_** | 10.54 | -1.06 | (-2.49 to 0.39) | -1.69* | (-3.40 to 0.06) | -1.60* | (-3.16 to -0.02) | -1.43 | (-3.25 to 0.41) |
| **NO_X_** | 28.05 | -0.64 | (-1.82 to 0.55) | -1.40* | (-2.81 to 0.04) | -1.35* | (-2.69 to 0.01) | -1.46* | (-3.01 to 0.11) |

For explanation see Table S4.
