# Supplementary material for: Composition of PM Affects Acute Vascular Inflammatory and Coagulative Markers - The RAPTES Project
Source: PLoS One. 2013 Mar 13;8(3):e58944. doi: 10.1371/journal.pone.0058944 (PMC3596332; doi:10.1371/journal.pone.0058944)
Supplement: Table S8 — Adjusted associations between exposure to air pollution and percentage changes (post-pre) in von Willebrand Factor. (DOC) [file pone.0058944.s009.doc]

**Table S8** Adjusted associations between exposure to air pollution and percentage changes (post-pre) in von Willebrand Factor.

|  | **IQR** | **All sites** | | | | **Outdoor sites** | | | |
| --- | --- | --- | --- | --- | --- | --- | --- | --- | --- |
|  |  | **2h post**-**exposure** | | **Next morning** | | **2h post**-**exposure** | | **Next morning** | |
|  |  | **Estimate (%)** | **95% CI (%)** | **Estimate (%)** | **95% CI (%)** | **Estimate (%)** | **95% CI (%)** | **Estimate (%)** | **95% CI (%)** |
| **PM_10_** | 13.50 | -0.01 | (-0.19 to 0.16) | 0.22** | (0.02 to 0.41) | 0.8 | (-0.19 to 1.80) | 0.84 | (-0.29 to 1.99) |
| **PM_2.5_** | 11.54 | 0.08 | (-0.32 to 0.49) | 0.58** | (0.12 to 1.03) | 1.14** | (0.08 to 2.20) | 1.28** | (0.07 to 2.51) |
| **PM_2.5-10_** | 8.23 | -0.04 | (-0.20 to 0.13) | 0.18* | (-0.01 to 0.37) | -0.32 | (-2.38 to 1.79) | -0.31 | (-2.64 to 2.07) |
| **PNC** | 32,906 | 0.5 | (-2.11 to 3.18) | -0.04 | (-2.80 to 2.80) | -0.1 | (-2.46 to 2.32) | 0.61 | (-1.96 to 3.26) |
| **Absorbance^a^** | 3.49 | 0.17 | (-1.12 to 1.46) | 0.49 | (-0.91 to 1.91) | 1.09 | (-1.81 to 4.07) | -0.14 | (-3.35 to 3.18) |
| **EC (F)** | 4.35 | -0.16 | (-1.60 to 1.31) | 0.32 | (-1.27 to 1.94) | 0.49 | (-2.97 to 4.07) | 1.1 | (-2.64 to 4.99) |
| **EC (C)** | 0.40 | -0.01 | (-0.24 to 0.23) | 0.25* | (-0.02 to 0.52) | -0.63 | (-3.88 to 2.74) | 0.17 | (-3.60 to 4.10) |
| **OC (F)** | 1.82 | 1.33* | (-0.02 to 2.70) | 1.81** | (0.26 to 3.38) | 2.91** | (0.96 to 4.89) | 3.10** | (0.82 to 5.44) |
| **OC (C)** | 0.79 | -0.38 | (-1.27 to 0.50) | 0.48 | (-0.53 to 1.50) | 0.07 | (-1.11 to 1.26) | 0.3 | (-1.12 to 1.74) |
| **Fe (tot)** | 895.10 | -0.01 | (-0.03 to 0.02) | 0.02 | (-0.01 to 0.06) | 0.5 | (-1.34 to 2.37) | 0.66 | (-1.52 to 2.88) |
| **Fe (sol)** | 32.09 | 0.25 | (-0.60 to 1.10) | 0.43 | (-0.51 to 1.38) | -1.21 | (-3.88 to 1.55) | -0.58 | (-3.78 to 2.73) |
| **Cu (tot)** | 57.96 | 0 | (-0.04 to 0.04) | 0.03 | (-0.01 to 0.07) | -0.44 | (-3.20 to 2.40) | 1.17 | (-2.04 to 4.48) |
| **Cu (sol)** | 8.65 | 0.01 | (-0.04 to 0.05) | 0.03 | (-0.02 to 0.08) | 1.34 | (-1.21 to 3.96) | 0.9 | (-2.14 to 4.02) |
| **Ni (tot)** | 3.53 | -0.08 | (-0.31 to 0.16) | 0.19 | (-0.09 to 0.48) | 0.06 | (-0.58 to 0.69) | 0.07 | (-0.72 to 0.86) |
| **Ni (sol)** | 1.82 | 0.03 | (-1.67 to 1.75) | -0.05 | (-1.92 to 1.86) | -1.56 | (-4.12 to 1.08) | -2 | (-5.12 to 1.21) |
| **V (tot)** | 2.04 | -0.1 | (-0.44 to 0.24) | 0.04 | (-0.35 to 0.43) | -0.89 | (-2.32 to 0.56) | -1.3 | (-3.06 to 0.48) |
| **V (sol)** | 1.94 | -0.43 | (-2.16 to 1.32) | -0.42 | (-2.31 to 1.51) | -1.25 | (-2.97 to 0.49) | -1.7 | (-3.77 to 0.41) |
| **Endotoxin** | 0.19 | 0.01 | (-0.03 to 0.04) | 0.01 | (-0.03 to 0.05) | 0.02 | (-0.01 to 0.05) | 0.02 | (-0.02 to 0.05) |
| **NO_3_^- a^** | 5.19 | 0.87 | (-0.33 to 2.08) | 0.66 | (-0.63 to 1.97) | 0.99* | (-0.12 to 2.11) | 1.01 | (-0.27 to 2.31) |
| **SO_4_^2- a^** | 2.99 | 0.71 | (-0.66 to 2.10) | 0.93 | (-0.91 to 2.80) | 0.36 | (-0.92 to 1.65) | 0.84 | (-0.88 to 2.59) |
| **OP^AA^** | 19.08 | 0 | (-0.06 to 0.05) | 0.05 | (-0.01 to 0.12) | 1.08 | (-0.37 to 2.56) | 1.28 | (-0.49 to 3.09) |
| **OP^GSH^** | 15.53 | -0.01 | (-0.05 to 0.04) | 0.05* | (0.00 to 0.10) | 0.91 | (-1.44 to 3.32) | 0.68 | (-2.03 to 3.47) |
| **OP^TOTAL^** | 38.71 | -0.01 | (-0.06 to 0.05) | 0.06* | (-0.01 to 0.12) | 1.26 | (-1.00 to 3.57) | 1.56 | (-1.18 to 4.38) |
| **O_3_** | 9.74 | -0.33 | (-2.04 to 1.41) | -0.61 | (-2.48 to 1.29) | 0.02 | (-3.54 to 3.71) | 0.3 | (-3.65 to 4.42) |
| **NO_2_** | 10.54 | 2.61* | (-0.43 to 5.74) | 1.19 | (-2.00 to 4.49) | 1.6 | (-1.36 to 4.66) | 1.73 | (-1.60 to 5.18) |
| **NO_X_** | 28.05 | 0.75 | (-1.71 to 3.27) | 0.45 | (-2.13 to 3.11) | 0.51 | (-2.03 to 3.11) | -0.2 | (-2.99 to 2.67) |

For explanation see Table S4.
