# Supplementary material for: Composition of PM Affects Acute Vascular Inflammatory and Coagulative Markers - The RAPTES Project
Source: PLoS One. 2013 Mar 13;8(3):e58944. doi: 10.1371/journal.pone.0058944 (PMC3596332; doi:10.1371/journal.pone.0058944)
Supplement: Table S9 — Two-pollutant models of associations between exposure to air pollution and percentage changes (25 h post-pre) in hs-CRP (all sites). (DOC) [file pone.0058944.s010.doc]

**Table S9** Two-pollutant models of associations between exposure to air pollution and percentage changes (25h post-pre) in hs-CRP (all sites).

|  | **IQR** | **A D J U S T M E N T P O L L U T A N T S** | | | | | | | | | | | | | | | | | | | | | | | | | |
| --- | --- | --- | --- | --- | --- | --- | --- | --- | --- | --- | --- | --- | --- | --- | --- | --- | --- | --- | --- | --- | --- | --- | --- | --- | --- | --- | --- |
|  |  | **PM_10_** | **PM_2.5_** | **PM_2.5_**-**_10_** | **PNC** | **Abs.^a^** | **EC (F)** | **EC (C)** | **OC (F)** | **OC (C)** | **Fe (tot)** | **Fe (sol)** | **Cu (tot)** | **Cu (sol)** | **Ni (tot)** | **Ni (sol)** | **V (tot)** | **V (sol)** | **End.** | **NO_3_^- a^** | **SO_4_^2- a^** | **OP^AA^** | **OP^GSH^** | **OP^TOTAL^** | **O_3_** | **NO_2_** | **NO_X_** |
| **PM_10_** | 13.50 | 0.74* | 0.19 | 1.74 | 0.79** | 1.66* | 1.46* | 1.04 | 0.36 | 1.01* | -0.42 | 0.51 | -0.50 | 0.41 | 0.70 | 0.69* | 0.13 | 0.64* | 0.79** | 0.72* | 0.77** | -0.71 | 0.05 | -0.71 | 0.05 | 0.63 | 0.68 |
| **PM_2.5_** | 11.54 | 1.32 | 1.74* | 1.48 | 1.81** | 2.91* | 2.54 | 1.91 | 0.76 | 2.46* | 0.76 | 1.27 | 0.29 | 1.07 | 1.53 | 1.62* | 0.66 | 1.65* | 1.80** | 1.71* | 1.74* | -0.46 | 1.06 | 0.13 | 0.44 | 1.45 | 1.61 |
| **PM_2.5_**-**_10_** | 8.23 | -0.93 | 0.11 | 0.67* | 0.73** | 1.44 | 1.32 | 0.53 | 0.33 | 0.83 | -1.98 | 0.44 | -0.84 | 0.33 | 0.42 | 0.63* | -0.03 | 0.55 | 0.72* | 0.66* | 0.72* | -0.80 | -0.55 | -1.11 | -0.06 | 0.58 | 0.61 |
| **PNC** | 32,906 | -5.75 | -5.23 | -5.97 | -4.31 | -9.92 | -9.91 | -4.47 | -4.89 | -2.23 | -4.29 | -5.16 | -4.24 | -3.83 | -2.84 | -3.08 | -3.99 | -2.87 | -4.66 | -3.46 | -3.49 | -8.94 | -8.85 | -8.95 | -8.87 | -11.23* | -11.46* |
| **Absorbance ^a^** | 3.49 | -7.02 | -4.11 | -6.25 | 6.31* | 3.62 | -3.39 | -3.02 | 0.46 | 3.53 | -6.61 | 1.95 | -6.08 | 1.05 | -0.37 | 3.76 | -1.60 | 3.73 | 4.41 | 3.71 | 4.15 | -9.03* | -6.62 | -9.52* | -8.55 | 2.02 | 2.71 |
| **EC (F)** | 4.35 | -6.30 | -3.30 | -6.02 | 7.24* | 8.33 | 4.23 | -2.79 | 1.30 | 3.82 | -8.03 | 2.26 | -6.79 | 1.44 | -0.65 | 4.19 | -2.23 | 3.85 | 5.13 | 4.53 | 4.98 | -9.10* | -5.72 | -8.79 | -6.75 | 2.63 | 3.25 |
| **EC (C)** | 0.40 | -0.52 | -0.18 | 0.12 | 0.96* | 1.41 | 1.31 | 0.87 | 0.22 | 1.00 | -1.37 | 0.60 | -2.64 | 0.36 | 0.43 | 0.90 | 0.02 | 0.82 | 0.85 | 0.86 | 0.92* | -1.82 | -0.63 | -2.15 | -0.38 | 0.69 | 0.69 |
| **OC (F)** | 1.82 | 5.00 | 4.95 | 5.18 | 6.81** | 6.42* | 6.13* | 6.05 | 6.68** | 6.94** | 5.38 | 6.12* | 5.17 | 6.28 | 5.86* | 6.81** | 5.29* | 8.57** | 6.92** | 6.94** | 6.48** | 4.92 | 6.33* | 5.52 | 4.60 | 5.69* | 6.24** |
| **OC (C)** | 0.79 | -2.18 | -2.40 | -1.61 | 1.61 | 0.25 | 0.36 | -0.71 | -0.20 | 1.64 | -1.37 | 0.80 | -1.57 | -0.44 | -1.20 | 1.58 | -0.58 | 1.60 | 1.78 | 1.11 | 1.72 | -0.64 | -0.55 | -0.79 | -1.04 | 1.18 | 1.05 |
| **Fe (tot)** | 895.10 | 0.18 | 0.07 | 0.45 | 0.12** | 0.25 | 0.26* | 0.27 | 0.05 | 0.14* | 0.11* | 0.09 | -0.11 | 0.08 | 0.17 | 0.12* | 0.03 | 0.10* | 0.11* | 0.11* | 0.12** | -0.12 | 0.01 | -0.11 | -0.01 | 0.09 | 0.10 |
| **Fe (sol)** | 32.09 | 1.56 | 1.48 | 1.70 | 3.43* | 2.19 | 2.23 | 1.51 | 0.81 | 2.68 | 1.42 | 2.92 | 0.94 | 0.60 | 1.82 | 2.95 | 1.75 | 3.02 | 2.81 | 2.88 | 2.96 | 0.51 | 1.82 | 1.13 | 1.31 | 2.47 | 2.26 |
| **Cu (tot)** | 57.96 | 0.27 | 0.14 | 0.35 | 0.17** | 0.33* | 0.33* | 0.57 | 0.08 | 0.21* | 0.31 | 0.14 | 0.16* | 0.12 | 0.26 | 0.17* | 0.08 | 0.15* | 0.16* | 0.16* | 0.17** | -0.05 | 0.18 | 0.03 | 0.04 | 0.14* | 0.14 |
| **Cu (sol)** | 8.65 | 0.10 | 0.10 | 0.12 | 0.19* | 0.16 | 0.16 | 0.13 | 0.03 | 0.20 | 0.09 | 0.16 | 0.07 | 0.18* | 0.12 | 0.19* | 0.11 | 0.18* | 0.18 | 0.17 | 0.18* | 0.08 | 0.15 | 0.10 | 0.08 | 0.16 | 0.15 |
| **Ni (tot)** | 3.53 | -0.04 | 0.07 | 0.30 | 0.92* | 0.96 | 1.00 | 0.50 | 0.32 | 1.14 | -0.52 | 0.65 | -0.67 | 0.48 | 0.90* | 0.92 | 0.05 | 0.84 | 0.87 | 0.92* | 1.05* | -1.00 | -0.26 | -0.91 | -0.03 | 0.77 | 0.73 |
| **Ni (sol)** | 1.82 | -0.44 | 0.08 | -0.63 | 1.85 | -0.11 | -0.15 | -0.57 | 1.21 | 0.92 | -1.06 | -0.23 | -1.38 | -0.34 | -0.33 | 1.33 | -5.63 | -6.52 | 0.88 | 2.25 | 1.98 | -2.19 | -3.05 | -2.72 | -1.27 | 0.50 | 0.53 |
| **V (tot)** | 2.04 | 1.33 | 1.09 | 1.61 | 1.62** | 1.90 | 1.99 | 1.52 | 1.00 | 1.67* | 1.24 | 1.28 | 0.93 | 1.16 | 1.49 | 2.30** | 1.54** | 1.08 | 1.53* | 1.59** | 1.63** | -0.70 | -0.48 | -0.77 | 0.52 | 1.28 | 1.35* |
| **V (sol) ^b^** | 1.94 | 7.55* | 8.04** | 7.34* | 7.98** | 7.91** | 7.67* | 7.62* | 10.54** | 7.86** | 7.28* | 8.09** | 7.33* | 7.81** | 7.55* | 12.94** | 5.45 | 7.90** | 7.67* | 8.47** | 7.62* | 0.76 | -0.54 | 0.03 | 6.72* | 6.95* | 7.57* |
| **Endotoxin** | 0.19 | 0.04 | 0.03 | 0.05 | -0.01 | 0.06 | 0.06 | -0.01 | 0.04 | -0.06 | -0.01 | -0.01 | -0.01 | -0.02 | -0.02 | -0.05 | -0.01 | -0.03 | 0.01 | 0.00 | 0.01 | 0.09 | 0.08 | 0.09 | 0.07 | 0.05 | 0.05 |
| **NO_3_^- a^** | 5.19 | 1.21 | 0.30 | 1.72 | 1.41 | 2.02 | 2.28 | 2.17 | -0.54 | 1.71 | 2.35 | 2.24 | 2.18 | 1.54 | 2.46 | 2.69 | 2.64 | 3.00 | 1.88 | 1.89 | 0.97 | 2.93 | 3.10 | 3.01 | 1.18 | 0.79 | 1.69 |
| **SO_4_^2- a^** | 2.99 | 3.07 | 2.53 | 3.35 | 2.05 | 3.32 | 3.53 | 3.31 | 1.64 | 2.92 | 3.73 | 2.93 | 3.57 | 2.50 | 4.08 | 3.09 | 3.51 | 2.27 | 2.50 | 1.79 | 2.51 | 3.10 | 2.98 | 3.05 | 2.33 | 1.94 | 2.62 |
| **OP^AA^** | 19.08 | 0.52* | 0.36 | 0.56** | 0.33** | 0.66** | 0.62** | 0.69** | 0.17 | 0.30** | 0.51* | 0.26 | 0.35 | 0.21 | 0.48* | 0.29** | 0.36** | 0.28** | 0.34** | 0.29** | 0.31** | 0.30** | 0.57 | 0.84 | 0.37* | 0.29** | 0.30** |
| **OP^GSH^** | 15.53 | 0.19 | 0.09 | 0.34 | 0.22** | 0.41** | 0.36* | 0.30 | 0.07 | 0.20 | 0.16 | 0.12 | -0.02 | 0.07 | 0.23 | 0.21** | 0.23 | 0.18* | 0.22** | 0.19** | 0.20** | -0.22 | 0.20** | -0.68 | 0.19 | 0.19* | 0.19* |
| **OP^TOTAL^** | 38.71 | 0.51 | 0.27 | 0.66* | 0.31** | 0.68** | 0.61** | 0.77* | 0.13 | 0.30* | 0.49 | 0.21 | 0.22 | 0.17 | 0.45 | 0.29** | 0.36* | 0.26** | 0.32** | 0.27** | 0.29** | -0.55 | 1.16 | 0.28** | 0.34 | 0.27** | 0.28** |
| **O_3_** | 9.74 | -7.38 | -6.32 | -8.30 | -9.59** | -17.13** | -14.17** | -9.81 | -5.15 | -8.75** | -7.96 | -6.66* | -6.31 | -6.24 | -7.85 | -8.07** | -5.83 | -6.83* | -8.73** | -7.57** | -7.69** | 2.53 | -0.55 | 1.87 | -7.76** | -6.59 | -8.68* |
| **NO_2_** | 10.54 | 8.11 | 7.56 | 8.58 | 18.64** | 8.63 | 8.60 | 11.49* | 6.33 | 12.79* | 11.27 | 12.13* | 11.25 | 11.85* | 11.97* | 13.23* | 10.85 | 11.65* | 12.07* | 10.21 | 10.29 | 4.38 | 4.67 | 4.44 | 4.10 | 10.81 | 14.91 |
| **NO_X_** | 28.05 | 1.91 | 1.76 | 2.27 | 13.31* | 2.60 | 2.60 | 3.86 | 2.53 | 6.55 | 3.70 | 4.79 | 3.24 | 3.90 | 4.69 | 7.18 | 4.31 | 6.63 | 7.41 | 5.76 | 6.16 | 0.13 | 1.08 | 0.44 | -2.19 | -3.53 | 6.02 |

^a^ measured in PM_2.5_; ^b^ associations disappeared or decreased substantially after excluding 1% of influential observations. * p<0.10, ** p<0.05. “Tot” denotes total, whereas “sol” water-soluble metal extraction. “C” is the coarse and “F” is the fine PM fraction. Fields in light shading indicate Spearman’s R above 0.7. In each row effect estimates for the indicated pollutant in two-pollutant models are presented. The effect estimates in a single-pollutant model are presented on the diagonal (dark shading). All models were adjusted for the use of oral contraceptives and the use of oral contraceptives on the sampling day or the day before, temperature, relative humidity, season and adjustment pollutant (indicated in the header of each column). Estimates are percentage increases above population-average baseline expressed per outdoor-sites IQR. N=170, except all models including OP where N=153 and all models including EC (C), OC (C) and trace metals where N=166.
