# Supplementary material for: Composition of PM Affects Acute Vascular Inflammatory and Coagulative Markers - The RAPTES Project
Source: PLoS One. 2013 Mar 13;8(3):e58944. doi: 10.1371/journal.pone.0058944 (PMC3596332; doi:10.1371/journal.pone.0058944)
Supplement: Table S10 — Two-pollutant models of associations between exposure to air pollution and percentage changes (25 h post-pre) in fibrinogen (all sites). (DOC) [file pone.0058944.s011.doc]

**Table S10** Two-pollutant models of associations between exposure to air pollution and percentage changes (25h post-pre) in fibrinogen (all sites).

|  | **IQR** | **A D J U S T M E N T P O L L U T A N T S** | | | | | | | | | | | | | | | | | | | | | | | | | |
| --- | --- | --- | --- | --- | --- | --- | --- | --- | --- | --- | --- | --- | --- | --- | --- | --- | --- | --- | --- | --- | --- | --- | --- | --- | --- | --- | --- |
|  |  | **PM_10_** | **PM_2.5_** | **PM_2.5_**-**_10_** | **PNC** | **Abs.^a^** | **EC (F)** | **EC (C)** | **OC (F)** | **OC (C)** | **Fe (tot)** | **Fe (sol)** | **Cu (tot)** | **Cu (sol)** | **Ni (tot)** | **Ni (sol)** | **V (tot)** | **V (sol)** | **End.** | **NO_3_^- a^** | **SO_4_^2- a^** | **OP^AA^** | **OP^GSH^** | **OP^TOTAL^** | **O_3_** | **NO_2_** | **NO_X_** |
| **PM_10_** | 13.50 | 0.10 | -0.18 | 0.61 | 0.11 | 0.27 | 0.30* | 0.16 | 0.07 | 0.02 | 0.42 | 0.07 | 0.11 | 0.05 | 0.13 | 0.10 | 0.07 | 0.10 | 0.10 | 0.08 | 0.11 | 0.09 | 0.04 | 0.06 | 0.10 | 0.11 | 0.12 |
| **PM_2.5_** | 11.54 | 0.68 | 0.27 | 0.52 | 0.28* | 0.57* | 0.59* | 0.49 | 0.22 | 0.14 | 0.59 | 0.22 | 0.44 | 0.20 | 0.40 | 0.27* | 0.25 | 0.28* | 0.27 | 0.18 | 0.26 | 0.38 | 0.28 | 0.33 | 0.33 | 0.28 | 0.32* |
| **PM_2.5_**-**_10_** | 8.23 | -0.48 | -0.11 | 0.09 | 0.10 | 0.20 | 0.25 | -0.02 | 0.05 | 0.00 | 0.12 | 0.05 | -0.05 | 0.03 | 0.03 | 0.08 | 0.02 | 0.08 | 0.09 | 0.08 | 0.10 | 0.02 | -0.09 | -0.05 | 0.05 | 0.09 | 0.10 |
| **PNC** | 32,906 | -1.12 | -1.06 | -1.14 | -0.92 | -1.75 | -1.62 | -1.36 | -0.99 | -0.93 | -1.27 | -1.54 | -1.27 | -1.27 | -1.08 | -1.20 | -1.23 | -1.07 | -1.17 | -0.35 | -0.46 | -1.24 | -1.28 | -1.27 | -1.49 | -1.40 | -1.48 |
| **Absorbance^a^** | 3.49 | -1.35 | -1.11 | -0.98 | 0.87 | 0.44 | 4.53 | -1.20 | 0.10 | -0.12 | -0.96 | 0.06 | -1.07 | -0.10 | -0.38 | 0.41 | -0.29 | 0.43 | 0.46 | 0.47 | 0.63 | -0.52 | -0.91 | -0.88 | -0.75 | 0.47 | 0.70 |
| **EC (F)** | 4.35 | -1.82 | -1.36 | -1.58 | 0.84 | -4.45 | 0.40 | -1.57 | 0.06 | -0.23 | -1.74 | -0.03 | -1.63 | -0.17 | -0.75 | 0.35 | -0.63 | 0.37 | 0.40 | 0.52 | 0.66 | -1.01 | -1.28 | -1.33 | -1.00 | 0.40 | 0.58 |
| **EC (C)** | 0.40 | -0.08 | -0.14 | 0.15 | 0.16* | 0.34 | 0.37* | 0.13 | 0.07 | 0.03 | 0.22 | 0.07 | -0.01 | 0.03 | 0.09 | 0.13 | 0.06 | 0.13 | 0.13 | 0.12 | 0.14* | 0.06 | -0.04 | -0.03 | 0.11 | 0.13 | 0.17 |
| **OC (F)** | 1.82 | 0.41 | 0.24 | 0.50 | 0.75 | 0.67 | 0.70 | 0.54 | 0.73 | 0.47 | 0.59 | 0.52 | 0.52 | 0.40 | 0.56 | 0.77 | 0.57 | 0.90* | 0.73 | 0.34 | 0.56 | 0.71 | 0.64 | 0.66 | 0.51 | 0.77 | 0.78 |
| **OC (C)** | 0.79 | 0.56 | 0.41 | 0.65 | 0.62* | 0.69* | 0.72* | 0.56 | 0.51 | 0.64* | 0.61 | 0.54 | 0.55 | 0.49 | 0.60 | 0.63* | 0.52 | 0.64* | 0.66** | 0.39 | 0.64** | 0.70* | 0.73 | 0.71 | 0.58 | 0.64* | 0.68** |
| **Fe (tot)** | 895.10 | -0.05 | -0.02 | -0.01 | 0.02 | 0.03 | 0.05* | -0.01 | 0.01 | 0.00 | 0.01 | 0.01 | -0.04 | 0.00 | 0.00 | 0.01 | 0.00 | 0.01 | 0.01 | 0.01 | 0.02* | -0.01 | -0.02 | -0.02 | 0.01 | 0.01 | 0.02 |
| **Fe (sol)** | 32.09 | 0.31 | 0.25 | 0.35 | 0.63* | 0.47 | 0.49 | 0.32 | 0.30 | 0.33 | 0.36 | 0.49 | 0.31 | 0.17 | 0.35 | 0.48 | 0.36 | 0.49 | 0.50 | 0.47 | 0.49 | 0.35 | 0.32 | 0.32 | 0.38 | 0.48 | 0.56 |
| **Cu (tot)** | 57.96 | 0.00 | -0.02 | 0.03 | 0.02* | 0.05 | 0.06* | 0.02 | 0.01 | 0.01 | 0.08 | 0.01 | 0.02 | 0.01 | 0.02 | 0.02 | 0.01 | 0.02 | 0.02 | 0.02 | 0.02* | 0.01 | 0.00 | 0.00 | 0.02 | 0.02 | 0.03 |
| **Cu (sol)** | 8.65 | 0.02 | 0.01 | 0.02 | 0.03* | 0.03 | 0.03 | 0.02 | 0.02 | 0.02 | 0.02 | 0.02 | 0.02 | 0.03* | 0.02 | 0.03 | 0.02 | 0.03 | 0.03 | 0.02 | 0.03 | 0.03 | 0.03 | 0.03 | 0.02 | 0.03 | 0.04* |
| **Ni (tot)** | 3.53 | -0.04 | -0.09 | 0.10 | 0.13 | 0.19 | 0.24 | 0.05 | 0.07 | 0.01 | 0.13 | 0.08 | 0.01 | 0.05 | 0.13 | 0.13 | 0.06 | 0.13 | 0.13 | 0.14 | 0.18* | 0.08 | 0.04 | 0.04 | 0.10 | 0.13 | 0.15 |
| **Ni (sol)** | 1.82 | 0.02 | 0.07 | 0.02 | 0.47 | 0.12 | 0.16 | 0.00 | 0.26 | 0.12 | 0.00 | 0.02 | -0.06 | 0.01 | 0.05 | 0.28 | -0.56 | -0.14 | 0.24 | 0.64 | 0.53 | -0.22 | -0.41 | -0.32 | 0.03 | 0.27 | 0.28 |
| **V (tot)** | 2.04 | 0.08 | 0.02 | 0.16 | 0.22* | 0.26 | 0.32 | 0.12 | 0.14 | 0.09 | 0.18 | 0.14 | 0.10 | 0.12 | 0.13 | 0.27 | 0.20 | 0.18 | 0.20 | 0.21* | 0.22* | -0.05 | -0.15 | -0.11 | 0.17 | 0.20 | 0.21 |
| **V (sol)** | 1.94 | 0.49 | 0.56 | 0.47 | 0.58 | 0.54 | 0.52 | 0.50 | 0.81 | 0.53 | 0.47 | 0.57 | 0.47 | 0.53 | 0.49 | 0.64 | 0.15 | 0.54 | 0.52 | 0.74 | 0.42 | -0.90 | -1.09 | -0.99 | 0.42 | 0.53 | 0.54 |
| **Endotoxin** | 0.19 | 0.00 | 0.00 | 0.00 | -0.01 | 0.00 | 0.00 | 0.00 | 0.00 | -0.01 | 0.00 | 0.00 | 0.00 | 0.00 | 0.00 | 0.00 | 0.00 | 0.00 | 0.00 | -0.01 | 0.00 | 0.01 | 0.01 | 0.01 | 0.00 | 0.00 | 0.00 |
| **NO_3_^- a^** | 5.19 | 0.90* | 0.81 | 0.96* | 0.94* | 1.00** | 1.03** | 0.97** | 0.86 | 0.77 | 0.99** | 0.97** | 0.97** | 0.89** | 1.00** | 1.09** | 1.03** | 1.05** | 1.03** | 0.98** | 0.54 | 0.98* | 0.98* | 0.98* | 0.92* | 1.03** | 0.99** |
| **SO_4_^2- a b^** | 2.99 | 1.40** | 1.31** | 1.44** | 1.27** | 1.45** | 1.47** | 1.40** | 1.23* | 1.34** | 1.45** | 1.34** | 1.43** | 1.27** | 1.53** | 1.41** | 1.42** | 1.31** | 1.34** | 0.90 | 1.33** | 1.21* | 1.21* | 1.21* | 1.30** | 1.34** | 1.34** |
| **OP^AA^** | 19.08 | 0.01 | -0.02 | 0.03 | 0.04 | 0.05 | 0.07 | 0.02 | 0.01 | 0.01 | 0.05 | 0.02 | 0.02 | 0.01 | 0.02 | 0.04 | 0.04 | 0.03 | 0.04 | 0.03 | 0.03 | 0.03 | 0.00 | -0.04 | 0.03 | 0.03 | 0.04 |
| **OP^GSH^** | 15.53 | 0.02 | 0.00 | 0.05 | 0.03 | 0.06 | 0.06* | 0.03 | 0.01 | 0.00 | 0.06 | 0.02 | 0.03 | 0.01 | 0.02 | 0.03* | 0.04 | 0.03* | 0.03 | 0.03 | 0.03 | 0.03 | 0.03 | 0.04 | 0.04 | 0.03 | 0.03 |
| **OP^TOTAL^** | 38.71 | 0.02 | -0.01 | 0.05 | 0.04 | 0.07 | 0.08 | 0.04 | 0.02 | 0.01 | 0.07 | 0.02 | 0.03 | 0.01 | 0.03 | 0.04* | 0.05 | 0.04* | 0.04 | 0.03 | 0.04 | 0.08 | -0.01 | 0.04 | 0.04 | 0.04 | 0.04 |
| **O_3_** | 9.74 | 0.01 | 0.27 | -0.42 | -1.19 | -1.77 | -1.89 | -0.14 | -0.56 | -0.20 | -0.33 | -0.48 | -0.11 | -0.32 | -0.31 | -0.80 | -0.15 | -0.75 | -0.92 | -0.70 | -0.82 | 0.04 | 0.35 | 0.28 | -0.88 | -1.09 | -1.37 |
| **NO_2_** | 10.54 | -0.11 | -0.29 | -0.01 | 1.10 | -0.17 | -0.02 | -0.06 | -0.28 | -0.01 | -0.02 | 0.04 | -0.03 | 0.01 | 0.05 | 0.20 | -0.09 | 0.12 | 0.23 | -0.42 | -0.05 | -0.33 | -0.36 | -0.35 | -0.71 | 0.29 | 0.61 |
| **NO_X_** | 28.05 | -0.60 | -0.71 | -0.49 | 0.91 | -0.74 | -0.48 | -0.74 | -0.33 | -0.41 | -0.54 | -0.54 | -0.66 | -0.71 | -0.45 | -0.02 | -0.40 | -0.02 | 0.01 | -0.05 | 0.14 | -0.47 | -0.51 | -0.51 | -1.10 | -0.31 | 0.10 |

For explanation see Table S9.
