# Supplementary material for: Composition of PM Affects Acute Vascular Inflammatory and Coagulative Markers - The RAPTES Project
Source: PLoS One. 2013 Mar 13;8(3):e58944. doi: 10.1371/journal.pone.0058944 (PMC3596332; doi:10.1371/journal.pone.0058944)
Supplement: Table S11 — Two-pollutant models of associations between exposure to air pollution and percentage changes (25 h post-pre) in platelet counts (all sites). (DOC) [file pone.0058944.s012.doc]

**Table S11** Two-pollutant models of associations between exposure to air pollution and percentage changes (25h post-pre) in platelet counts (all sites).

|  | **IQR** | **A D J U S T M E N T P O L L U T A N T S** | | | | | | | | | | | | | | | | | | | | | | | | | |
| --- | --- | --- | --- | --- | --- | --- | --- | --- | --- | --- | --- | --- | --- | --- | --- | --- | --- | --- | --- | --- | --- | --- | --- | --- | --- | --- | --- |
|  |  | **PM_10_** | **PM_2.5_** | **PM_2.5_**-**_10_** | **PNC** | **Abs.^a^** | **EC (F)** | **EC (C)** | **OC (F)** | **OC (C)** | **Fe (tot)** | **Fe (sol)** | **Cu (tot)** | **Cu (sol)** | **Ni (tot)** | **Ni (sol)** | **V (tot)** | **V (sol)** | **End.** | **NO_3_^- a^** | **SO_4_^2- a^** | **OP^AA^** | **OP^GSH^** | **OP^TOTAL^** | **O_3_** | **NO_2_** | **NO_X_** |
| **PM_10_** | 13.50 | 0.04 | 0.12 | -0.11 | 0.05 | 0.25* | 0.23* | 0.39* | 0.07 | -0.17** | 0.53* | 0.06 | 0.32 | 0.05 | 0.25 | 0.02 | 0.03 | 0.03 | 0.05 | 0.03 | 0.04 | 0.30** | -0.05 | 0.17 | 0.25** | 0.07 | 0.10 |
| **PM_2.5_** | 11.54 | -0.19 | 0.07 | -0.09 | 0.09 | 0.33 | 0.30 | 0.36 | 0.17 | -0.39** | 0.24 | 0.13 | 0.29 | 0.09 | 0.27 | 0.04 | 0.03 | 0.06 | 0.08 | 0.00 | 0.08 | 0.37 | -0.15 | 0.08 | 0.38* | 0.15 | 0.21 |
| **PM_2.5_**-**_10_** | 8.23 | 0.14 | 0.07 | 0.04 | 0.05 | 0.25** | 0.25** | 0.36* | 0.06 | -0.14** | 0.78** | 0.06 | 0.31* | 0.05 | 0.26* | 0.02 | 0.04 | 0.03 | 0.05 | 0.04 | 0.04 | 0.29** | 0.00 | 0.22 | 0.25** | 0.06 | 0.09* |
| **PNC** | 32,906 | -1.26 | -1.21 | -1.28 | -1.15 | -1.47 | -1.44 | -1.27 | -1.15 | -1.15 | -1.27 | -1.20 | -1.25 | -1.23 | -1.20 | -1.45* | -1.28 | -1.19 | -1.18 | -0.75 | -1.09 | -0.70 | -0.81 | -0.76 | -1.18 | -0.51 | -0.37 |
| **Absorbance ^a^** | 3.49 | -1.67* | -0.94 | -1.79* | 0.31 | -0.05 | 0.85 | -0.63 | 0.01 | -0.99** | -1.01 | 0.02 | -0.69 | -0.18 | -0.29 | -0.21 | -0.61 | -0.07 | 0.03 | -0.01 | -0.01 | 0.35 | -1.21 | -0.47 | 0.45 | 0.33 | 0.73 |
| **EC (F)** | 4.35 | -1.77* | -0.98 | -2.01** | 0.32 | -1.01 | -0.08 | -0.70 | -0.03 | -1.06** | -1.27 | -0.02 | -0.84 | -0.21 | -0.40 | -0.27 | -0.77 | -0.10 | 0.01 | 0.03 | -0.02 | 0.21 | -1.20 | -0.58 | 0.31 | 0.29 | 0.70 |
| **EC (C)** | 0.40 | -0.51* | -0.19 | -0.49* | 0.03 | 0.12 | 0.11 | 0.01 | 0.04 | -0.21** | -0.17 | 0.05 | -0.12 | -0.01 | 0.02 | -0.02 | -0.08 | 0.01 | 0.02 | 0.01 | 0.01 | 0.21 | -0.46** | -0.15 | 0.13 | 0.03 | 0.09 |
| **OC (F)** | 1.82 | -0.43 | -0.47 | -0.39 | -0.10 | -0.12 | -0.11 | -0.25 | -0.12 | -0.65 | -0.27 | -0.08 | -0.27 | -0.39 | -0.21 | -0.15 | -0.26 | -0.17 | -0.09 | -0.50 | -0.14 | -0.06 | -0.53 | -0.31 | -0.06 | 0.11 | 0.08 |
| **OC (C)** | 0.79 | 1.44** | 1.44** | 1.37** | 0.78** | 1.20** | 1.16** | 1.29** | 0.96** | 0.79** | 1.31** | 0.91** | 1.32** | 1.11** | 1.50** | 0.75** | 0.99** | 0.79** | 0.78** | 0.63** | 0.79** | 1.10** | 0.96** | 1.06** | 1.24** | 0.84** | 0.94** |
| **Fe (tot)** | 895.10 | -0.08* | -0.01 | -0.13** | 0.00 | 0.02 | 0.03 | 0.02 | 0.01 | -0.02** | 0.00 | 0.01 | 0.02 | 0.00 | 0.02 | 0.00 | -0.01 | 0.00 | 0.00 | 0.00 | 0.00 | 0.03 | -0.03 | 0.01 | 0.02 | 0.01 | 0.01 |
| **Fe (sol)** | 32.09 | -0.27 | -0.25 | -0.27 | 0.00 | -0.11 | -0.09 | -0.22 | -0.07 | -0.37 | -0.20 | -0.10 | -0.23 | -0.45 | -0.15 | -0.21 | -0.19 | -0.10 | -0.09 | -0.09 | -0.10 | -0.17 | -0.45 | -0.35 | -0.06 | -0.06 | 0.06 |
| **Cu (tot)** | 57.96 | -0.07 | -0.02 | -0.07 | 0.01 | 0.02 | 0.02 | 0.02 | 0.01 | -0.03** | -0.02 | 0.01 | 0.00 | 0.00 | 0.01 | 0.00 | -0.01 | 0.00 | 0.00 | 0.00 | 0.00 | 0.03 | -0.05* | -0.02 | 0.02 | 0.01 | 0.01 |
| **Cu (sol)** | 8.65 | -0.01 | 0.00 | -0.01 | 0.01 | 0.01 | 0.01 | 0.01 | 0.01 | -0.03* | 0.00 | 0.02 | 0.00 | 0.00 | 0.00 | 0.00 | 0.00 | 0.00 | 0.00 | 0.00 | 0.00 | 0.01 | -0.03 | -0.01 | 0.01 | 0.01 | 0.02 |
| **Ni (tot)** | 3.53 | -0.32 | -0.14 | -0.37 | 0.01 | 0.05 | 0.06 | -0.01 | 0.03 | -0.28** | -0.13 | 0.03 | -0.07 | -0.01 | 0.01 | -0.02 | -0.09 | 0.01 | 0.01 | 0.01 | 0.02 | 0.15 | -0.19 | -0.02 | 0.08 | 0.02 | 0.06 |
| **Ni (sol)** | 1.82 | 0.59 | 0.61 | 0.59 | 0.85 | 0.72 | 0.73 | 0.68 | 0.65 | 0.42 | 0.67 | 0.76 | 0.69 | 0.66 | 0.68 | 0.64 | 0.75 | 1.16* | 0.70 | 0.95* | 0.71 | 0.69 | 0.50 | 0.61 | 0.78 | 0.73 | 0.79 |
| **V (tot)** | 2.04 | 0.01 | 0.03 | -0.01 | 0.08 | 0.19 | 0.21 | 0.15 | 0.08 | -0.14 | 0.16 | 0.09 | 0.16 | 0.06 | 0.16 | -0.04 | 0.06 | 0.08 | 0.07 | 0.08 | 0.07 | 0.28* | 0.06 | 0.19 | 0.29 | 0.10 | 0.13 |
| **V (sol)** | 1.94 | -0.08 | -0.06 | -0.10 | -0.03 | -0.07 | -0.07 | -0.07 | -0.13 | -0.08 | -0.08 | -0.08 | -0.08 | -0.07 | -0.07 | -0.81 | -0.22 | -0.07 | -0.05 | 0.10 | -0.14 | 0.67 | 0.60 | 0.65 | -0.04 | 0.07 | 0.02 |
| **Endotoxin** | 0.19 | 0.01 | 0.01 | 0.01 | 0.00 | 0.01 | 0.01 | 0.00 | 0.01 | 0.00 | 0.00 | 0.00 | 0.00 | 0.00 | 0.00 | 0.01 | 0.01 | 0.00 | 0.01 | 0.00 | 0.01 | 0.00 | 0.01 | 0.01 | 0.00 | 0.00 | 0.00 |
| **NO_3_^- a^** | 5.19 | 0.72* | 0.74* | 0.74** | 0.64* | 0.74** | 0.75** | 0.85** | 0.92** | 0.52 | 0.85** | 0.85** | 0.85** | 0.86** | 0.86** | 0.99** | 0.87** | 0.86** | 0.73* | 0.74** | 0.90* | 0.38 | 0.35 | 0.37 | 0.77** | 0.98** | 0.78** |
| **SO_4_^2- a^** | 2.99 | 0.33 | 0.30 | 0.35 | 0.18 | 0.29 | 0.29 | 0.38 | 0.31 | 0.41 | 0.40 | 0.37 | 0.39 | 0.37 | 0.40 | 0.45 | 0.40 | 0.39 | 0.29 | -0.33 | 0.29 | 0.00 | 0.03 | 0.02 | 0.30 | 0.39 | 0.28 |
| **OP^AA^** | 19.08 | -0.09** | -0.04 | -0.09** | 0.01 | -0.01 | 0.00 | -0.04 | 0.01 | -0.04* | -0.06 | 0.01 | -0.04 | 0.00 | -0.02 | 0.00 | -0.03 | 0.01 | 0.01 | 0.00 | 0.01 | 0.01 | -0.15** | -0.31** | 0.00 | 0.01 | 0.02 |
| **OP^GSH^** | 15.53 | 0.03 | 0.03 | 0.02 | 0.02 | 0.06* | 0.05* | 0.10** | 0.03* | -0.01 | 0.06 | 0.03* | 0.08** | 0.04* | 0.05 | 0.01 | 0.01 | 0.02 | 0.02 | 0.02 | 0.02 | 0.13** | 0.02 | 0.25** | 0.04* | 0.02 | 0.03* |
| **OP^TOTAL^** | 38.71 | -0.04 | 0.01 | -0.06 | 0.02 | 0.03 | 0.04 | 0.05 | 0.02 | -0.03 | 0.00 | 0.03 | 0.04 | 0.03 | 0.02 | 0.01 | -0.01 | 0.02 | 0.02 | 0.01 | 0.02 | 0.32** | -0.30** | 0.02 | 0.03 | 0.02 | 0.03 |
| **O_3_** | 9.74 | 2.37** | 1.56 | 2.46** | -0.05 | 0.74 | 0.53 | 0.99 | 0.17 | 1.50** | 1.42 | 0.18 | 1.00 | 0.42 | 0.66 | 0.45 | 1.39 | 0.22 | 0.14 | 0.32 | 0.21 | -0.32 | 1.16 | 0.44 | 0.21 | -0.44 | -0.67 |
| **NO_2_** | 10.54 | -1.93** | -1.98** | -1.89** | -1.40 | -2.01** | -1.91** | -1.65* | -1.76* | -1.83** | -1.67* | -1.56* | -1.65* | -1.60* | -1.61* | -1.68* | -1.75* | -1.59* | -1.71* | -2.28** | -1.77** | -1.18 | -1.30 | -1.24 | -2.08** | -1.69* | -0.89 |
| **NO_X_** | 28.05 | -1.93** | -1.89** | -1.91** | -1.19 | -2.27** | -2.10** | -1.70** | -1.43* | -1.86** | -1.64** | -1.37* | -1.65** | -1.59** | -1.49* | -1.46** | -1.57** | -1.31* | -1.50* | -1.48** | -1.39* | -1.37* | -1.67** | -1.53* | -1.98** | -0.79 | -1.40* |

For explanation see Table S9.
