# Supplementary material for: Composition of PM Affects Acute Vascular Inflammatory and Coagulative Markers - The RAPTES Project
Source: PLoS One. 2013 Mar 13;8(3):e58944. doi: 10.1371/journal.pone.0058944 (PMC3596332; doi:10.1371/journal.pone.0058944)
Supplement: Table S12 — Two-pollutant models of associations between exposure to air pollution and percentage changes (25 h post-pre) in von Willebrand Factor (all sites). (DOC) [file pone.0058944.s013.doc]

**Table S12** Two-pollutant models of associations between exposure to air pollution and percentage changes (25h post-pre) in von Willebrand Factor (all sites).

|  | **IQR** | **A D J U S T M E N T P O L L U T A N T S** | | | | | | | | | | | | | | | | | | | | | | | | | |
| --- | --- | --- | --- | --- | --- | --- | --- | --- | --- | --- | --- | --- | --- | --- | --- | --- | --- | --- | --- | --- | --- | --- | --- | --- | --- | --- | --- |
|  |  | **PM_10_** | **PM_2.5_** | **PM_2.5-10_** | **PNC** | **Abs.^a^** | **EC (F)** | **EC (C)** | **OC (F)** | **OC (C)** | **Fe (tot)** | **Fe (sol)** | **Cu (tot)** | **Cu (sol)** | **Ni (tot)** | **Ni (sol)** | **V (tot)** | **V (sol)** | **End.** | **NO_3_^- a^** | **SO_4_^2- a^** | **OP^AA^** | **OP^GSH^** | **OP^TOTAL^** | **O_3_** | **NO_2_** | **NO_X_** |
| **PM_10_** | 13.50 | 0.22** | -0.32 | 1.19* | 0.22** | 0.21 | 0.31 | 0.36 | -0.04 | 0.07 | 1.18** | 0.02 | 0.36 | -0.02 | 0.54* | 0.22** | 0.29 | 0.21** | 0.23** | 0.21** | 0.13 | 0.35 | 0.19 | 0.02 | 0.12 | 0.20* | 0.21* |
| **PM_2.5_** | 11.54 | 1.29 | 0.58** | 1.02* | 0.58** | 0.66 | 0.78* | 0.97 | -0.06 | 0.32 | 1.28** | 0.15 | 0.79 | 0.08 | 1.11** | 0.57** | 0.72* | 0.55** | 0.59** | 0.55** | 0.35 | 0.51 | 0.60 | 0.34 | 0.41 | 0.53** | 0.58** |
| **PM_2.5-10_** | 8.23 | -0.91 | -0.19 | 0.18* | 0.18* | 0.08 | 0.15 | 0.03 | 0.09 | 0.03 | 0.18 | 0.00 | 0.11 | -0.05 | 0.35 | 0.18* | 0.21 | 0.17* | 0.19* | 0.18* | 0.11 | 0.19 | -0.02 | 0.11 | 0.05 | 0.16* | 0.17* |
| **PNC** | 32,906 | 0.16 | 0.28 | 0.13 | -0.04 | -0.66 | -0.40 | 0.02 | -0.13 | 0.87 | 0.12 | -0.19 | 0.11 | -0.02 | 0.75 | 0.22 | 0.16 | 0.21 | 0.20 | 0.40 | 0.37 | -1.34 | -1.43 | -1.40 | -0.35 | -0.73 | -0.48 |
| **Absorbance ^a^** | 3.49 | 0.08 | -0.29 | 0.88 | 0.66 | 0.49 | 10.15* | 0.14 | -0.27 | 0.38 | 1.28 | 0.12 | 0.92 | -0.09 | 1.74 | 0.49 | 0.81 | 0.44 | 1.74** | 1.50** | 0.94 | -0.92 | -2.09 | -1.69 | 0.40 | 0.32 | 0.57 |
| **EC (F)** | 4.35 | -0.91 | -0.87 | -0.86 | 0.44 | -9.98* | 0.32 | -1.10 | -0.40 | 0.06 | -0.10 | -0.19 | -0.23 | -0.40 | 0.30 | 0.28 | 0.31 | 0.26 | 1.68* | 1.54* | 0.88 | -1.44 | -2.39 | -2.13 | -0.45 | 0.12 | 0.24 |
| **EC (C)** | 0.40 | -0.22 | -0.27 | 0.21 | 0.09 | 0.06 | 0.25 | 0.25* | -0.11 | 0.08 | 0.67 | 0.01 | 0.67 | -0.10 | 0.43 | 0.10 | 0.19 | 0.25* | 0.27* | 0.25* | 0.16 | 0.18 | 0.30 | -0.20 | 0.09 | 0.22 | 0.23 |
| **OC (F)** | 1.82 | 1.51 | 1.46 | 1.32 | 1.34* | 1.48* | 1.50* | 1.68* | 1.81** | 1.68** | 1.71* | 1.41 | 2.16** | 2.53** | 1.65* | 1.81** | 1.51* | 1.71** | 1.87** | 1.69* | 1.24 | 1.15 | 0.86 | 0.98 | 1.46* | 1.30* | 1.39* |
| **OC (C)** | 0.79 | -0.05 | -0.29 | 0.10 | 0.50 | 0.07 | 0.20 | 0.03 | 0.03 | 0.48 | 0.19 | 0.10 | 0.16 | -0.11 | 0.29 | 0.49 | 0.24 | 0.48 | 0.22 | -0.02 | 0.13 | 0.65 | 0.47 | 0.56 | 0.11 | 0.17 | 0.39 |
| **Fe (tot)** | 895.10 | -0.16* | -0.05 | -0.02 | 0.01 | -0.02 | 0.01 | -0.05 | -0.01 | 0.00 | 0.02 | 0.00 | -0.02 | -0.01 | 0.04 | 0.03 | 0.01 | 0.03 | 0.03 | 0.03 | 0.01 | -0.01 | -0.04 | -0.05 | -0.01 | 0.02 | 0.02 |
| **Fe (sol)** | 32.09 | 0.37 | 0.28 | 0.43 | 0.45 | 0.39 | 0.49 | 0.41 | -0.08 | 0.41 | 0.49 | 0.43 | 0.53 | 0.05 | 0.50 | 0.47 | 0.47 | 0.43 | 0.49 | 0.43 | 0.35 | 0.43 | 0.13 | 0.25 | 0.41 | 0.38 | 0.42 |
| **Cu (tot)** | 57.96 | -0.07 | -0.06 | -0.02 | 0.01 | -0.02 | 0.01 | -0.09 | -0.02 | 0.00 | 0.04 | -0.01 | 0.03 | -0.02 | 0.02 | 0.03 | 0.02 | 0.03 | 0.03 | 0.03 | 0.02 | 0.00 | -0.01 | -0.05 | 0.00 | 0.03 | 0.03 |
| **Cu (sol)** | 8.65 | 0.03 | 0.02 | 0.04 | 0.03 | 0.03 | 0.04 | 0.04 | -0.04 | 0.03 | 0.04 | 0.03 | 0.05 | 0.03 | 0.04 | 0.03 | 0.03 | 0.03 | 0.03 | 0.02 | 0.02 | 0.04 | 0.03 | 0.04 | 0.03 | 0.03 | 0.03 |
| **Ni (tot)** | 3.53 | -0.51 | -0.39 | -0.30 | 0.19 | -0.08 | -0.01 | -0.20 | 0.02 | -0.03 | -0.12 | -0.04 | 0.06 | -0.13 | 0.19 | 0.21 | 0.12 | 0.20 | 0.20 | 0.20 | 0.10 | 0.27 | 0.06 | 0.16 | -0.06 | 0.18 | 0.17 |
| **Ni (sol)** | 1.82 | -0.50 | -0.38 | -0.52 | -0.08 | -0.23 | -0.14 | -0.25 | -0.25 | -0.10 | -0.52 | -0.29 | -0.51 | -0.31 | -0.33 | -0.05 | -0.28 | 0.41 | -0.04 | 0.21 | -0.05 | -0.17 | -0.53 | -0.35 | -0.22 | -0.14 | -0.11 |
| **V (tot)** | 2.04 | -0.21 | -0.19 | -0.11 | 0.03 | -0.13 | -0.02 | -0.19 | -0.12 | -0.01 | -0.10 | -0.03 | -0.10 | -0.09 | 0.14 | 0.07 | 0.04 | 0.39* | 0.04 | 0.05 | 0.15 | 0.05 | -0.18 | -0.08 | -0.12 | 0.00 | 0.01 |
| **V (sol)** | 1.94 | -0.57 | -0.41 | -0.63 | -0.42 | -0.41 | -0.43 | -0.54 | 0.06 | -0.48 | -0.61 | -0.39 | -0.57 | -0.43 | -0.54 | -0.69 | -1.27 | -0.42 | -0.42 | -0.29 | -0.45 | -0.93 | -1.24 | -1.08 | -0.51 | -0.54 | -0.45 |
| **Endotoxin** | 0.19 | 0.01 | 0.01 | 0.01 | 0.01 | 0.02 | 0.02 | 0.01 | 0.01 | 0.00 | 0.01 | 0.01 | 0.01 | 0.01 | 0.00 | 0.00 | 0.00 | 0.00 | 0.01 | 0.01 | 0.00 | 0.02 | 0.02 | 0.02 | 0.01 | 0.01 | 0.01 |
| **NO_3_^- a^** | 5.19 | 0.59 | 0.29 | 0.74 | 0.72 | 0.84 | 0.92 | 0.81 | 0.25 | 0.73 | 0.85 | 0.71 | 0.81 | 0.62 | 0.86 | 0.75 | 0.73 | 0.70 | 0.64 | 0.66 | 0.55 | 0.87 | 0.87 | 0.87 | 0.62 | 0.57 | 0.65 |
| **SO_4_^2- a^** | 2.99 | 0.99 | 0.88 | 1.04 | 0.97 | 1.10 | 1.11 | 1.08 | 0.67 | 1.00 | 1.10 | 1.00 | 1.07 | 0.94 | 1.11 | 1.00 | 1.06 | 1.04 | 0.92 | 0.46 | 0.93 | 1.04 | 1.05 | 1.04 | 0.88 | 0.80 | 0.93 |
| **OP^AA^** | 19.08 | -0.05 | -0.05 | -0.01 | 0.05 | 0.05 | 0.06 | -0.03 | 0.02 | 0.02 | 0.03 | -0.01 | 0.04 | -0.03 | -0.01 | 0.05 | 0.04 | 0.05 | 0.06* | 0.05 | 0.03 | 0.05 | 0.00 | -0.03 | 0.04 | 0.05 | 0.06 |
| **OP^GSH^** | 15.53 | 0.00 | -0.01 | 0.05 | 0.05* | 0.08 | 0.08* | -0.03 | 0.03 | 0.00 | 0.10 | 0.01 | 0.04 | -0.01 | 0.04 | 0.05* | 0.06 | 0.05* | 0.05** | 0.05* | 0.03 | 0.03 | 0.05* | 0.02 | 0.07 | 0.05* | 0.05* |
| **OP^TOTAL^** | 38.71 | 0.01 | -0.02 | 0.02 | 0.06* | 0.09 | 0.10 | 0.09 | 0.03 | -0.01 | 0.11 | 0.00 | 0.12 | -0.02 | 0.02 | 0.06 | 0.06 | 0.06 | 0.07* | 0.06 | 0.03 | 0.07 | 0.01 | 0.06* | 0.07 | 0.06* | 0.06* |
| **O_3_** | 9.74 | 0.38 | 0.80 | -0.20 | -0.69 | -0.15 | -1.07 | 0.04 | 0.33 | -0.39 | -0.82 | -0.13 | -0.57 | 0.17 | -0.82 | -0.58 | -0.98 | -0.59 | -0.80 | -0.49 | -1.17 | 1.07 | 2.22 | 1.77 | -0.61 | -0.33 | -0.67 |
| **NO_2_** | 10.54 | 1.79 | 1.52 | 1.96 | 1.61 | 0.88 | 1.10 | 2.34 | 0.25 | 1.41 | 2.42 | 1.29 | 2.47 | 1.25 | 2.55 | 1.50 | 1.47 | 1.60 | 1.50 | 0.78 | 1.48 | 0.58 | 0.50 | 0.53 | 0.90 | 1.19 | 2.37 |
| **NO_X_** | 28.05 | 0.12 | -0.08 | 0.30 | 0.72 | -0.22 | 0.22 | 0.35 | -0.28 | 1.10 | 0.66 | 0.14 | 0.70 | -0.04 | 0.87 | 0.58 | 0.54 | 0.60 | 0.77 | 0.35 | 0.68 | -0.56 | -0.72 | -0.67 | -0.12 | -1.12 | 0.45 |

For explanation see Table S9.
