# Supplementary material for: Composition of PM Affects Acute Vascular Inflammatory and Coagulative Markers - The RAPTES Project
Source: PLoS One. 2013 Mar 13;8(3):e58944. doi: 10.1371/journal.pone.0058944 (PMC3596332; doi:10.1371/journal.pone.0058944)
Supplement: Table S13 — Two-pollutant models of associations between exposure to air pollution and percentage changes (25 h post-pre) in hs-CRP (outdoor sites). (DOC) [file pone.0058944.s014.doc]

**Table S13** Two-pollutant models of associations between exposure to air pollution and percentage changes (25h post-pre) in hs-CRP (outdoor sites).

|  | **IQR** | **A D J U S T M E N T P O L L U T A N T S** | | | | | | | | | | | | | | | | | | | | | | | | | |
| --- | --- | --- | --- | --- | --- | --- | --- | --- | --- | --- | --- | --- | --- | --- | --- | --- | --- | --- | --- | --- | --- | --- | --- | --- | --- | --- | --- |
|  |  | **PM_10_** | **PM_2.5_** | **PM_2.5_**-**_10_** | **PNC** | **Abs.^a^** | **EC (F)** | **EC (C)** | **OC (F)** | **OC (C)** | **Fe (tot)** | **Fe (sol)** | **Cu (tot)** | **Cu (sol)** | **Ni (tot)** | **Ni (sol)** | **V (tot)** | **V (sol)** | **End.** | **NO_3_^- a^** | **SO_4_^2- a^** | **OP^AA^** | **OP^GSH^** | **OP^TOTAL^** | **O_3_** | **NO_2_** | **NO_X_** |
| **PM_10_** | 13.50 | -0.69 | -16.60* | 4.66 | -1.09 | -0.39 | -0.75 | -0.48 | -1.13 | -0.02 | -0.58 | -1.48 | -0.52 | -0.19 | -0.40 | -0.89 | 0.29 | 0.36 | -1.18 | -6.81 | -1.22 | -0.54 | 1.91 | 2.12 | -0.94 | -1.33 | -0.76 |
| **PM_2.5_** | 11.54 | 21.42* | 0.36 | 3.97 | -0.30 | 0.56 | 0.11 | 0.63 | 0.29 | 2.70 | 0.29 | -0.31 | 0.45 | 0.89 | 0.77 | 0.34 | 1.48 | 1.71 | -0.12 | -3.86 | -0.38 | 1.99 | 2.59 | 4.41 | 0.19 | -0.14 | 0.32 |
| **PM_2.5_**-**_10_** | 8.23 | -12.92* | -10.47* | -6.36 | -5.87 | -5.51 | -5.68 | -6.44 | -6.41 | -14.03* | -5.51 | -7.28 | -5.84 | -5.75 | -6.54 | -6.76 | -5.27 | -5.70 | -7.03 | -10.34* | -6.09 | -6.26 | -1.57 | -5.26 | -7.39 | -7.70 | -6.82 |
| **PNC** | 32,906 | -8.31 | -8.15 | -7.52 | -8.04 | -12.04 | -16.15 | -16.05 | -8.09 | -7.22 | 1.76 | -8.36 | -7.51 | -3.46 | -7.92 | -6.83 | -7.29 | -6.92 | -7.71 | -8.10 | -7.35 | -11.41** | -11.30* | -11.61** | -10.32* | -12.14** | -13.42** |
| **Absorbance^a^** | 3.49 | -7.74 | -7.94 | -6.40 | 6.09 | -7.85 | 2.22 | -12.29 | -8.09 | -6.01 | 9.85 | -3.39 | 3.11 | 7.56 | -6.73 | -5.29 | -4.67 | -4.22 | -7.23 | -7.66 | -7.28 | -13.12* | -10.61 | -13.06* | -14.10* | -22.47** | -24.10** |
| **EC (F)** | 4.35 | -9.85 | -9.78 | -8.54 | 15.13 | -12.08 | -9.80 | -22.91 | -9.79 | -8.55 | 17.38 | -6.36 | 1.37 | 4.72 | -9.31 | -7.54 | -8.72 | -8.11 | -9.26 | -9.48 | -8.96 | -14.37* | -12.86 | -14.43* | -14.55 | -21.25** | -25.34** |
| **EC (C)** | 0.40 | -2.96 | -2.74 | -0.87 | 20.70 | 10.94 | 22.81 | -2.92 | -2.35 | -3.28 | 20.26 | 3.37 | 18.12 | 17.87 | -5.12 | -1.57 | -8.53 | -6.68 | -3.12 | -2.50 | -3.63 | -6.71 | -3.75 | -7.11 | -9.93 | -12.57 | -14.23 |
| **OC (F)** | 1.82 | 1.70 | 0.20 | 0.87 | -0.34 | 1.31 | 0.38 | 1.32 | 0.53 | 1.82 | 0.61 | -0.04 | 0.64 | 1.78 | 1.96 | 0.65 | 5.85 | 6.68 | 0.45 | -0.61 | -0.04 | 2.10 | 1.61 | 3.24 | 0.57 | -0.32 | 0.47 |
| **OC (C)** | 0.79 | -0.70 | -3.04 | 6.93 | -1.58 | -1.08 | -1.39 | -0.89 | -0.86 | -0.71 | -1.10 | -1.89 | -1.00 | -0.97 | -0.55 | -1.04 | 0.13 | -0.12 | -1.03 | -2.99 | -0.84 | -1.13 | 3.19 | 0.00 | -0.81 | -0.84 | -0.59 |
| **Fe (tot)** | 895.10 | -6.08 | -5.97 | -4.87 | -7.32 | -11.19 | -13.85 | -13.78* | -5.93 | -6.20 | -6.03 | -7.65 | -16.33 | -4.56 | -6.99 | -6.15 | -6.29 | -6.11 | -7.03 | -5.74 | -5.27 | -6.58 | -6.39 | -7.04 | -7.57 | -9.23* | -10.42* |
| **Fe (sol)** | 32.09 | -7.25 | -5.97 | -7.20 | 2.87 | -3.48 | -1.69 | -7.32 | -5.64 | -7.14 | 3.23 | -5.62 | 0.87 | 0.59 | -6.52 | -5.97 | -6.52 | -6.63 | -6.48 | -4.96 | -4.06 | -5.26 | -6.22 | -6.49 | -6.88 | -7.18 | -8.32 |
| **Cu (tot)** | 57.96 | -7.35 | -7.21 | -5.81 | 1.35 | -9.79 | -8.32 | -17.69 | -7.13 | -7.51 | 19.52 | -8.00 | -7.31 | -0.02 | -9.24 | -7.52 | -8.18 | -8.01 | -8.54 | -6.77 | -6.11 | -8.36 | -7.72 | -8.97 | -10.25 | -12.79* | -14.93* |
| **Cu (sol)** | 8.65 | -7.39 | -7.57 | -6.17 | -4.32 | -12.74 | -10.37 | -16.47 | -7.50 | -7.58 | -2.44 | -7.81 | -7.43 | -7.44 | -8.38 | -7.36 | -6.63 | -6.73 | -7.77 | -7.19 | -6.90 | -6.98 | -4.94 | -7.44 | -10.57 | -15.59** | -16.54** |
| **Ni (tot)** | 3.53 | -0.36 | -0.45 | 0.02 | -1.23 | -0.90 | -1.04 | -0.92 | -0.54 | -0.30 | -1.16 | -0.85 | -1.28 | -1.00 | -0.40 | -0.53 | 0.25 | -0.06 | -0.43 | -0.32 | 0.00 | -0.51 | -1.06 | -0.62 | -0.07 | -0.24 | -0.25 |
| **Ni (sol)** | 1.82 | -3.77 | -2.63 | -3.82 | 0.72 | -1.93 | -0.57 | -2.37 | -2.40 | -3.45 | 0.46 | 0.63 | 0.47 | -0.29 | -3.30 | -2.93 | -14.58* | -18.27** | -2.98 | -1.71 | -1.75 | -6.55 | -7.13 | -7.43 | -3.14 | -3.27 | -3.90 |
| **V (tot) ^b^** | 2.04 | 8.01** | 8.26** | 7.33* | 8.25** | 7.72** | 8.17** | 9.15** | 9.62** | 7.96** | 8.08** | 8.18** | 8.22** | 7.63** | 8.04** | 13.16** | 7.95** | 28.40* | 8.38** | 7.94** | 7.52* | -2.99 | -1.77 | -2.82 | 7.75** | 7.64** | 7.83** |
| **V (sol) ^b^** | 1.94 | 7.83* | 8.38* | 7.03 | 7.92* | 7.27 | 7.80* | 8.65* | 10.73** | 7.65* | 7.77* | 8.11* | 8.03* | 7.29 | 7.65* | 17.85** | -19.55 | 7.70* | 7.95* | 8.08* | 7.19 | -3.82 | -3.66 | -4.20 | 7.56* | 7.51 | 7.54 |
| **Endotoxin** | 0.19 | 0.06 | 0.05 | 0.07 | 0.01 | 0.02 | 0.01 | -0.01 | 0.05 | 0.02 | -0.05 | -0.03 | -0.04 | -0.02 | 0.01 | 0.00 | 0.04 | 0.03 | 0.05 | 0.05 | 0.05 | 0.08 | 0.14* | 0.09 | 0.06 | 0.07 | 0.07 |
| **NO_3_^- a^** | 5.19 | 8.59 | 5.11 | 4.59 | -0.09 | 0.98 | 0.54 | 1.48 | 1.41 | 3.31 | 0.61 | 0.51 | 0.92 | 1.28 | 1.52 | 1.30 | 1.56 | 2.05 | 0.95 | 1.25 | -0.22 | 5.95 | 3.47 | 6.54 | 1.09 | 0.92 | 1.24 |
| **SO_4_^2- a^** | 2.99 | 3.26 | 3.03 | 2.56 | 1.84 | 2.44 | 2.27 | 3.20 | 2.88 | 3.12 | 2.16 | 2.48 | 2.36 | 2.68 | 3.08 | 2.91 | 1.07 | 2.33 | 2.90 | 3.03 | 2.88 | 2.08 | 2.37 | 2.50 | 2.83 | 2.72 | 2.90 |
| **OP^AA^** | 19.08 | 1.39 | -1.60 | 3.15 | 1.00 | 2.55 | 1.42 | 3.60 | -0.34 | 3.91 | 3.03 | 1.98 | 3.10 | 3.74 | 3.00 | 1.92 | 3.31 | 2.99 | 0.56 | -5.17 | 0.02 | 0.72 | 3.02 | 13.91 | 1.25 | 1.50 | 1.12 |
| **OP^GSH^** | 15.53 | -7.92 | -8.12 | -4.80 | -0.12 | -1.98 | -2.07 | -2.81 | -5.69 | -7.15 | -0.99 | -3.38 | -1.67 | -1.99 | -4.62 | -3.12 | -3.77 | -3.65 | -9.88* | -7.75 | -6.04 | -7.86 | -5.75 | -10.06 | -5.76 | -6.08 | -5.64 |
| **OP^TOTAL^** | 38.71 | -5.13 | -8.50 | 2.40 | 1.22 | 2.68 | 1.15 | 3.68 | -3.10 | 2.15 | 3.36 | 0.72 | 3.22 | 4.18 | 1.81 | 0.88 | 2.50 | 2.14 | -2.40 | -10.02 | -2.32 | -18.45 | 6.22 | -1.22 | -0.70 | -1.04 | -0.72 |
| **O_3_** | 9.74 | -3.51 | -2.54 | -6.40 | -8.85 | -13.21 | -10.33 | -11.34 | -2.69 | -5.37 | -9.21 | -7.13 | -9.77 | -10.74 | -5.15 | -5.43 | -2.50 | -4.55 | -4.12 | -1.61 | -0.36 | 3.80 | -0.05 | 2.25 | -2.67 | 2.47 | -3.37 |
| **NO_2_** | 10.54 | 5.30 | 3.92 | 7.37 | 12.17 | 26.24** | 18.77* | 14.22 | 3.95 | 6.26 | 12.72 | 7.97 | 13.31 | 18.52* | 6.04 | 6.37 | 4.10 | 5.52 | 5.63 | 3.09 | 3.13 | -2.95 | 1.39 | -0.47 | 5.46 | 3.78 | 21.56 |
| **NO_X_** | 28.05 | 1.49 | 1.17 | 3.06 | 11.07 | 23.24** | 19.38* | 12.07 | 1.20 | 2.90 | 11.31 | 6.30 | 12.26 | 15.47* | 2.83 | 3.85 | 1.40 | 2.09 | 3.12 | 1.18 | 1.38 | -2.66 | -0.45 | -1.90 | -0.66 | -13.48 | 1.25 |

For explanation see Table S9.
