# Supplementary material for: Composition of PM Affects Acute Vascular Inflammatory and Coagulative Markers - The RAPTES Project
Source: PLoS One. 2013 Mar 13;8(3):e58944. doi: 10.1371/journal.pone.0058944 (PMC3596332; doi:10.1371/journal.pone.0058944)
Supplement: Table S15 — Two-pollutant models of associations between exposure to air pollution and percentage changes (25 h post-pre) in platelet counts (outdoor sites). (DOC) [file pone.0058944.s016.doc]

**Table S15** Two-pollutant models of associations between exposure to air pollution and percentage changes (25h post-pre) in platelet counts (outdoor sites).

|  | **IQR** | **A D J U S T M E N T P O L L U T A N T S** | | | | | | | | | | | | | | | | | | | | | | | | | |
| --- | --- | --- | --- | --- | --- | --- | --- | --- | --- | --- | --- | --- | --- | --- | --- | --- | --- | --- | --- | --- | --- | --- | --- | --- | --- | --- | --- |
|  |  | **PM_10_** | **PM_2.5_** | **PM_2.5_**-**_10_** | **PNC** | **Abs.^a^** | **EC (F)** | **EC (C)** | **OC (F)** | **OC (C)** | **Fe (tot)** | **Fe (sol)** | **Cu (tot)** | **Cu (sol)** | **Ni (tot)** | **Ni (sol)** | **V (tot)** | **V (sol)** | **End.** | **NO_3_^- a^** | **SO_4_^2- a^** | **OP^AA^** | **OP^GSH^** | **OP^TOTAL^** | **O_3_** | **NO_2_** | **NO_X_** |
| **PM_10_** | 13.50 | 0.68** | 0.66 | 0.69 | 0.65** | 0.73** | 0.67** | 0.72** | 0.89** | 0.30 | 0.73** | 0.73** | 0.73** | 0.73** | 0.75** | 0.76** | 0.71** | 0.72** | 0.67** | 0.18 | 0.66** | 0.97* | 0.57 | 1.02* | 0.82** | 0.98** | 0.75** |
| **PM_2.5_** | 11.54 | 0.03 | 0.72** | 0.59 | 0.67* | 0.75** | 0.70** | 0.76** | 1.13** | 0.34 | 0.78** | 0.79** | 0.79** | 0.78** | 0.79** | 0.83** | 0.75** | 0.77** | 0.70* | 0.00 | 0.69* | 1.31** | 0.63 | 1.17** | 0.83** | 1.02** | 0.76** |
| **PM_2.5_**-**_10_** | 8.23 | -0.02 | 0.40 | 1.06 | 1.10* | 1.22* | 1.15* | 1.19* | 1.05 | -0.17 | 1.17* | 1.03 | 1.13* | 1.11* | 1.19* | 1.08* | 1.06* | 1.09* | 1.01 | 0.38 | 1.12* | 0.31 | 0.55 | 0.37 | 1.37** | 1.45** | 1.33** |
| **PNC** | 32,906 | -0.78 | -0.72 | -1.02 | -0.96 | -0.64 | -1.59 | -1.11 | -0.96 | -0.51 | -5.54** | -0.83 | -5.69** | -3.38** | -1.07 | -0.84 | -0.84 | -0.88 | -0.89 | -0.44 | -0.88 | -0.57 | -0.79 | -0.67 | -0.76 | -0.49 | -0.14 |
| **Absorbance^a^** | 3.49 | -1.34 | -1.25 | -1.42 | -0.43 | -1.13 | -2.15 | -0.99 | -1.16 | -0.68 | -3.09 | -0.65 | -4.47** | -5.41** | -1.12 | -0.90 | -0.98 | -1.05 | -1.04 | -0.97 | -1.07 | -1.35 | -1.45 | -1.51 | -0.86 | -0.11 | 0.76 |
| **EC (F)** | 4.35 | -1.16 | -1.05 | -1.44 | 0.96 | 1.29 | -1.23 | -1.01 | -1.22 | -0.54 | -6.02* | -0.58 | -8.96** | -6.74** | -1.24 | -0.94 | -0.96 | -1.02 | -1.10 | -0.73 | -1.14 | -1.19 | -1.41 | -1.32 | -0.86 | -0.13 | 0.98 |
| **EC (C)** | 0.40 | -0.87 | -0.74 | -1.21 | 0.35 | 0.01 | -0.08 | -0.99 | -0.96 | -0.61 | -2.23 | -0.42 | -3.90* | -3.99** | -1.33 | -0.83 | -0.74 | -0.84 | -0.98 | -0.76 | -1.25 | -1.12 | -1.37 | -1.33 | -0.69 | 0.00 | 1.28 |
| **OC (F)** | 1.82 | -0.79 | -1.18 | 0.05 | 0.01 | 0.20 | 0.07 | 0.06 | 0.10 | 0.05 | 0.18 | -0.07 | 0.23 | 0.21 | 0.23 | -0.08 | 0.00 | 0.01 | 0.09 | -0.70 | 0.07 | 0.05 | 0.28 | 0.16 | 0.06 | 0.40 | 0.11 |
| **OC (C)** | 0.79 | 0.68 | 0.71 | 1.10* | 0.95** | 0.97** | 0.97** | 0.98** | 1.01** | 1.01** | 1.01** | 1.00** | 1.02** | 1.02** | 1.14** | 1.00** | 0.98** | 0.99** | 1.16** | 0.60 | 0.99** | 0.78 | 1.06** | 0.93 | 1.04** | 1.03** | 0.95** |
| **Fe (tot)** | 895.10 | -0.11 | 0.00 | -0.39 | 4.35** | 1.71 | 3.27* | 0.89 | -0.16 | -0.05 | -0.20 | 0.75 | -2.80 | -1.25 | -0.24 | -0.05 | -0.14 | -0.17 | -0.12 | 0.26 | -0.13 | -0.15 | -0.27 | -0.25 | -0.03 | 0.23 | 0.65 |
| **Fe (sol)** | 32.09 | -0.01 | 0.03 | -0.56 | -0.12 | -0.49 | -0.56 | -0.68 | -0.97 | -0.08 | -1.74 | -0.93 | -2.92* | -2.49* | -1.03 | -0.84 | -0.83 | -0.87 | -0.97 | 0.34 | -0.81 | 0.03 | -0.40 | -0.15 | -0.77 | -0.63 | -0.26 |
| **Cu (tot)** | 57.96 | 0.14 | 0.26 | -0.15 | 6.86** | 4.22** | 7.95** | 3.00 | 0.12 | 0.25 | 4.13 | 2.58 | 0.04 | -1.23 | 0.01 | 0.38 | 0.16 | 0.11 | 0.27 | 0.59 | 0.15 | 0.26 | 0.18 | 0.13 | 0.44 | 0.96 | 1.93 |
| **Cu (sol)** | 8.65 | 0.11 | 0.15 | 0.12 | 3.45** | 4.86** | 5.26** | 2.90* | 0.29 | 0.41 | 1.68 | 2.02 | 1.29 | 0.29 | 0.29 | 0.50 | 0.30 | 0.27 | 0.44 | 0.40 | 0.28 | 0.01 | 0.03 | -0.11 | 0.74 | 1.64 | 2.47** |
| **Ni (tot)** | 3.53 | -0.09 | -0.07 | -0.12 | -0.13 | -0.11 | -0.11 | -0.15 | -0.03 | -0.23 | -0.04 | -0.09 | -0.02 | 0.00 | -0.02 | -0.04 | -0.05 | -0.03 | -0.04 | 0.02 | 0.03 | -0.05 | -0.07 | -0.04 | -0.08 | -0.06 | -0.10 |
| **Ni (sol)** | 1.82 | 0.19 | 0.30 | -0.41 | -0.21 | -0.46 | -0.33 | -0.29 | -0.69 | -0.12 | -0.59 | -0.17 | -0.77 | -0.77 | -0.63 | -0.62 | -0.22 | -0.35 | -0.56 | 0.35 | -0.54 | -0.10 | -0.44 | -0.25 | -0.56 | -0.52 | -0.21 |
| **V (tot) ^b^** | 2.04 | -0.21 | -0.23 | -0.27 | -0.33 | -0.40 | -0.34 | -0.28 | -0.40 | -0.25 | -0.39 | -0.35 | -0.41 | -0.40 | -0.41 | -0.33 | -0.40 | -1.31 | -0.38 | -0.37 | -0.68 | -0.14 | -0.10 | -0.16 | -0.33 | -0.29 | -0.24 |
| **V (sol) ^b^** | 1.94 | -0.10 | -0.08 | -0.27 | -0.34 | -0.44 | -0.34 | -0.26 | -0.38 | -0.23 | -0.38 | -0.33 | -0.39 | -0.38 | -0.39 | -0.23 | 1.17 | -0.39 | -0.36 | -0.22 | -0.54 | -0.06 | -0.16 | -0.12 | -0.34 | -0.31 | -0.24 |
| **Endotoxin** | 0.19 | 0.00 | 0.00 | 0.01 | 0.00 | 0.00 | 0.00 | 0.00 | 0.01 | -0.01 | 0.00 | 0.00 | 0.01 | 0.01 | 0.01 | 0.00 | 0.00 | 0.00 | 0.01 | 0.00 | 0.01 | 0.01 | 0.01 | 0.01 | 0.01 | 0.00 | 0.00 |
| **NO_3_^- a^** | 5.19 | 0.65 | 0.83 | 0.72* | 0.76* | 0.80** | 0.78** | 0.87** | 1.02** | 0.55 | 0.93** | 0.96** | 0.94** | 0.90** | 0.89** | 0.94** | 0.88** | 0.87** | 0.81** | 0.83** | 0.94** | 1.18* | 0.58 | 0.93 | 1.06** | 1.06** | 0.84** |
| **SO_4_^2- a^** | 2.99 | 0.21 | 0.14 | 0.46 | 0.30 | 0.34 | 0.34 | 0.50 | 0.38 | 0.37 | 0.40 | 0.36 | 0.42 | 0.41 | 0.42 | 0.39 | 0.65 | 0.50 | 0.40 | -0.23 | 0.38 | 0.02 | 0.09 | 0.04 | 0.58 | 0.47 | 0.39 |
| **OP^AA^** | 19.08 | -0.93 | -1.27 | 0.15 | 0.28 | 0.44 | 0.32 | 0.56 | 0.24 | -0.01 | 0.50 | 0.51 | 0.51 | 0.50 | 0.49 | 0.49 | 0.51 | 0.50 | 0.25 | -0.96 | 0.26 | 0.26 | 0.30 | 0.47 | 0.46 | 0.80 | 0.50 |
| **OP^GSH^** | 15.53 | -0.61 | -0.53 | -0.25 | 0.48 | 0.62 | 0.50 | 0.65 | 0.10 | -0.91 | 0.33 | 0.24 | 0.15 | 0.19 | 0.16 | 0.25 | 0.22 | 0.22 | -0.11 | -0.27 | 0.08 | -0.14 | 0.09 | -0.38 | 0.33 | 0.46 | 0.50 |
| **OP^TOTAL^** | 38.71 | -1.59 | -1.70 | 0.10 | 0.49 | 0.78 | 0.56 | 0.94 | 0.25 | -0.43 | 0.72 | 0.65 | 0.67 | 0.71 | 0.66 | 0.64 | 0.70 | 0.68 | 0.28 | -1.01 | 0.33 | -0.35 | 0.61 | 0.35 | 0.69 | 1.17 | 0.79 |
| **O_3_** | 9.74 | 1.94 | 1.77 | 1.91 | 0.73 | 0.55 | 0.74 | 0.48 | 1.19 | 1.13 | 0.91 | 0.66 | 1.14 | 1.35 | 1.05 | 0.87 | 0.75 | 0.85 | 1.06 | 2.26* | 1.68 | 1.38 | 1.15 | 1.41 | 1.20 | -0.56 | -1.07 |
| **NO_2_** | 10.54 | -2.43** | -2.35** | -2.02** | -1.13 | -1.35 | -1.36 | -1.21 | -1.60 | -1.29 | -1.35 | -1.03 | -1.68 | -2.28* | -1.24 | -1.16 | -1.09 | -1.16 | -1.37 | -2.17** | -1.55 | -2.07* | -1.38 | -2.01* | -1.80 | -1.43 | 1.39 |
| **NO_X_** | 28.05 | -1.66** | -1.57** | -1.77** | -1.37 | -1.99 | -2.00 | -1.97 | -1.46* | -1.09 | -1.73* | -1.17 | -2.29** | -2.78** | -1.34* | -1.23 | -1.19 | -1.23 | -1.47* | -1.49* | -1.46* | -1.58* | -1.50* | -1.63* | -2.05 | -2.54 | -1.46* |

For explanation see Table S9.
