# Supplementary material for: Composition of PM Affects Acute Vascular Inflammatory and Coagulative Markers - The RAPTES Project
Source: PLoS One. 2013 Mar 13;8(3):e58944. doi: 10.1371/journal.pone.0058944 (PMC3596332; doi:10.1371/journal.pone.0058944)
Supplement: Table S16 — Two-pollutant models of associations between exposure to air pollution and percentage changes (25 h post-pre) in von Willebrand Factor (outdoor sites). (DOC) [file pone.0058944.s017.doc]

**Table S16** Two-pollutant models of associations between exposure to air pollution and percentage changes (25h post-pre) in von Willebrand Factor (outdoor sites).

|  | **IQR** | **A D J U S T M E N T P O L L U T A N T S** | | | | | | | | | | | | | | | | | | | | | | | | | |
| --- | --- | --- | --- | --- | --- | --- | --- | --- | --- | --- | --- | --- | --- | --- | --- | --- | --- | --- | --- | --- | --- | --- | --- | --- | --- | --- | --- |
|  |  | **PM_10_** | **PM_2.5_** | **PM_2.5-10_** | **PNC** | **Abs.^a^** | **EC (F)** | **EC (C)** | **OC (F)** | **OC (C)** | **Fe (tot)** | **Fe (sol)** | **Cu (tot)** | **Cu (sol)** | **Ni (tot)** | **Ni (sol)** | **V (tot)** | **V (sol)** | **End.** | **NO_3_^- a^** | **SO_4_^2- a^** | **OP^AA^** | **OP^GSH^** | **OP^TOTAL^** | **O_3_** | **NO_2_** | **NO_X_** |
| **PM_10_** | 13.50 | 0.84 | -5.32** | 2.77** | 0.58 | 0.69 | 0.67 | 0.87 | 0.11 | 2.54** | 0.60 | 0.93 | 0.60 | 0.83 | 0.90 | 0.72 | 0.51 | 0.46 | 0.77 | -0.69 | 0.41 | 0.90 | 0.64 | 1.33 | 0.89 | 0.72 | 0.84 |
| **PM_2.5_** | 11.54 | 7.26** | 1.28** | 2.36** | 1.31** | 1.27** | 1.30** | 1.32** | 0.43 | 2.92** | 0.88 | 1.51** | 1.35** | 1.30** | 1.35** | 1.20* | 0.86 | 0.79 | 1.23* | 2.34 | 0.79 | 1.70 | 0.72 | 1.93* | 1.33** | 1.20* | 1.28** |
| **PM_2.5_**-**_10_** | 8.23 | -4.87** | -3.28** | -0.31 | -0.24 | -0.30 | -0.30 | -0.19 | -0.77 | -1.77 | -0.34 | -0.39 | -0.34 | -0.48 | -0.37 | -0.77 | -0.87 | -0.85 | -0.47 | -2.29 | -0.96 | -1.03 | 0.52 | -1.04 | -0.28 | -0.54 | -0.29 |
| **PNC** | 32,906 | 0.77 | 0.35 | 0.64 | 0.61 | -1.80 | -1.14 | 0.83 | 0.30 | 1.08 | 1.61 | 2.43 | 0.30 | -1.24 | 0.90 | 1.88 | 0.29 | 0.26 | 1.13 | 1.05 | -0.08 | -1.68 | -1.05 | -2.05 | 0.73 | 0.22 | 0.62 |
| **Absorbance^a^** | 3.49 | -0.36 | 0.70 | -0.04 | 3.28 | -0.14 | 5.09 | 4.09 | 0.31 | 0.47 | 4.29 | 3.27 | 2.19 | -2.28 | 0.48 | 0.64 | 1.05 | 0.88 | 0.56 | 1.12 | 0.53 | -2.23 | -1.02 | -2.48 | 2.00 | 0.96 | 0.14 |
| **EC (F)** | 4.35 | -0.51 | 0.63 | 1.18 | 2.71 | -6.11 | 1.10 | 4.02 | 0.43 | 1.83 | 4.24 | 3.22 | 2.29 | -1.86 | -0.03 | 2.74 | 0.78 | 0.71 | 2.20 | 1.01 | 0.17 | -2.38 | -1.32 | -2.73 | 1.49 | 0.38 | 1.71 |
| **EC (C)** | 0.40 | -0.64 | -0.26 | 0.25 | -2.06 | -4.98 | -4.50 | 0.17 | 0.82 | 0.28 | -2.41 | 0.02 | -3.53 | -7.43** | -0.09 | 1.43 | 0.00 | -0.03 | 0.42 | 0.34 | -0.66 | -2.30 | -2.42 | -2.65 | -0.09 | -1.70 | -1.32 |
| **OC (F)** | 1.82 | 2.99** | 2.60* | 3.13** | 3.14** | 3.08** | 3.11** | 3.51** | 3.10** | 3.40** | 3.45** | 3.82** | 3.57** | 3.37** | 3.59** | 3.84** | 3.50** | 3.51** | 3.08** | 2.87** | 3.12** | 2.73 | 2.73** | 2.65* | 3.11** | 3.02** | 3.10** |
| **OC (C)** | 0.79 | -2.46* | -2.38** | 1.16 | 0.36 | 0.33 | 0.38 | 0.24 | -0.13 | 0.30 | 0.28 | 0.24 | 0.28 | 0.34 | 0.29 | 0.21 | -0.13 | -0.12 | 0.21 | -1.03 | -0.17 | 0.62 | 1.25 | 0.81 | 0.30 | 0.28 | 0.31 |
| **Fe (tot)** | 895.10 | 0.70 | 0.87 | 0.73 | -0.62 | -1.87 | -1.59 | 1.82 | 0.39 | 0.71 | 0.66 | 1.49 | -1.59 | -1.41 | 0.63 | 1.31 | -0.10 | -0.12 | 0.96 | 0.99 | -0.08 | -0.93 | -1.09 | -1.09 | 0.66 | 0.27 | 0.52 |
| **Fe (sol)** | 32.09 | 0.41 | 1.12 | -0.64 | -2.15 | -2.74 | -1.75 | 0.19 | 1.53 | -0.38 | -1.45 | -0.58 | -2.30 | -4.20* | -0.55 | 0.09 | 0.16 | 0.25 | -0.37 | 1.06 | 0.13 | -0.57 | -1.18 | -0.87 | -0.62 | -1.13 | -0.73 |
| **Cu (tot)** | 57.96 | 1.20 | 0.56 | 1.26 | 0.82 | -2.05 | -0.64 | 3.80 | 1.26 | 1.24 | 3.50 | 3.21 | 1.17 | -2.20 | 1.18 | 2.49 | 0.53 | 0.55 | 1.63 | 1.58 | 0.38 | -1.08 | -1.26 | -1.31 | 1.23 | 0.58 | 1.09 |
| **Cu (sol)** | 8.65 | 1.32 | 1.38 | 1.02 | 2.86 | 2.79 | 2.98 | 6.61** | 1.45 | 0.96 | 3.28 | 4.61* | 3.47 | 0.90 | 1.04 | 2.51 | 1.41 | 1.40 | 1.10 | 1.82 | 1.35 | -0.08 | 0.22 | -0.24 | 1.04 | 0.46 | 1.50 |
| **Ni (tot)** | 3.53 | -0.15 | -0.15 | 0.08 | -0.01 | 0.10 | 0.07 | -0.11 | -0.33 | 0.02 | -0.03 | 0.03 | 0.01 | 0.14 | 0.07 | -0.16 | -0.18 | -0.15 | 0.05 | 0.12 | -0.06 | 0.73 | 0.67 | 0.75 | 0.08 | 0.09 | 0.07 |
| **Ni (sol)** | 1.82 | -1.40 | -0.90 | -2.07 | -2.42 | -1.17 | -2.29 | -1.99 | 1.07 | -0.93 | -2.17 | -1.08 | -2.52 | -2.91* | -2.11 | -2.00 | -0.36 | 0.44 | -1.95 | -1.23 | -1.37 | -0.42 | -0.93 | -0.70 | -2.03 | -2.20 | -2.32 |
| **V (tot) ^b^** | 2.04 | -1.16 | -1.08 | -1.38 | -1.31 | -1.25 | -1.32 | -1.30 | 0.24 | -1.33 | -1.30 | -1.31 | -1.32 | -1.24 | -1.37 | -1.18 | -1.30 | 1.50 | -1.29 | -1.26 | -0.72 | -1.53 | -1.34 | -1.57 | -1.34 | -1.41 | -1.35 |
| **V (sol) ^b^** | 1.94 | -1.51 | -1.35 | -1.77 | -1.71 | -1.62 | -1.71 | -1.70 | 0.25 | -1.73 | -1.70 | -1.72 | -1.72 | -1.63 | -1.74 | -1.91 | -3.35 | -1.70 | -1.68 | -1.56 | -1.05 | -1.16 | -1.30 | -1.30 | -1.71 | -1.73 | -1.75 |
| **Endotoxin** | 0.19 | 0.01 | 0.00 | 0.02 | 0.02 | 0.02 | 0.02 | 0.01 | 0.01 | 0.01 | 0.01 | 0.01 | 0.01 | 0.01 | 0.01 | 0.00 | 0.00 | 0.00 | 0.02 | 0.01 | 0.01 | 0.02 | 0.02 | 0.02 | 0.02 | 0.02 | 0.02 |
| **NO_3_^- a^** | 5.19 | 1.58 | -1.20 | 1.72** | 0.63 | 1.06 | 1.09 | 0.53 | 0.28 | 1.66* | 0.67 | 1.26 | 0.66 | 1.13* | 0.91 | 0.86 | 0.70 | 0.60 | 0.94 | 1.01 | 0.78 | 0.96 | 0.39 | 0.34 | 1.09 | 0.90 | 1.01 |
| **SO_4_^2- a^** | 2.99 | 0.65 | 0.42 | 0.85 | 0.83 | 0.88 | 0.85 | 0.91 | -0.09 | 0.92 | 0.88 | 0.92 | 0.95 | 1.00 | 0.87 | 0.76 | 1.09 | 1.01 | 0.81 | 0.21 | 0.84 | 0.65 | 0.79 | 0.72 | 0.85 | 0.79 | 0.84 |
| **OP^AA^** | 19.08 | 0.14 | -0.75 | 1.69 | 1.34 | 1.60* | 1.42 | 1.12 | -0.03 | 0.53 | 0.94 | 0.82 | 0.94 | 0.94 | 1.16 | 0.87 | 1.05 | 0.91 | 1.24 | 0.25 | 0.29 | 1.28 | 1.58 | 2.85 | 1.78* | 1.68 | 1.56 |
| **OP^GSH^** | 15.53 | -0.11 | -0.04 | 0.36 | 1.19 | 1.04 | 1.06 | 0.92 | 0.34 | -1.16 | 0.61 | 0.23 | 0.48 | 0.00 | 0.60 | 0.26 | 0.35 | 0.30 | -0.04 | 0.43 | -0.32 | -1.01 | 0.68 | -2.26 | 1.12 | 0.79 | 0.93 |
| **OP^TOTAL^** | 38.71 | -1.01 | -1.83 | 2.30 | 2.02 | 2.31 | 2.05 | 1.77 | 0.14 | 0.22 | 1.37 | 0.98 | 1.33 | 1.21 | 1.66 | 1.07 | 1.42 | 1.20 | 1.28 | 0.30 | 0.21 | -2.49 | 3.23 | 1.56 | 2.35 | 1.97 | 2.03 |
| **O_3_** | 9.74 | 0.66 | 0.75 | 0.14 | 0.42 | 1.51 | 0.76 | -0.42 | -0.25 | 0.00 | 0.01 | -0.22 | 0.20 | 0.50 | -0.17 | -0.59 | -0.67 | -0.36 | -0.07 | 0.86 | 0.08 | 3.14 | 1.99 | 3.00 | 0.30 | 4.42 | 0.20 |
| **NO_2_** | 10.54 | 0.98 | 0.66 | 1.00 | 0.99 | 0.38 | 0.91 | 2.45 | 0.37 | 1.10 | 1.33 | 2.35 | 1.22 | 0.84 | 1.17 | 2.29 | 1.71 | 1.51 | 2.25 | 1.16 | 0.90 | -1.39 | -0.38 | -0.94 | 4.70 | 1.73 | 12.29** |
| **NO_X_** | 28.05 | 0.09 | 0.14 | -0.11 | 0.00 | -0.29 | -0.56 | 1.40 | 0.13 | 0.12 | 0.31 | 0.33 | 0.11 | -0.83 | 0.12 | 1.11 | 0.67 | 0.62 | 0.26 | 0.34 | 0.06 | -1.53 | -0.86 | -1.49 | -0.09 | -8.49** | -0.20 |

For explanation see Table S9.
