# Supplementary material for: Composition of PM Affects Acute Vascular Inflammatory and Coagulative Markers - The RAPTES Project
Source: PLoS One. 2013 Mar 13;8(3):e58944. doi: 10.1371/journal.pone.0058944 (PMC3596332; doi:10.1371/journal.pone.0058944)
Supplement: Table S17 — Two-pollutant models of associations between exposure to air pollution and percentage changes (25 h post-pre) in tPA/PAI-1 complex (all sites). (DOC) [file pone.0058944.s018.doc]

**Table S17** Two-pollutant models of associations between exposure to air pollution and percentage changes (25h post-pre) in tPA/PAI-1 complex (all sites).

|  | **IQR** | **A D J U S T M E N T P O L L U T A N T S** | | | | | | | | | | | | | | | | | | | | | | | | | |
| --- | --- | --- | --- | --- | --- | --- | --- | --- | --- | --- | --- | --- | --- | --- | --- | --- | --- | --- | --- | --- | --- | --- | --- | --- | --- | --- | --- |
|  |  | **PM_10_** | **PM_2.5_** | **PM_2.5-10_** | **PNC** | **Abs.^a^** | **EC (F)** | **EC (C)** | **OC (F)** | **OC (C)** | **Fe (tot)** | **Fe (sol)** | **Cu (tot)** | **Cu (sol)** | **Ni (tot)** | **Ni (sol)** | **V (tot)** | **V (sol)** | **End.** | **NO_3_^- a^** | **SO_4_^2- a^** | **OP^AA^** | **OP^GSH^** | **OP^TOTAL^** | **O_3_** | **NO_2_** | **NO_X_** |
| **PM_10_** | 13.50 | 1.09* | 1.62 | 0.15 | 0.97 | -0.42 | 0.29 | 1.16 | 1.04 | 0.91 | 2.97 | 1.44** | 2.54 | 1.06 | 2.30 | 1.28** | 1.44 | 1.11* | 1.05* | 1.07* | 1.16* | 2.33 | 1.73 | 2.38 | -1.48 | 0.88 | 0.73 |
| **PM_2.5_** | 11.54 | -1.24 | 2.36* | 0.13 | 2.17 | -0.70 | 0.44 | 0.80 | 2.29 | 1.72 | 1.46 | 3.04* | 2.26 | 2.06 | 2.71 | 2.59* | 2.25 | 2.31 | 2.26 | 2.36 | 2.41* | 3.97 | 2.16 | 3.10 | -2.60 | 1.77 | 1.48 |
| **PM_2.5_**-**_10_** | 8.23 | 0.89 | 0.98 | 1.03* | 0.91 | -0.37 | 0.29 | 1.19 | 0.96 | 0.87 | 4.34 | 1.35** | 2.41 | 1.02 | 2.35 | 1.26** | 1.50 | 1.08* | 1.00* | 1.03* | 1.14** | 1.94 | 1.87 | 2.21 | -1.35 | 0.86 | 0.71 |
| **PNC** | 32,906 | 12.67 | 13.48 | 12.33 | 14.90 | 6.29 | 7.85 | 11.31 | 14.49 | 15.25 | 11.93 | 15.03 | 12.24 | 12.95 | 13.80 | 18.26* | 12.70 | 15.13 | 14.62 | 18.09* | 17.55* | 12.89 | 12.46 | 12.65 | 6.78 | 7.82 | 6.39 |
| **Absorbance^a^** | 3.49 | 12.75 | 11.81 | 12.59 | 8.04 | 9.73** | 72.35* | 14.28 | 9.52* | 8.17 | 17.08 | 13.07** | 16.82 | 9.48 | 13.38 | 11.39** | 12.47* | 9.48** | 10.04** | 9.93** | 10.95** | 18.73* | 15.45 | 18.30* | -6.46 | 7.33 | 6.87 |
| **EC (F)** | 4.35 | 7.49 | 8.40 | 7.33 | 7.49 | -40.29 | 9.82* | 8.40 | 8.89 | 7.63 | 11.78 | 12.35** | 12.47 | 8.57 | 11.51 | 11.83** | 11.40 | 9.82* | 9.94* | 10.33** | 11.40** | 15.10 | 10.96 | 13.48 | -8.76 | 7.07 | 5.84 |
| **EC (C)** | 0.40 | -0.07 | 1.02 | -0.21 | 1.24 | -0.87 | 0.23 | 1.47* | 1.41 | 1.08 | 1.86 | 2.37** | 3.73 | 1.59 | 2.14 | 1.78** | 1.63 | 1.47* | 1.48* | 1.46* | 1.57* | 3.71 | 2.25 | 3.75 | -1.61 | 1.23 | 0.93 |
| **OC (F)** | 1.82 | 0.60 | 0.32 | 1.14 | 5.04 | 0.40 | 1.88 | 0.52 | 5.33 | 2.84 | 1.47 | 6.63 | 1.45 | 1.58 | 2.33 | 5.28 | 3.27 | 4.53 | 4.98 | 5.26 | 5.04 | 1.35 | -0.05 | 0.48 | -0.93 | 2.99 | 3.09 |
| **OC (C)** | 0.79 | 1.40 | 2.03 | 1.47 | 5.16 | 1.74 | 2.39 | 2.34 | 4.14 | 4.93 | 2.51 | 5.10 | 2.85 | 3.69 | 3.32 | 5.17 | 3.63 | 4.85 | 5.12 | 5.21 | 5.14 | 3.84 | 2.99 | 3.41 | 0.46 | 4.32 | 3.68 |
| **Fe (tot)** | 895.10 | -0.30 | 0.07 | -0.55 | 0.14 | -0.16 | -0.04 | -0.05 | 0.14 | 0.11 | 0.16* | 0.21* | 0.36 | 0.14 | 0.28 | 0.20** | 0.21 | 0.17* | 0.16 | 0.16* | 0.18* | 0.30 | 0.15 | 0.25 | -0.25 | 0.14 | 0.10 |
| **Fe (sol)** | 32.09 | -2.85 | -2.48 | -2.76 | -0.43 | -3.66 | -2.65 | -4.52 | -1.44 | -0.52 | -2.68 | 0.87 | -3.65 | -6.75 | -1.65 | 1.68 | -0.85 | 0.75 | 0.39 | 0.87 | 0.96 | -4.29 | -3.92 | -4.39 | -3.56 | 0.27 | -1.23 |
| **Cu (tot)** | 57.96 | -0.32 | 0.01 | -0.32 | 0.18 | -0.22 | -0.07 | -0.36 | 0.18 | 0.13 | -0.27 | 0.32* | 0.21 | 0.18 | 0.23 | 0.27* | 0.20 | 0.21 | 0.21 | 0.21 | 0.23* | 0.39 | 0.13 | 0.28 | -0.26 | 0.17 | 0.12 |
| **Cu (sol)** | 8.65 | 0.02 | 0.06 | 0.02 | 0.18 | 0.01 | 0.07 | -0.03 | 0.18 | 0.11 | 0.06 | 0.54* | 0.05 | 0.22 | 0.11 | 0.27 | 0.14 | 0.22 | 0.21 | 0.21 | 0.21 | 0.12 | 0.02 | 0.07 | -0.05 | 0.19 | 0.11 |
| **Ni (tot)** | 3.53 | -1.79 | -0.23 | -2.05 | 1.18 | -0.81 | -0.34 | -0.76 | 1.02 | 0.62 | -1.09 | 1.50 | -0.16 | 0.86 | 1.25 | 1.48 | 0.83 | 1.26 | 1.20 | 1.27 | 1.49* | 0.76 | -0.22 | 0.17 | -1.34 | 1.08 | 0.78 |
| **Ni (sol)** | 1.82 | -7.94 | -6.75 | -8.58 | -8.21 | -8.90 | -8.77 | -8.52 | -5.25 | -5.93 | -8.68 | -6.17 | -8.82 | -7.52 | -7.43 | -5.21 | -14.79** | -3.23 | -6.14 | -4.86 | -4.54 | -5.99 | -7.62 | -6.79 | -10.17 | -6.41 | -7.04 |
| **V (tot) ^b^** | 2.04 | -0.82 | 0.15 | -1.14 | 1.48 | -0.96 | -0.49 | -0.30 | 1.41 | 1.03 | -0.69 | 1.91 | 0.12 | 1.21 | 0.75 | 3.69** | 1.76 | 2.53* | 1.71 | 1.83 | 1.94 | 1.70 | 0.99 | 1.35 | -3.12 | 1.33 | 1.11 |
| **V (sol) ^b^** | 1.94 | -4.98 | -4.32 | -5.41 | -5.45 | -4.48 | -5.10 | -4.92 | -3.45 | -4.66 | -5.33 | -4.88 | -5.16 | -4.77 | -5.02 | -3.04 | -9.06 | -4.94 | -5.55 | -4.69 | -5.43 | -6.02 | -7.01 | -6.44 | -6.81 | -6.58 | -5.81 |
| **Endotoxin** | 0.19 | -0.04 | -0.05 | -0.03 | -0.01 | 0.02 | 0.01 | 0.00 | -0.06 | -0.09 | 0.00 | -0.06 | -0.01 | -0.03 | -0.03 | -0.09 | -0.02 | -0.09 | -0.08 | -0.09 | -0.08 | -0.06 | -0.05 | -0.05 | 0.03 | -0.02 | 0.01 |
| **NO_3_^- a^** | 5.19 | 1.12 | -0.07 | 1.89 | 4.36 | 2.55 | 3.02 | 1.76 | 0.16 | -0.88 | 2.05 | 1.78 | 1.80 | 0.96 | 2.14 | 0.94 | 2.38 | 1.33 | 2.40 | 1.90 | 0.14 | 3.98 | 3.95 | 3.95 | 0.88 | -0.08 | 1.49 |
| **SO_4_^2- a^** | 2.99 | 5.00 | 4.16 | 5.49 | 5.95 | 6.28 | 6.38 | 4.88 | 3.24 | 4.33 | 5.38 | 3.74 | 5.00 | 3.48 | 5.79 | 3.00 | 4.80 | 4.15 | 3.80 | 3.64 | 3.75 | 7.11 | 7.17 | 7.16 | 3.97 | 2.78 | 4.12 |
| **OP^AA^** | 19.08 | -0.48 | -0.29 | -0.40 | 0.18 | -0.43 | -0.26 | -0.60 | 0.17 | 0.07 | -0.35 | 0.43 | -0.33 | 0.11 | 0.06 | 0.24 | 0.02 | 0.20 | 0.18 | 0.19 | 0.23 | 0.20 | -0.31 | -0.85 | -0.52 | 0.17 | 0.10 |
| **OP^GSH^** | 15.53 | -0.22 | -0.01 | -0.28 | 0.18 | -0.23 | -0.07 | -0.19 | 0.20 | 0.11 | -0.01 | 0.35 | 0.07 | 0.19 | 0.24 | 0.26 | 0.11 | 0.21 | 0.19 | 0.19 | 0.22 | 0.44 | 0.20 | 0.70 | -0.36 | 0.17 | 0.13 |
| **OP^TOTAL^** | 38.71 | -0.50 | -0.16 | -0.48 | 0.21 | -0.41 | -0.19 | -0.60 | 0.22 | 0.11 | -0.25 | 0.47 | -0.16 | 0.18 | 0.21 | 0.29 | 0.08 | 0.24 | 0.21 | 0.22 | 0.26 | 1.10 | -0.64 | 0.24 | -0.54 | 0.20 | 0.13 |
| **O_3_** | 9.74 | -25.29** | -22.66** | -24.94** | -13.78** | -21.69* | -22.81** | -22.87** | -15.57** | -14.54** | -25.46** | -17.81** | -22.81** | -15.84** | -20.72** | -17.17** | -24.51** | -15.61** | -15.46** | -14.97** | -15.12** | -25.13** | -23.91** | -25.45** | -15.08** | -13.48** | -14.13** |
| **NO_2_** | 10.54 | 16.76 | 16.54 | 17.19 | 15.37 | 12.55 | 14.43 | 17.07 | 18.30 | 18.41* | 17.36 | 20.07* | 17.71 | 18.71* | 18.44* | 21.50* | 17.67 | 22.13* | 20.01* | 20.55* | 19.79* | 19.29 | 18.82 | 19.02 | 5.96 | 20.50* | 9.63 |
| **NO_X_** | 28.05 | 12.16 | 12.67 | 12.19 | 12.86 | 7.81 | 10.26 | 11.69 | 15.09* | 13.91 | 12.40 | 17.93* | 12.76 | 13.96 | 13.64 | 18.13** | 13.96 | 17.22* | 17.13* | 16.58* | 17.02* | 13.81 | 12.86 | 13.26 | 2.39 | 9.69 | 16.76* |

For explanation see Table S9.
