# Supplementary material for: Composition of PM Affects Acute Vascular Inflammatory and Coagulative Markers - The RAPTES Project
Source: PLoS One. 2013 Mar 13;8(3):e58944. doi: 10.1371/journal.pone.0058944 (PMC3596332; doi:10.1371/journal.pone.0058944)
Supplement: Table S18 — Two-pollutant models of associations between exposure to air pollution and percentage changes (25 h post-pre) in tPA/PAI-1 complex (outdoor sites). (DOC) [file pone.0058944.s019.doc]

**Table S18** Two-pollutant models of associations between exposure to air pollution and percentage changes (25h post-pre) in tPA/PAI-1 complex (outdoor sites).

|  | **IQR** | **A D J U S T M E N T P O L L U T A N T S** | | | | | | | | | | | | | | | | | | | | | | | | | |
| --- | --- | --- | --- | --- | --- | --- | --- | --- | --- | --- | --- | --- | --- | --- | --- | --- | --- | --- | --- | --- | --- | --- | --- | --- | --- | --- | --- |
|  |  | **PM_10_** | **PM_2.5_** | **PM_2.5-10_** | **PNC** | **Abs.^a^** | **EC (F)** | **EC (C)** | **OC (F)** | **OC (C)** | **Fe (tot)** | **Fe (sol)** | **Cu (tot)** | **Cu (sol)** | **Ni (tot)** | **Ni (sol)** | **V (tot)** | **V (sol)** | **End.** | **NO_3_^- a^** | **SO_4_^2- a^** | **OP^AA^** | **OP^GSH^** | **OP^TOTAL^** | **O_3_** | **NO_2_** | **NO_X_** |
| **PM_10_** | 13.50 | 2.06 | 34.77* | -6.21 | 2.56 | 1.53 | 2.15 | 2.06 | 3.16 | -2.68 | 2.07 | 1.94 | 1.97 | 1.79 | 2.11 | -0.75 | 1.48 | 0.92 | 2.48 | 4.59 | 1.44 | 0.03 | -0.24 | -3.24 | 0.06 | 0.65 | 1.61 |
| **PM_2.5_** | 11.54 | -26.65* | 0.34 | -5.34 | 1.14 | 0.03 | 0.70 | 0.67 | 1.10 | -5.78 | 0.65 | -0.27 | 0.34 | 0.02 | 0.22 | -3.63 | -0.40 | -1.16 | 0.66 | -4.01 | -0.73 | -6.87 | -2.18 | -8.44 | -1.44 | -1.31 | 0.05 |
| **PM_2.5_**-**_10_** | 8.23 | 24.72* | 19.94* | 12.82 | 12.12 | 10.89 | 11.81 | 11.41 | 12.89 | 19.78 | 11.75 | 13.06 | 12.52 | 12.77 | 14.07 | 11.81 | 12.34 | 12.02 | 13.52 | 16.15 | 13.51 | 18.98 | 14.23 | 17.63 | 8.29 | 11.07 | 11.48 |
| **PNC** | 32,906 | 10.85 | 10.59 | 8.88 | 10.11 | -10.26 | 12.23 | -2.61 | 10.13 | 11.89 | 6.42 | 37.03* | 31.76 | 22.15 | 9.19 | 23.97** | 9.80 | 9.72 | 10.69 | 12.64 | 13.15 | 9.74 | 3.92 | 7.80 | 2.28 | 6.58 | 6.55 |
| **Absorbance^a^** | 3.49 | 15.94 | 16.45 | 13.14 | 31.22 | 16.46 | 115.57* | 9.84 | 16.98 | 17.72 | 25.04 | 33.67* | 47.42 | 61.30* | 15.76 | 22.44* | 14.97 | 13.57 | 18.50 | 17.01 | 18.50 | 17.64 | 13.20 | 15.50 | 1.70 | 15.75 | 19.39 |
| **EC (F)** | 4.35 | 14.14 | 14.17 | 10.77 | -2.86 | -53.39 | 13.94 | -12.86 | 13.90 | 16.37 | 1.50 | 37.33 | 49.47 | 45.96 | 12.37 | 30.81* | 13.54 | 13.35 | 15.53 | 15.87 | 16.87 | 14.38 | 8.04 | 11.91 | -1.62 | 6.66 | 6.76 |
| **EC (C)** | 0.40 | 18.67 | 18.69 | 14.33 | 22.40 | 7.55 | 34.27 | 18.43 | 18.82 | 21.54 | 21.58 | 40.61* | 45.34 | 45.63 | 21.07 | 50.69** | 24.82 | 26.20 | 21.46 | 19.15 | 17.67 | 17.24 | 11.51 | 14.99 | -0.74 | 15.16 | 19.02 |
| **OC (F)** | 1.82 | -4.12 | -2.21 | -1.49 | 0.09 | -2.39 | -0.68 | 0.81 | -0.93 | -2.63 | -0.34 | -2.52 | -0.78 | -1.69 | -1.14 | -17.39* | -4.39 | -8.18 | -0.86 | -2.59 | -1.95 | -12.28 | -2.30 | -9.45 | -0.89 | -3.87 | -1.35 |
| **OC (C)** | 0.79 | 7.38 | 10.01 | -4.66 | 5.75 | 5.44 | 5.60 | 5.59 | 4.58 | 4.33 | 4.89 | 4.69 | 4.65 | 4.53 | 5.51 | 2.22 | 3.87 | 3.66 | 5.56 | 5.85 | 4.31 | 3.65 | 0.58 | 1.43 | 4.14 | 4.11 | 4.83 |
| **Fe (tot)** | 895.10 | 7.71 | 7.71 | 4.83 | 2.37 | -5.94 | 6.66 | -1.80 | 7.46 | 8.38 | 7.53 | 24.24* | 55.20 | 17.33 | 7.33 | 17.76* | 7.81 | 7.82 | 9.11 | 8.98 | 10.26 | 7.62 | 4.07 | 6.49 | 2.76 | 5.00 | 4.77 |
| **Fe (sol)** | 32.09 | 0.24 | -2.28 | 0.81 | -28.50 | -19.27 | -19.80 | -18.84 | -3.37 | 2.12 | -23.47 | -1.96 | -19.97 | -11.06 | -3.24 | 20.18 | -1.47 | -0.85 | -2.33 | -0.74 | 1.25 | -2.84 | -6.95 | -2.61 | -6.99 | -4.76 | -7.39 |
| **Cu (tot)** | 57.96 | 6.74 | 6.69 | 3.15 | -22.40 | -24.59 | -22.89 | -18.37 | 6.35 | 7.71 | -43.30 | 28.67 | 6.58 | 12.15 | 5.87 | 25.43* | 7.27 | 7.66 | 8.07 | 7.85 | 10.18 | 6.30 | 0.36 | 4.49 | -2.69 | 1.08 | -0.65 |
| **Cu (sol)** | 8.65 | 3.71 | 4.16 | 1.38 | -13.83 | -29.25 | -20.01 | -17.46 | 4.24 | 5.01 | -13.01 | 12.89 | -5.01 | 4.17 | 3.43 | 14.22 | 3.64 | 3.17 | 4.45 | 4.49 | 5.56 | 2.64 | -4.35 | 0.15 | -4.89 | -3.04 | -4.25 |
| **Ni (tot)** | 3.53 | -1.11 | -0.94 | -1.80 | -0.06 | 0.12 | -0.18 | 0.92 | -0.86 | -1.90 | -0.22 | -1.15 | -0.43 | -0.70 | -0.93 | -1.71 | -1.28 | -1.24 | -0.96 | -0.88 | -0.39 | -2.00 | -1.46 | -1.71 | 0.82 | -0.64 | -0.50 |
| **Ni (sol)** | 1.82 | -21.80** | -24.06** | -20.50** | -29.61** | -24.38** | -27.67** | -32.89** | -33.08** | -20.48** | -28.20** | -29.10** | -28.78** | -24.96** | -21.99** | -21.29** | -24.73** | -23.49* | -22.11** | -23.41** | -20.53** | -21.40* | -24.37** | -20.49* | -22.30** | -22.06** | -24.65** |
| **V (tot) ^b^** | 2.04 | -4.07 | -4.48 | -3.37 | -4.76 | -3.93 | -4.76 | -7.11 | -5.59 | -3.83 | -4.63 | -4.36 | -4.63 | -4.28 | -4.81 | 4.24 | -4.40 | 58.34** | -4.58 | -4.39 | -6.67 | -6.02 | -7.75 | -6.94 | -6.59 | -5.37 | -5.31 |
| **V (sol) ^b^** | 1.94 | -8.51 | -9.22 | -7.96 | -9.05 | -7.86 | -9.02 | -11.65 | -12.11 | -8.32 | -8.99 | -8.79 | -9.09 | -8.68 | -9.07 | 2.66 | -46.65** | -8.83 | -9.01 | -8.75 | -10.09 | -15.16 | -18.20 | -15.74 | -9.82 | -9.32 | -9.65 |
| **Endotoxin** | 0.19 | -0.05 | -0.04 | -0.06 | 0.02 | 0.05 | 0.03 | 0.07 | -0.03 | -0.07 | 0.06 | -0.01 | 0.04 | 0.01 | 0.01 | -0.06 | -0.02 | -0.03 | -0.03 | -0.04 | -0.03 | -0.04 | -0.14 | -0.07 | 0.03 | 0.01 | 0.02 |
| **NO_3_^- a^** | 5.19 | -3.15 | 5.29 | -3.10 | 3.27 | 1.92 | 2.43 | 1.73 | 1.99 | -2.07 | 2.55 | 0.91 | 1.79 | 1.26 | 0.93 | -2.84 | 1.03 | 0.47 | 1.52 | 1.28 | -1.19 | -0.51 | 1.10 | -2.27 | -1.55 | 0.05 | 1.28 |
| **SO_4_^2- a^** | 2.99 | 4.20 | 4.91 | 5.37 | 6.61 | 5.95 | 5.87 | 4.17 | 4.84 | 4.53 | 6.53 | 4.74 | 5.84 | 4.95 | 4.33 | 2.61 | 6.73 | 6.04 | 4.62 | 5.51 | 4.64 | 6.09 | 6.60 | 5.57 | 1.38 | 4.11 | 4.89 |
| **OP^AA^** | 19.08 | 4.73 | 14.10 | -2.13 | 4.46 | 2.44 | 4.01 | 2.77 | 11.81 | 1.55 | 4.05 | 3.49 | 4.02 | 3.82 | 3.50 | 0.07 | 4.48 | 3.55 | 4.89 | 5.34 | 2.67 | 4.77 | 1.02 | -13.43 | -0.04 | 1.78 | 3.42 |
| **OP^GSH^** | 15.53 | 14.59 | 16.77 | 4.86 | 12.12 | 9.37 | 11.75 | 9.69 | 14.19 | 12.94 | 11.53 | 14.44 | 13.50 | 15.75 | 12.63 | 18.75* | 15.04 | 16.67 | 19.88 | 13.51 | 13.69 | 13.39 | 14.27 | 12.46 | 8.42 | 11.84 | 12.42 |
| **OP^TOTAL^** | 38.71 | 18.25 | 29.69 | -0.95 | 9.13 | 6.35 | 8.83 | 6.81 | 17.42 | 8.26 | 8.85 | 9.57 | 9.51 | 10.09 | 9.09 | 6.92 | 11.19 | 10.41 | 12.13 | 14.70 | 8.35 | 36.77 | 2.07 | 10.93 | 3.21 | 7.48 | 8.82 |
| **O_3_** | 9.74 | -25.06** | -25.81** | -21.98* | -24.05* | -24.12 | -25.74* | -25.05 | -25.10** | -24.54* | -23.57* | -26.13** | -25.61* | -26.66** | -25.63** | -26.07** | -26.82** | -25.79** | -25.62** | -26.17** | -24.31* | -26.61* | -24.01* | -25.16* | -25.12** | -38.87** | -37.15** |
| **NO_2_** | 10.54 | 12.06 | 14.20 | 7.81 | 8.55 | 0.82 | 8.76 | 3.56 | 14.85 | 11.42 | 8.51 | 13.20 | 11.31 | 14.15 | 11.56 | 14.16 | 13.76 | 13.16 | 13.01 | 12.79 | 11.91 | 11.27 | 8.35 | 7.84 | -18.56 | 12.83 | 11.60 |
| **NO_X_** | 28.05 | 9.74 | 10.24 | 7.17 | 5.90 | -2.65 | 6.28 | -0.47 | 10.35 | 10.42 | 5.93 | 12.50 | 9.83 | 12.44 | 9.11 | 16.53 | 10.97 | 11.27 | 10.70 | 10.25 | 10.63 | 8.22 | 6.34 | 6.80 | -14.44 | 1.00 | 10.25 |

For explanation see Table S9.
