# Supplementary material for: Composition of PM Affects Acute Vascular Inflammatory and Coagulative Markers - The RAPTES Project
Source: PLoS One. 2013 Mar 13;8(3):e58944. doi: 10.1371/journal.pone.0058944 (PMC3596332; doi:10.1371/journal.pone.0058944)
Supplement: Table S19 — Two-pollutant models of associations between exposure to air pollution and percentage changes (25 h post-pre) in tPA/PAI-1 complex (not log-transformed; all sites). (DOC) [file pone.0058944.s020.doc]

**Table S19** Two-pollutant models of associations between exposure to air pollution and percentage changes (25h post-pre) in tPA/PAI-1 complex (not log-transformed; all sites).

|  | **IQR** | **A D J U S T M E N T P O L L U T A N T S** | | | | | | | | | | | | | | | | | | | | | | | | | |
| --- | --- | --- | --- | --- | --- | --- | --- | --- | --- | --- | --- | --- | --- | --- | --- | --- | --- | --- | --- | --- | --- | --- | --- | --- | --- | --- | --- |
|  |  | **PM_10_** | **PM_2.5_** | **PM_2.5-10_** | **PNC** | **Abs.^a^** | **EC (F)** | **EC (C)** | **OC (F)** | **OC (C)** | **Fe (tot)** | **Fe (sol)** | **Cu (tot)** | **Cu (sol)** | **Ni (tot)** | **Ni (sol)** | **V (tot)** | **V (sol)** | **End.** | **NO_3_^- a^** | **SO_4_^2- a^** | **OP^AA^** | **OP^GSH^** | **OP^TOTAL^** | **O_3_** | **NO_2_** | **NO_X_** |
| **PM_10_** | 13.50 | 1.01** | 1.05 | 0.96 | 0.94** | 0.00 | 0.28 | 1.89 | 1.17** | 1.15** | 1.46 | 1.24** | 2.19 | 1.36** | 2.00* | 1.04** | 0.53 | 1.02** | 1.00** | 1.00** | 1.08** | 1.77* | 1.91* | 2.07* | -1.18 | 0.85** | 0.82** |
| **PM_2.5_** | 11.54 | -0.08 | 2.25** | 0.82 | 2.12** | 0.19 | 0.65 | 2.06 | 2.82** | 2.44* | 1.14 | 2.70** | 2.38 | 2.82** | 2.67 | 2.25** | 1.06 | 2.27** | 2.20** | 2.27** | 2.27** | 3.19* | 2.50 | 2.92 | -1.89 | 1.81** | 1.78* |
| **PM_2.5_**-**_10_** | 8.23 | 0.05 | 0.64 | 0.95** | 0.86** | -0.06 | 0.19 | 1.62 | 1.02** | 1.03** | 1.57 | 1.15** | 1.89 | 1.26** | 1.90* | 0.99** | 0.47 | 0.95** | 0.94** | 0.94** | 1.04** | 1.44* | 1.99* | 1.83* | **-**1.13* | 0.81** | 0.76** |
| **PNC** | 32,906 | 8.46 | 9.13* | 8.18 | 10.33* | 2.99 | 3.61 | 7.32 | 10.03* | 10.34* | 7.55 | 9.67* | 7.91 | 9.07 | 9.30* | 10.15* | 7.75 | 9.87* | 10.19* | 12.62** | 12.63** | 7.96 | 7.70 | 7.79 | 3.59 | 5.03 | 5.36 |
| **Absorbance^a^** | 3.49 | 8.07 | 7.58 | 8.54 | 7.36** | 8.11** | 22.38 | 11.68* | 8.86** | 7.60** | 9.08 | 10.05** | 11.28* | 9.61** | 9.66** | 8.26** | 5.52 | 7.99** | 8.58** | 8.23** | 9.11** | 11.78** | 11.65** | 12.28** | -7.26 | 6.49** | 7.53** |
| **EC (F)** | 4.35 | 6.72 | 6.83 | 7.30 | 7.76** | -16.32 | 8.76** | 10.17 | 8.94** | 8.02** | 8.26 | 10.36** | 10.83 | 9.62** | 9.97* | 9.05** | 5.38 | 8.65** | 9.20** | 9.14** | 10.11** | 10.99* | 10.05* | 10.81* | -7.07 | 6.95** | 7.60** |
| **EC (C)** | 0.40 | -1.25 | 0.13 | -1.00 | 1.12** | -0.77 | -0.28 | 1.27** | 1.46** | 1.13* | -0.92 | 1.84** | 1.35 | 2.07** | 1.37 | 1.31** | 0.31 | 1.27** | 1.33** | 1.26** | 1.36** | 1.88 | 1.67 | 2.03 | **-**1.59* | 1.07** | 0.97* |
| **OC (F)** | 1.82 | -1.99 | -2.93 | -1.14 | 2.99 | -1.57 | -0.40 | -1.75 | 3.27 | 1.27 | -1.32 | 2.92 | -1.33 | 1.06 | -0.32 | 3.11 | 0.16 | 3.59 | 2.97 | 2.91 | 2.92 | -1.98 | -2.48 | -2.49 | -2.77 | 1.12 | 1.49 |
| **OC (C)** | 0.79 | -0.90 | -0.58 | -0.56 | 3.62* | 0.45 | 0.76 | 0.84 | 3.12 | 3.46* | 0.29 | 3.36* | 0.83 | 3.05 | 1.04 | 3.42* | 1.05 | 3.47* | 3.56* | 3.53* | 3.64* | 2.51 | 2.11 | 2.24 | -0.81 | 2.94 | 2.60 |
| **Fe (tot)** | 895.10 | -0.07 | 0.08 | -0.11 | 0.14** | -0.03 | 0.01 | 0.26 | 0.17** | 0.15* | 0.16** | 0.19** | 0.51 | 0.20** | 0.31* | 0.16** | 0.05 | 0.16** | 0.16** | 0.16** | 0.17** | 0.26* | 0.25 | 0.29 | -0.17 | 0.14** | 0.13** |
| **Fe (sol)** | 32.09 | -1.99 | -1.79 | -1.85 | 0.31 | -2.52 | -1.94 | -3.05 | 0.21 | 0.32 | -2.01 | 1.24 | -2.68 | -1.84 | -0.96 | 1.15 | -0.79 | 1.27 | 1.01 | 1.24 | 1.35 | -3.76 | -2.68 | -3.41 | -2.56 | 0.70 | -0.21 |
| **Cu (tot)** | 57.96 | -0.27 | -0.01 | -0.22 | 0.18** | -0.11 | -0.06 | -0.01 | 0.22** | 0.17 | -0.49 | 0.27** | 0.20** | 0.28** | 0.21 | 0.21** | 0.01 | 0.19** | 0.20** | 0.19** | 0.21** | 0.27 | 0.21 | 0.26 | -0.21 | 0.17** | 0.15* |
| **Cu (sol)** | 8.65 | -0.13 | -0.10 | -0.12 | 0.10 | -0.10 | -0.06 | -0.20 | 0.10 | 0.04 | -0.11 | 0.21 | -0.14 | 0.13 | -0.03 | 0.13 | -0.01 | 0.13 | 0.12 | 0.12 | 0.12 | -0.08 | -0.14 | -0.12 | -0.15 | 0.10 | 0.04 |
| **Ni (tot)** | 3.53 | -1.49 | -0.30 | -1.53 | 1.13** | -0.41 | -0.27 | -0.12 | 1.20* | 0.97 | -1.42 | 1.31** | -0.10 | 1.28* | 1.17** | 1.19** | -0.08 | 1.17** | 1.17** | 1.19** | 1.40** | 0.89 | 0.65 | 0.72 | -1.05 | 1.03* | 0.89 |
| **Ni (sol)** | 1.82 | -1.02 | -0.06 | -1.48 | -0.56 | -1.71 | -1.77 | -1.23 | 1.45 | 0.83 | -1.68 | 0.77 | -1.59 | 0.29 | -0.52 | 1.44 | -7.71 | 0.92 | 1.02 | 2.24 | 2.43 | 0.17 | -0.98 | -0.44 | -3.04 | 0.35 | 0.09 |
| **V (tot) ^b^** | 2.04 | 1.21 | 1.41 | 1.26 | 2.00** | 0.91 | 1.06 | 1.78 | 2.15** | 1.95** | 1.53 | 2.30** | 2.05 | 2.21** | 2.26* | 3.07** | 2.17** | 2.43** | 2.26** | 2.22** | 2.33** | 2.24* | 2.34 | 2.29* | -1.03 | 1.86** | 1.82** |
| **V (sol) ^b^** | 1.94 | 1.15 | 1.83 | 0.76 | 1.00 | 1.66 | 1.10 | 1.23 | 2.51 | 1.43 | 0.81 | 1.45 | 1.00 | 1.38 | 1.16 | 0.76 | -3.17 | 1.33 | 1.03 | 1.65 | 0.81 | 2.54 | 1.31 | 1.93 | -0.53 | 0.05 | 0.74 |
| **Endotoxin** | 0.19 | -0.01 | -0.03 | -0.01 | -0.01 | 0.03 | 0.03 | 0.03 | -0.04 | -0.05 | 0.03 | -0.03 | 0.02 | -0.02 | 0.00 | -0.04 | 0.03 | -0.04 | -0.06 | -0.07 | -0.06 | 0.00 | 0.00 | 0.00 | 0.05 | -0.01 | 0.00 |
| **NO_3_^- a^** | 5.19 | 0.96 | -0.20 | 1.67 | 3.57 | 2.21 | 2.68 | 1.55 | 0.81 | -0.22 | 1.82 | 1.66 | 1.59 | 1.19 | 1.91 | 2.03 | 2.23 | 1.80 | 2.13 | 1.79 | -0.32 | 2.75 | 2.77 | 2.75 | 0.68 | 0.16 | 1.43 |
| **SO_4_^2- a^** | 2.99 | 4.93* | 4.19 | 5.36* | 5.64* | 5.92** | 6.16** | 4.80 | 3.68 | 4.29 | 5.34* | 4.01 | 4.96* | 3.79 | 5.69* | 4.24 | 5.01* | 3.85 | 4.00 | 4.21 | 3.99 | 5.80* | 5.78* | 5.81* | 3.92 | 3.17 | 4.16 |
| **OP^AA^** | 19.08 | -0.29 | -0.17 | -0.21 | 0.22* | -0.20 | -0.13 | -0.18 | 0.29* | 0.15 | -0.25 | 0.43** | -0.14 | 0.31* | 0.06 | 0.24* | -0.02 | 0.24** | 0.24* | 0.23* | 0.26** | 0.24** | 0.00 | -0.26 | **-**0.33* | 0.21* | 0.18 |
| **OP^GSH^** | 15.53 | -0.27 | -0.05 | -0.32 | 0.19* | -0.15 | -0.06 | -0.10 | 0.26** | 0.13 | -0.19 | 0.30** | -0.03 | 0.31* | 0.10 | 0.21** | -0.03 | 0.20** | 0.20** | 0.20** | 0.22** | 0.21 | 0.20** | 0.21 | **-**0.28* | 0.18* | 0.16 |
| **OP^TOTAL^** | 38.71 | -0.39 | -0.12 | -0.35 | 0.24* | -0.23 | -0.12 | -0.22 | 0.32** | 0.16 | -0.32 | 0.42** | -0.12 | 0.38* | 0.11 | 0.26** | -0.03 | 0.26** | 0.26** | 0.25** | 0.27** | 0.52 | -0.01 | 0.26** | **-**0.38* | 0.23* | 0.20 |
| **O_3_** | 9.74 | **-**24.76** | **-**21.39** | **-**24.90** | **-**13.85** | **-**23.45** | **-**21.99** | **-**23.99** | **-**16.36** | **-**15.25** | **-**23.50** | **-**16.80** | **-**22.07** | **-**17.47** | **-**19.91** | **-**15.19** | **-**18.30** | **-**14.49** | **-**15.41** | **-**14.53** | **-**14.60** | **-**22.39** | **-**23.34** | **-**23.68** | **-**14.65** | **-**13.93** | **-**16.48** |
| **NO_2_** | 10.54 | 11.69* | 11.33* | 12.13** | 12.12* | 8.59 | 9.66 | 12.17* | 14.22** | 13.47** | 12.08* | 14.39** | 12.50** | 13.89** | 13.11** | 14.64** | 11.48* | 14.70** | 14.82** | 14.90** | 14.21** | 12.11* | 11.82* | 11.91* | 2.21 | 15.00** | 12.70 |
| **NO_X_** | 28.05 | 6.08 | 6.33 | 6.28 | 7.95 | 1.63 | 3.07 | 5.96 | 10.07* | 8.84* | 6.02 | 10.83** | 6.51 | 9.67* | 7.60 | 10.59** | 6.73 | 10.54** | 10.98** | 10.67** | 11.00** | 6.87 | 6.52 | 6.58 | -3.84 | 2.26 | 10.86** |

For explanation see Table S9.
